# Cancer Cell‐Secreted miR‐33a Reduces Stress Granule Formation by Targeting Polyamine Metabolism in Stroma to Promote Tumourigenesis

**DOI:** 10.1002/jev2.70153

**Published:** 2025-09-03

**Authors:** Sheng Hu, Xu Li, Qixin Hu, Chenyu Wang, Ao Hua, Gang Deng, Wenda Huang, Xiaoyu Fu, Haifeng Zhou, Xiaohui Zhang, Meixin Li, Juan Wu, Mingzhou Chen, Xiaolu Zhao, Lianyun Li, Zifu Li, Min Wu, Juanjuan Li, Wei Yan

**Affiliations:** ^1^ State Key Laboratory of Metabolism and Regulation in Complex Organisms, Hubei Provincial Research Center for Basic Biological Sciences, Hubei Key Laboratory of Cell Homeostasis, College of Life Sciences, TaiKang Center for Life and Medical Sciences, RNA Institute Wuhan University Wuhan China; ^2^ State Key Laboratory of Metabolism and Regulation in Complex Organisms, Hubei Key Laboratory of Cell Homeostasis, College of Life Sciences, Frontier Science Center for Immunology and Metabolism, TaiKang Center for Life and Medical Sciences Wuhan University Wuhan China; ^3^ National Engineering Research Center for Nanomedicine, College of Life Science and Technology Huazhong University of Science and Technology Wuhan China; ^4^ Department of Breast and Thyroid Surgery Renmin Hospital of Wuhan University Wuhan Hubei China; ^5^ Department of Pathology Renmin Hospital of Wuhan University Wuhan Hubei China

**Keywords:** epigenetic reprogramming, extracellular vesicles, polyamine metabolism, stress granules, tumour microenvironment

## Abstract

Tumour progression depends on the bidirectional interactions between cancer and stroma in the heterogeneous tumour microenvironment (TME) partially through extracellular vesicles (EVs). However, the secretary mechanism and biological effect of cancer cell derived EVs on tumour survival under starvation is poorly defined. Here, we identify cancer cells selectively secrete miR‐33a with the assistance of aconitase 1 (ACO1), an iron‐responsive RNA binding protein, under glucose starvation and lower iron level, which affiliates the binding capability of miR‐33a and ACO1. Exosomal miR‐33a suppresses putrescine biosynthesis by targeting AGMAT in cancer‐associated fibroblasts (CAFs) from tumour core region, where putrescine inhibits the expression of demethylase KDM5C. TIA1 gene, stress granule (SG) marker, is tightly regulated by miR‐33a/KDM5C/H3K4me3 axis and exosomal miR‐33a diminishes the formation of stromal SGs in CAFs. Collectively, our study reveals tumour selectively secretes miR‐33a‐5p through EVs to remodel the stromal SG formation and gain survival possibility for cancer cells in tumour core region, highlighting a novel regulatory mechanism of iron and nutrient level on EV secretion and the function of polyamine metabolism in reshaping epigenetic profiles.

## 1 Introduction

The metabolic heterogeneity of tumour microenvironment (TME) results from the unequal supply of oxygen and nutrients by the tumour vasculature across the tumour bulk (Anderson and Simon 2020; Helms et al. 2020). Therefore, metabolic reprogramming is one of the hallmarks of cancer to serve as an adaptive mechanism by which fast‐growing cancer cells adapt to their increasing energy demands (Solimini et al. 2007). Such responses not only include alterations to the metabolic and transcriptomic landscape, but also the epigenetic profile to cope with stress (Song and Grabocka 2023). Moreover, epigenetic regulation of gene expression is dysregulated in many cancers, including breast cancer (Zhu et al. 2012). T cell intracellular antigen‐1 (TIA‐1), an RNA binding protein, promotes the assembly of stress granules (SGs) by discrete cytoplasmic inclusions into stalled translation initiation complexes and dynamically recruits in cells subjected to environmental stress (Meyer et al. 2018; Kedersha et al. 1999). Several intrinsic pro‐tumourigenic signalling molecules have been shown to induce SG formation, such as the RAS oncogene and mTOR (Grabocka and Bar‐Sagi 2016; Fournier et al. 2013; Wippich et al. 2013). However, whether extrinsic signals derived from cancer cells regulates SGs in tumour stroma under stress is poorly defined.

Cancer progression and metastasis closely connected to TME (Quail and Joyce 2013). Tumour stroma consists of many different kinds of cell types including immune cells, extracellular matrix (ECM) and activated fibroblasts such as cancer associated fibroblasts (CAFs) (Kalluri and Zeisberg 2006). CAFs, the major components of stromal cells that surround cancer cells, functioning as mechanical support for the proliferation, survival and metastasis for cancer cells (Chen and Song 2019). CAFs facilitate creating ECM structure, metabolic reprogramming and immune reshaping of the TME with an impact on adaptive resistance to chemotherapy (Kay et al. 2022). There are multiple ways to achieve tumour‐stroma metabolic communication, such as extracellular vesicles (EVs) (Bertero et al. 2019).

EVs have been recognized as crucial signalling mediators by encapsulating bioactive cargoes, such as miRNAs and proteins to communicate with each other in TME (van Niel et al. 2018; Shah et al. 2018; Mathieu et al. 2019; van Niel et al. 2022). EV secretion and the specific loading of signalling factors in EVs contribute to organ development and tissue differentiation. Recycling Rab GTPase family members are very crucial for EV secretion, such as Rab11 and Rab35 (Linnemannstons et al. 2022). Moreover, some tetranucleotide sequences have been found in some specific cell types (Santangelo et al. 2016; Temoche‐Diaz et al. 2019; Villarroya‐Beltri et al. 2013). The presence of EV export (EXOmotifs) versus cellular retention (CELLmotifs) sequences contribute to EV sorting and cellular retention (Garcia‐Martin et al. 2022). Besides, CD63 has been reported in EV secretion and CD63 is post‐transcriptionally regulated by iron via the IRE‐IRP system. Excess iron induces CD63 expression and ferritin is transferred to CD63^+^ EVs (Yanatori et al. 2021). In this study, we report cancer cells secrete more miR‐33a‐5p to suppress the polyamine metabolism and thereby remodel the epigenetic profile in tumour stroma for survival under nutrient‐poor region. Our work reveals the contribution of EVs to the heterogeneity of TME and the capability for stress adaption of cancer cells, shedding light on the future therapeutic applications for cancer.

## 2 Materials and Methods

### 2.1 Cells and Constructs

Cell lines applied in this study were obtained from American Type Culture Collection (ATCC) including MDA‐MB‐231 (HTB‐26), MCF‐7 (HTB‐22), BT‐474 (HTB‐20), 4T1 (CRL‐2539), E0771 (CRL‐3461) and MCF‐10A (CRL‐10317), and the mouse embryonic fibroblast cell line NIH3T3 (CRL‐1658). The human or mice derived primary cancer associated fibroblasts denoted as hCAF or mCAF in this study were isolated as reported previously (Tsuyada et al. 2012), and were cultured in DMEM (Gibco, 11965092) containing 10% FBS and 1% penicillin/streptomycin. All cell lines were cultured in an atmosphere of 5% CO_2_ incubator at 37°C. MDA‐MB‐231/Rab27a KD cells were constructed using short hairpin RNA (shRNA) targeting Rab27a sequence CCAGTGTACTTTACCAATATA and the corresponding oligonucleotides were cloned into pLKO.1 puro vector (Addgene, 8453). Cells were transduced by the lentivirus and selected in puromycin. To knock out *hsa*‐miR‐33a‐5p gene in MDA‐MB‐231 using CRISPR–Cas9 genomic editing system, two single‐guide RNAs (sgRNAs; DNA sequences TTACGGCTTATTGGCCTAGG and GGAGCATCACCTGGTCTACG) predicted by the sgRNA Designer (https://portals.broadinstitute.org/gpp/public/analysis‐tools/sgrna‐design) were synthesized in DNA form and annealed into double strands treated with T4 polynucleotide kinase and inserted into the BsmbI‐digested lentiCRISPR v2 vector (Addgene, 52961). The two constructs were co‐transfected into MDA‐MB‐231 cells, and selected by puromycin. A similar strategy was used to construct 4T1/miR‐33 KO cells using sgRNAs (DNA sequences GGTGCAAACACATTTGCCCG and GAGGGCCTACCTAACCATCG). Monoclones were screened by genotyping PCR and confirmed by sequencing (Tsingke). 4T1/Rab27a KO cells using sgRNA (DNA sequence AGCGTCCCTGAAGAATGCAG). Monoclones were screened by western blot and confirmed by sequencing. To construct reporters for miR‐33a‐mediated regulation of AGMAT, a region encompassing partial coding region and 3’UTR of human *AGMAT* gene (primers 5′‐GCTTCTCCTATGAGGTGCTT and 5′‐TGGAAAAAGAAATAAACTAT) and the 3′UTR of mouse *Agmat* gene (primers 5′‐TGAGCCACCACTGCATGCT and 5′‐ATGCTGTGGAGACGGAATGC) were cloned by PCR and inserted into the XhoI/NotI sites of psiCHECK‐2 reporter vector (Promega, Cat# C8021) downstream of the Renilla luciferase (R‐Luc) gene. Site‐directed mutagenesis was used to mutate the following putative miR‐33a binding sites with corresponding primer sets: 5′‐ATCTTGCAATGCAAGGGCTACCCCATAATTATCAG and 5′‐TAGCCCTTGCATTGCAAGATGATGATTTGATTATG for human *AGMAT* site; 5′‐TCCTAAGGAGAGGCATGGCAGCCCCCGGT and 5′‐ACCGGGGGCTGCCATGCCTCTCCTTAGGA for mouse *Agmat* site. A similar strategy was used to construct reporters for polyamine‐mediated regulation of KDM5C, 5′UTR of human *KDM5C* gene (primers 5′‐GACAAACCAAGATGGCGGCG and 5′‐GGTGGGCCCGAGGTCTGGGCCA) were cloned by PCR and inserted into psiCHECK‐2 reporter vector upstream of the Renilla luciferase (R‐Luc) gene. The pLVX‐IRES‐ZsGreen1 expression plasmid (Takara, Cat# 632187) was used to generate the expression of human miR‐33a using primers 5′‐CATTTGCTCCAGCGGTTTGC and 5′‐CTGGGATGGCTGTGACTTG, 5′‐ATGCTGAGGCTGCTGAGGTCCAGCT and 5′‐TCAGACAGTTGTCACTTTGGGGAG for mouse *Agmat*, 5′‐ATGGAGGACGAGATGCCCAAG and 5′‐TCACTGGGTTTCATACCCTGC for human *TIA1*. All plasmid DNA was validated by sequencing.

### 2.2 EVs Isolation and Characterization

EVs were separated via ultracentrifugation as previously described (Fong et al. 2015). The medium from 90% confluent cells grown in EV‐free was collected and pre‐cleared by centrifugation at 500 × *g* for 15 min and then at 10,000 × *g* for 20 min. Tissue derived EVs were separated following the protocol established previously (Crescitelli et al. 2021). Specifically, a small (∼50 mg) piece of tissue was weighed and briefly sliced on dry ice and then incubated in 100 U/mL collagenase type I in HANKS solution at 37°C. The dissociated tissue was spun at 300 × *g* for 10 min at 4°C. The supernatant was transferred to a new tube and centrifuged at 2000 × *g* for 15 min at 4°C. Cell‐free supernatant was filtered through the 0.22 µm filter gently and slowly for further removal of cell debris and spun at 10,000 × *g* for 30 min at 4°C. EVs were pelleted by ultracentrifugation at 110,000 × *g* for 70 min, and resuspended in PBS for other experiments. Opti‐prep gradient for density separation was reported as previously described (Tauro et al. 2012). EVs were stored at −80°C and thawed on ice before use. For in vitro cell treatment, 2 µg of EVs based on protein measurement using BCA protein assay kit was added to 2 × 10^5^ recipient cells (Yan et al. 2022). EV size distribution and particle concentration were analysed using Flow NanoAnalyzer U30 (NanoFCM, Xiamen, China) according to the manufacturer's instructions. Briefly, the instrument calibrated for particle concentration to 10^7^–10^9^ particles/mL with 250 nm fluorescent silica nanospheres (NanoFCM Inc., QS2503) and for size distribution using Silica Nanospheres Cocktail (NanoFCM Inc., S16M‐Exo).

### 2.3 Isolating Mitochondria

For mitochondrial isolation, a rapid differential centrifugation reported in a previous study was used (Bogenhagen and Clayton 1974). MDA‐MB‐231 cells in the culture dishes were scraped off, collected into a centrifuge tube, and suspended in PBS. The suspended cells were lysed using a sonication method. The homogenate was spun down at 1000 × *g* for 2 min, and the supernatant was taken and spun down at 10,000 × *g* for 10 min. The pellet was washed by gently suspending with 1 mL of PBS after aspirating the supernatant, and then transferred to a new tube. The suspension was spun again at 10,000 × *g* for 10 min, and the pellet was lysed by 100 µL NP‐40 lysis buffer for 10 min on ice. Afterwards, the lysate was transferred to a new tube after spun down at 17,000 × *g* for 10 min.

### 2.4 Luciferase Reporter Assay

Both 231/WT and 231/miR‐33a cells were cultured in 24‐well plates and transfected with 0.5 µg psiCHECK‐2 reporter vector with either wild‐type or mutated AGMAT 3′UTR insertion using Lipofectamine 2000 (Invitrogen, 11668019) for 48 h. 293T cells were cultured in a 24‐well plate and transfected with 0.5 µg psiCHECK‐2 reporter plasmid with either wild‐type or mutated KDM5C 5’UTR insertion using Lipofectamine 2000 for 48 h, with polyamine (25 µM) treatment for 24 h. The luciferase assays were performed by Dual‐Luciferase Reporter Assay System (Promega, E1910). After discarding the culture medium, add 100 µL of Passive Lysis Buffer (PLB) to each well and shake for 15 min for lysis. Add 10 µL of the cell lysate to a luminometer tube containing 10 µL of LAR II and measure firefly luciferase luminescence. Add 10 µL of Stop & Glo Reagent and measure Renilla luciferase luminescence by GloMax 20/20 Luminometer (Promega, E5311).

### 2.5 MiRNA Mimic Treatment

Cells were cultured in 6‐well plates and transfected with miR‐33a‐5p mimic (Ambion, MC12410) or the negative mimic control (Ambion, 4464058) using Lipofectamine RNAiMAX (Invitrogen, 13778150) at a final concentration of 10 nM. After 24 and 48 h of incubation, the cells were collected for RNA and protein detection, respectively.

### 2.6 Lentivirus Package

HEK293T cells were transfected using Lipofectamine 2000 (Invitrogen, 11668019) with packaging plasmid: psPAX2 (Addgene, 12260) and pMD2.G (Addgene, 12259). Forty‐eight hours after transfection, lentiviral supernatant was harvested and filtered through a 0.45‐µm filter, and utilized for subsequent infection of MCF‐10A, MDA‐MB‐231, 4T1 and BALB/c mouse CAF cells (48 h incubation) together with 10 µg/mL polybrene (Beyotime, C0351) in the medium. Cells were selected based on puromycin selection or sorted by GFP^+^ cells with FACS.

### 2.7 Mass Spectrometry Imaging

Frozen tissue samples were fixed in three drops of distilled water during the cutting stage. The tissues were sectioned at 12 µm thickness using a Leica CM1950 cryostat (Leica Microsystems GmbH, Wetzlar, Germany) at −20°C. Afterwards, the tissue sections were placed in groups on electrically conductive slides coated with indium tin oxide (ITO), and the slides with tissue sections were dried in a vacuum desiccator for 30 min. Desiccated tissue sections mounted on ITO glass slides were sprayed using an HTX TM sprayer (Bruker Daltonics, Germany) with 15 mg/mL DHB (2,5‐dihydroxybenzoic acid), dissolved in 90%:10% Acetoni‐trile:water. The sprayer temperature was set to 60°C, with a flow rate of 0.12 mL/min, and a pressure of 5 psi. Twenty‐six passes of the matrix were applied to slides with 5 s of drying time between each pass. MALDI timsTOF MSI experiments were performed on a prototype Bruker timsTOF flex MS system (Bruker Daltonics, Bremen, Germany) equipped with a 10 kHz smartbeam 3D laser. Laser power was set to 60% and then fixed throughout the whole experiment. The mass spectra were acquired in positive mode. The mass spectra data were acquired over a mass range from m/z 50–1300 Da. The imaging spatial resolution was set to 50 µm for the tissue, and each spectrum consisted of 400 laser shots. MALDI mass spectra were normalized with the Root Mean Square, and the signal intensity in each image was shown as the normalized intensity. MS/MS fragmentations performed on the timsTOF flex MS system in the MS/MS mode were used for further detailed structural confirmation of the identified metabolites.

### 2.8 MiRNA Pulldown and RNA Binding Protein Immunoprecipitation

Cells were scraped and extracted with radioimmunoprecipitation assay buffer (100 µM PMSF, protease inhibitor cocktail and recombinant RNase Inhibitor) for 30 min on ice followed by centrifugation at 10,000 × *g*. The supernatant was transferred to new tubes. A total of 3 µg biotinylated miRNAs: wild‐type *hsa*‐miR‐33a‐5p, mutated *hsa*‐miR‐33a‐5p and scrambled miRNA (5′‐UUGUACUACACAAAAGUACUG‐3′) (Sangon Biotech) were pretreated at 90°C for 2 min followed by incubation on ice for 2 min and then added to annealing buffer for RNA oligos (Beyotime, R0051) for 20 min at room temperature. One milligram cell lysate was added to the preprocessing biotinylated miRNAs with continuous rotation at 4°C for 1 h. In parallel, 30 µL streptavidin magnetic beads (Beyotime, P2151) per sample were washed and resuspended in radioimmunoprecipitation assay buffer. Then streptavidin magnetic beads were added to samples overnight at 4°C under continuous rotation. After overnight incubation, magnetic beads were collected by magnet and completely washed briefly three times with radioimmunoprecipitation assay buffer and extra wash with PBS. All the buffers were containing recombinant RNase Inhibitor. The magnetic beads were resuspended by SDS‐PAGE sample loading buffer and got boiled for 10 min at 95°C for downstream western blot analysis. For immunoprecipitation, cells were fixed with 1% final formaldehyde and then quenched with 0.125 M of glycine for 5 min. Briefly, cells were harvested and lysed with RIP lysis buffer (50 mM Tris‐HCl, pH 7.4, 100 mM NaCl, 0.1% SDS, 0.5% sodium deoxycholate, 1% NP‐40, protease inhibitor cocktail and recombinant RNase Inhibitor). Cell debris were removed by centrifugation at 10,000 × *g* for 10 min at 4°C. One microgram of ACO1 antibody or immunoglobulin G antibody was added to supernatant and incubated for 2 h at 4°C with gentle rotation. Thirty microlitres pre‐cleared Protein G‐Agarose (Roche, 11719416001) was added to samples followed by gentle rotation at 4°C overnight. On day 2, the beads were precipitated at 500 × *g* for 1 min at 4°C. The supernatant was aspirated off and a total of three washed with wash buffer (50 mM Tris‐HCl, pH 7.4, 150 mM NaCl, 1 mM MgCl_2_, 0.5% NP‐40 and recombinant RNase Inhibitor) was required. A total of 150 µL Proteinase K buffer (Wash buffer, 1% SDS and 10% Proteinase K) was added per sample for resuspension. Samples were then incubated at 55°C for 30 min. Biotin‐miR‐33a‐5p with 5′ biotin (5′GUGCAUUGUAGUUGCAUUGCA) and Biotin‐miR‐33a‐5p‐mut with 5′ biotin (5′GUGCAUUGUAGUUGGUAACGA) were purchased from Sangon Biotech (R15817).

### 2.9 MicroScale Thermophoresis (MST) Assay

To test the binding affinity between putrescine and the 5’UTR of KDM5C mRNA, Cy5‐labelled RNA oligo corresponding to the wild‐type (WT) 5′UTR of KDM5C mRNA (5′‐UGGCGAAGGCUGCGGUACGACGGCCACACGCC‐3′) and mutant (mut) 5′UTR of KDM5C mRNA (5′‐UGGCGUGGGCUGCGGUACGACGGCCACACGCC‐3′), which had the similar secondary structure at the predicted putrescine binding sites were synthesized with HPLC purification (Sangon Biotech, China). The Cy5‐labelled RNA sequences were diluted in DEPC water to a stock concentration of 40 nM. A 1 mM putrescine solution was prepared in DEPC water, followed by a 16‐step serial dilution at a 1:2 ratio while maintaining its binding status. The MST assay was performed by monolith NT.115 with 40% of excitation and medium of IR laser power. After pre‐test and 950 binding checks, the mixture with a series of ligand concentration was introduced into the capillary (MO‐951 K022‐SP, Monolith Premium Capillary) by siphoning and then the binding affinity was tested. All consumables used in this experiment are RNase‐free to prevent RNA degradation.

### 2.10 Molecular Generation and Docking

The secondary and tertiary structure of *hsa*‐miR‐33a‐5p molecule were predicted by RNAfold (http://rna.tbi.univie.ac.at/) and 3dRNA/DNA (http://biophy.hust.edu.cn/new/3dRNA/create). The structure with the lowest score in all five candidates was chosen for the next docking program. The structure of human ACO1 proteins was obtained from AlphaFold Protein Structure Database (https://alphafold.com/) based on PDB 2B3X. The *hsa*‐miR‐33a‐5p molecule was docked with ACO1 using hDock Server (http://hdock.phys.hust.edu.cn/). All structures and docking results shown in the figures were generated using PyMOL 2.5.2.

### 2.11 In Situ Hybridization and Immunohistochemistry

Formalin‐fixed, paraffin‐embedded tissues were cut into 4–6 µm sections. Sections were evaluated by immunohistochemistry (IHC) and in situ hybridization (ISH) analysis following previously reported protocol (Fong et al. 2015). For brightfield images, slides were scanned by Aperio versa slide scanner (Leica) and images were obtained with Aperio ImageScope software (Leica) and analysed by ImageJ. The immunohistochemical staining results of intensity were scored into four grades: 0, negative; 1, weak; 2, moderate and 3, strong. The frequency of positive cells was defined as follows: 0, less than 5%; 1, 5% to 25%; 2, 26% to 50%; 3, 51% to 75%; and 4, greater than 75%. The intensity score was multiplied by the frequency score to obtain the final score. When the staining was heterogeneous, we scored it as follow: each component was scored independently and summed for the results. For fluorescent images, images were captured by the Leica SP8 confocal microscope and analysed with LAS X software (Leica). The *hsa*‐miR‐33a‐5p probe with 5′ DIG and 3′DIG (5′UGCAAUGCAACUACAAUGCAC3′) was purchased from Sangon Biotech (Order number: R11929).

### 2.12 Multiplexed Immunohistochemistry

Multiplexed Tyramide Signal Amplification (TSA) immunohistochemistry staining was performed using Panovue 4‐plex IHC kit (Panovue, 10001100020). Four micrometres sections of FFPE tumours were cut and mounted on charged slides. Dewaxing and heat induced epitope retrieval of slides was performed using citrate antigen retrieval solution for 20 min at 100°C. Endogenous peroxidase was blocked for 10 min and the slides further blocked with 10% w/v BSA in TBST. AGMAT (Santa Cruz, sc‐166414), Putrescine (FineTest, FNab09929), KDM5C (Affinity, DF13631), Smooth muscle actin (Proteintech, 14395‐1‐AP), MPO (Proteintech, 22225‐1‐AP) and CD68 (Abclonal, A13286) antibodies were sequentially applied, followed by horseradish peroxidase‐conjugated secondary antibody incubation and tyramide signal amplification. Following labelling with TSA, antibodies were removed using a heat stripping step in citrate antigen retrieval solution for 10 min at 100°C. This was not applied following application of the third antibody. Nuclei were stained with DAPI after all the antigens above had been labelled. The stained slides were scanned to obtain multispectral images using Thunder Imager DMi8 (Leica) and analysed using LAS X software (Leica).

### 2.13 Electron Microscopy

EV pellets were resuspended in PBS buffer filtered through a 0.22 µm filter. EVs were gently dropped on the copper grids for 1 min and then stained using 2% uranyl acetate (SPI‐Chem, #02624‐AB) for 30 s at room temperature. For immunogold staining for ACO1, EVs were isolated by ultracentrifugation as described and dropped on the nickel grids covered with carbon film. All processes should be carefully handled to keep the samples from dry. EVs were blocked in PBS buffer containing 50 mM glycine, 1% BSA, 0.2% Tween‐20 and 0.05% Triton X‐100 for 10 min. After blocking, EVs were incubated with ACO1 antibody (Proteintech, 12406‐1‐AP) for 30 min at room temperature. After washing with PBS three times for 1 min, 10‐nm colloidal gold conjugated protein A (Boster, GA1054) was incubated for 30 min at room temperature. Samples were washed with PBS three times for 1 min followed by staining with 2% uranyl acetate as described above. The grids were examined with JEOL JEM‐1400 Plus transmission electron microscope, and images were analysed by GATAN GMS 3 digital micrograph software.

### 2.14 RNA Isolation and RT‐qPCR

Total RNA was harvested from cultured cells or tissues using the TRIzol reagent (Invitrogen, 15596026) followed by cDNA synthesis using the MonScript RTIII Super Mix with dsDNase kit (Monad Biotech, MR05201). A CFX Connect real‐time PCR system (Bio‐Rad Laboratories) was used to perform quantitative real‐time PCR (RT‐qPCR) of cDNA samples using MonAmp ChemoHS qPCR Mix (Monad Biotech, MQ00401). The exosomal RNAs were extracted by TRIzol LS reagent (Invitrogen,10296028). EVs resuspended in 1 mL TRIzol LS and then incubated at room temperature for 10 min. A total of 200 µL of chloroform was added, gently mixed, and incubated for 10 min on ice. After incubation, microcentrifuge tubes were centrifuged for 10 min at 12,000 × *g* at 4°C. The aqueous phase was transferred to new tubes, an equal volume of isopropanol and 1 µL glycogen (Roche, 10901393001) were added, and mixed with incubation at −20°C overnight. Samples were centrifuged at 12,000 × *g* for 15 min at 4°C. Aspirated off liquid and added 500 µL of cold 75% ethanol to wash the pellet. The RNA pellet was air dried for a few minutes at room temperature and subsequently resuspended in 20 µL of RNase‐free water. The cellular or exosomal miRNA was reverse transcribed according to the miScript II RT Kit (QIAGEN, 218161) and measured using miScript SYBR Green PCR Kit (QIAGEN, 218073). The primers for miR‐33a‐5p, miR‐16 and U6 were purchased from Tiangen (Cat# CD201‐0362, CD201‐0235 and CD201‐0145). Data were normalized to levels of U6 or miR‐16‐5p. All the primers were shown in Table S1.

### 2.15 Western Blot Analysis

Cells were harvested and extracted with NP‐40 buffer (50 mM Tris‐HCl, pH 7.4, 150 mM NaCl, EDTA, 1% Nonidet P‐40, phosphatase inhibitor and protease inhibitor) for SDS‐PAGE. Approximately 20 mg of tissues were ground with radioimmunoprecipitation assay buffer (50 mM Tris‐HCl, pH 7.4, 150 mM NaCl, 1% Triton X‐100, 1% sodium deoxycholate, 0.1% SDS, sodium orthovanadate, sodium fluoride, EDTA and leupeptin). Samples were centrifuged for 10 min at 12,000 × *g* at 4°C. The supernatant was transferred to new tubes. Histones were extracted from cells and tumour tissues by following the protocol established previously (Shechter et al. 2007). Briefly, the harvested cells and tissue samples were lysed in NETN buffer (20 mM Tris‐Cl, pH 8.0, 1 mM EDTA, 0.5% NP‐40, 500 mM NaCl, sodium orthovanadate, sodium fluoride and leupeptin) on ice for 30 min, and then centrifuged for 10 min at 12,000 × *g* at 4°C. And the pellet was washed using NETN buffer two times, followed by centrifugation at 12,000 × *g*, 4°C, for 10 min each time. Then the pellet was suspended in 0.2 M HCl and incubated on ice for 30 min, and samples were centrifuged at 12,000 × *g*, 4°C, for 15 min. The histone‐containing supernatant was transferred into a new tube and neutralized using 1.0 M Tris‐HCl, pH 8.0. Protein concentration was detected using a BCA Protein Assay Kit (Thermo Fisher Scientific). SDS‐PAGE sample loading buffer was added to the protein lysates and boiled for 10 min at 95°C. The proteins were resolved by SDS‐PAGE, electrophoretically transferred to PVDF membranes, and blocked in 5% skim milk. Blocking buffer was then removed and washed using TBST buffer for primary antibodies incubation at 4°C overnight. The membranes were washed 3 × 10 min with TBST, and incubated with horseradish peroxidase‐conjugated anti‐mouse or rabbit secondary antibody (Thermo Fisher Scientific, 31430, 31460) for 2 h. The secondary antibody was removed by washing 3 × 10 min with TBST. The membranes were incubated with West Femto Maximum Sensitivity Substrate (Thermo Fisher Scientific) for the detection of the immunoreactive bands. All the antibodies were shown in Table S2.

### 2.16 Isolation and Proteomic Characterization of Cell and Tissue‐Derived EVs

A small (∼50 mg) piece of tissue was weighed and briefly sliced on dry ice and then incubated in 100 U/mL collagenase type I in HANKS solution at 37°C. The dissociated tissue was spun at 300 × *g* for 10 min at 4°C. The supernatant of cell culture medium (20 × 15 cm dished of ‐G or complete medium cultured for 24 h) was collect and spun at 500 × *g* for 15 min at 4°C. The supernatant above‐mentioned was transferred to a new tube and centrifuged at 2000 × *g* for 20 min at 4°C. Cell‐free supernatant was filtered through the 0.22 µm filter gently and slowly for further removal of cell debris and spun at 10,000 × *g* for 30 min at 4°C. EVs were pelleted by ultracentrifugation at 110,000 × *g* for 70 min, and resuspended in PBS. Added an appropriate amount of protein lysate (8 M urea, 1% SDS), with protease inhibitor to inhibit protease activity. The mixture was treated by ultrasound for 2 min at a low temperature, following splitting for 30 min. After centrifugation at 12,000 × *g* at 4°C for 30 min, the concentration of protein supernatant was determined by the Bicinchoninic acid (BCA) method by Pierce BCA Protein Assay Kit (Thermo Fisher, USA). Protein quantification was performed according to the kit protocol. Added TEAB (Triethylammonium bicarbonate buffer) into 100 µg protein samples to the final TEAB concentration of 100 mM. Then added, TCEP (tris (2‐carboxyethyl) phosphine) to the final concentration of 10 mM and reacted for 60 min at 37°C. Following, add IAM (Iodoacetamide) to the final concentration of 40 mM and react for 40 min at room temperature under dark conditions. Added a certain percentage (acetone: sample v/v = 6:1) of pre‐cooled acetone to each sample and settled for 4 h at −20°C. After centrifugal for 20 min at 10,000 × *g*, the sediment was collected and add 100 µL 100 mM TEAB solution to dissolve. Finally, the mixture was digested with trypsin overnight at 37°C added at a 1:50 trypsin‐to‐protein mass ratio. The peptides were vacuum dried and then resuspended with 0.1% TFA. Samples were desalted with HLB and vacuum dried. Peptide concentrations were determined by peptide quantification kit (Thermo Fisher, Cat#23275). Loading buffer was added to each tube to prepare samples for mass spectrometry analysis, and the concentration of each sample was 0.25 µg/µL. Trypsin‐digested peptides were analysed by an EASY nLC‐1200 system (Thermo Fisher, USA) coupled with a timsTOF Pro2 (Bruker, Germany) mass spectrometer at Majorbio Bio‐Pharm Technology Co. Ltd. (Shanghai, China). Briefly, the C18‐reversed phase column (75 µm × 25 cm, Ionopticks, USA) was equilibrated with solvent A (A:2% ACN with 0.1% formic acid) and solvent B (B: 80% ACN with 0.1% formic acid). The peptides were eluted using the following gradient: 0–45 min, 3%–28% B; 45–50 min, 28%–44% B; 50–55 min, 44%–90% B; 55–60 min, 90%–90% B. The tryptic peptides were separated at a flow rate of 250 nL/min. Peptides were separated by an ultrahigh‐performance liquid phase system subjected to a capillary ion source and then analyzed by timsTOF Pro2 (Bruker, Germany), and the electrospray voltage was 1.5 kV. The peptide parent ions and their secondary fragments were detected and analysed using high‐resolution TOF. The secondary MS scanning range was 100–1700 m/z. Data acquisition on the timsTOF Pro2 was collected using the parallel accumulation serial fragmentation (PASEF) acquisition mode. After the first MS stage, the second MS stage (charge number of the parent ions was 0–5) was recorded using the 10 PASEF mode. A dynamic exclusion time of 24 s was used for the MS/MS scan. Partial proteomic data were shown in Tables S3 and S6.

### 2.17 RNA Sequencing and Analysis

RNA sequencing was performed by Majorbio (Shanghai, China) and transcriptome library was prepared following TruSeq RNA sample preparation Kit from Illumina (San Diego, CA) using 1 µg of total RNA. Libraries were size selected for cDNA target fragments of 300 bp through 2% Low Range Ultra Agarose followed by PCR amplified using Phusion DNA polymerase (NEB) for 15 PCR cycles. After quantified by TBS380, the paired‐end RNA‐seq sequencing library was sequenced with the Illumina HiSeq xten/NovaSeq 6000 sequencer (2 × 150 bp read length). The raw paired end reads were trimmed and quality controlled by SeqPrep (https://github.com/jstjohn/SeqPrep) and Sickle (https://github.com/najoshi/sickle) with default parameters. Then clean reads were separately aligned to reference genome with orientation mode using HISAT2 (https://daehwankimlab.github.io/hisat2) software. The mapped reads of each sample were assembled by StringTie (https://ccb.jhu.edu/software/stringtie/index.shtml?%20t=example) in a reference‐based approach. The expression level of each transcript was calculated according to the transcripts per million reads (TPM) method. RSEM (http://deweylab.biostat.wisc.edu/rsem/) was used to quantify gene abundances. Eventually, differential expression analysis was performed using the DESeq2.

### 2.18 Small RNA Deep Sequencing and Bioinformatics Analysis

Small RNA sequencing was performed by Majorbio (Shanghai, China) and sequencing libraries were generated using NEBNext Multiplex Small RNA Library Prep Set for Illumina (NEB) following manufacturer's recommendations and index codes were added to attribute sequences to each sample. Library quality was assessed on the Agilent Bioanalyzer 2100 system using DNA High Sensitivity Chips. After cluster generation, the library preparations were sequenced on an Illumina platform and 50 bp single‐end reads were generated. The raw paired end reads were trimmed and quality controlled by SeqPrep (https://github.com/jstjohn/SeqPrep) and Sickle (https://github.com/najoshi/sickle) with default parameters. Then clean reads were separately aligned to reference genome with orientation mode using HIASAT (https://ccb.jhu.edu/software/hisat2/index.shtml) software. The mapped reads of each sample were assembled by StringTie (https://ccb.jhu.edu/software/stringtie/index.shtml?t=example) in a reference‐based approach. Low‐quality bases (Sanger base quality of < 20) of the 3′ end were trimmed using in‐house perl scripts, and then the sequencing adapters were removed with the fastx toolkit software (http://hannonlab.cshl.edu/fastx_toolkit/). All identical sequences of sizes ranging from 18 to 32 nt were counted and eliminated from the initial data set. The assembled unique sequences were used for a BLAST search of the Rfam database, version 10.1 (http://rfam.sanger.ac.uk/), to remove non‐miRNA sequences (rRNA, tRNA, snoRNA, etc.). Bowtie2 (http://sourceforge.net/projects/bowtie‐bio/files/) was used to annotate the chromosomal location against the reference genome data. Through a BLAST search of the miRbase, version 21.0 (http://www.mirbase.org/), the highly matched sequences were used to count and analyse the known miRNA expression profile. The characteristics of hairpin structure of miRNA precursor could be used to predict novel miRNA. Mireap or miRDeep2 were used to predict novel miRNA, and the Dicer cleavage site and the minimum free energy of the small RNA tags were unannotated in the former steps. Meanwhile, in‐house scripts were used to obtain the identified miRNA base bias on the first position with certain length and on each position of all identified miRNA. The expression level of each miRNA was calculated according to the transcripts TPM method. Significant differently expressed (DE) miRNAs were extracted with |log2FC| > 1 and FDR < 0.05 by DEseq2. Partial results were shown in Table S4.

### 2.19 ChIP Assay and ChIP‐Sequencing

CAF cells (5 × 10^6^) plated into a 10 cm dish were cross‐linked for 10 min at room temperature with 1% final formaldehyde and then quenched with 0.125 M of glycine for 5 min. Aspirated off the supernatant and added PBS to wash the cells. The cross‐linked cells were harvested with PBS and then centrifuged for 5 min at 500 × *g*. Supernatant was discarded and the precipitates were lysed with lysis buffer (50 mM Tris‐HCl, pH 8.0, 0.5% SDS, 5 mM EDTA). After centrifugation at 10,000 × *g* for 2 min at 4°C, the pellet was washed once with digestion buffer (50 mM Tris‐HCl, pH 7.6, 1 mM CaCl_2_, 0.2% triton X‐100), incubated in 500 µL digestion buffer with 3 µL MNase (NEB, M0247S) at 37°C for 20 min and quenched with 5 µL of 0.5 M EDTA. Samples were sonicated briefly and the supernatant was transferred to new tubes after centrifugation. A total of 5 µL supernatant, 5 µL TE buffer (10 mM Tris‐HCl, pH 8.0, 1 mM EDTA) and 1 µL Proteinase K (20 mg/mL) were used to check the DNA fragments of about 200 to 800 bp. Immunoprecipitation was performed with 100 µL sheared chromatin samples, 1 µg antibody, 50 µL Protein G‐Agarose (Roche, 11719416001) and 350 µL dilution buffer (20 mM Tris‐HCl, pH 8.0, 150 mM NaCl, 2 mM EDTA, 1% triton X‐100) on rocker overnight at 4°C. The next day, the immunoprecipitated product were collected by centrifugation at 500 × *g* for 1 min at 4°C and washed with three sequential wash: wash buffer I (20 mM Tris‐HCl, pH 8.0, 150 mM NaCl, 2 mM EDTA, 1% triton X‐100, 0.1% SDS), wash buffer II (20 mM Tris‐HCl, pH 8.0, 500 mM NaCl, 2 mM EDTA, 1% triton X‐100, 0.1% SDS), wash buffer III (10 mM Tris‐HCl, pH 8.0, 0.25 M LiCl, 1 mM EDTA, 1% sodium deoxycholate, 1% NP‐40) and followed by another two wash with TE buffer. The pellets were eluted twice with 100 µL elution buffer (1% SDS, 0.1 M NaHCO3, 1 µL 20 mg/mL Proteinase K) at room temperature. Samples were incubated at 65°C overnight and then purified with DNA purification kit (TIANGEN, DP214‐03).

ChIP‐seq libraries were constructed with ChIP and input DNA using VATHS Universal DNA Library Prep Kit for Illumina (Vazyme, ND606). Briefly, 50 µL of DNA (8–10 ng) was end‐repaired for dA tailing, followed by adaptor ligation. Each adaptor was marked with a barcode of 8 bp DNA. Adaptor‐ligated DNA was purified by AMPure XP beads (1:0.6) and then amplified by PCR of nine cycles with the primer matching with adaptor universal part. Amplified DNA was purified again using AMPure XP beads (1:0.9) in 20 µL EB elution buffer. For multiplexing, libraries with different barcode were mixed with equal molar quantities (30–50 million reads per library). Libraries were sequenced by Illumina Nova‐seq platform with pair‐end reads of 150 bp. The DNA sequencing information of ChIP‐seq Input samples was considered as the corresponding Whole Genome Sequencing (WGS) data. Before the immunoprecipitation procedure in ChIP assay, about 10% of the sonicated lysate supernatants were taken as the input samples, which contained fragmented genomic DNA (around 150 bp length) and binding proteins. Input sample was added with elution buffer to 100 µL, incubated at 65°C for 6 h, and then purified with DNA purification kit (TIANGEN, DP214‐03). The aim of this procedure is to remove DNA‐binding proteins and get pure DNA. VATHS Universal DNA Library Prep Kit for Illumina (Vazyme, ND606) was used to prepare libraries for genomic DNA, and the libraries were sequenced by the Illumina Nova‐seq platform with pair‐end reads of 150 bp.

### 2.20 ChIP‐seq Data Processing

For ChIP‐seq analysis, Fastqc (https://www.bioinformatics.babraham.ac.uk/projects/fastqc/) was used for raw data quality control. Cutadapt (https://cutadapt.readthedocs.io/en/stable/) was used to remove law quality bases and library adaptor contamination (cutadapt ‐a AGATCGGAAGAGCACACGTCTGAACTCCAGTCAC ‐A AGATCGGAAGAGCGTCGTGTAGGGAAAGAGTGT ‐u 5 ‐u ‐10 ‐U 5 ‐U ‐10 ‐m 30). After data filter, quality control of clean reads was performed by FastQC again. Bowtie2 (version 2.3.5.1) (http://sourceforge.net/projects/bowtie‐bio/files/) was used for data mapping to mouse reference genome mm10. Samtools (version1.4.1) (http://www.htslib.org) was used to sort BAM file and filter duplicate reads. Only unique mapped reads were accepted for further analysis. MACS2 (version2.1.1.2) (https://pypi.org/project/MACS2/) was used for ChIP‐seq peaks calling with p value cut‐off 1e‐8. Then HOMER annotate Peaks.pl (https://www.biostars.org/p/9475396/) was used to annotate ChIP‐seq peaks compared to reference genome mm10. We calculated the normalized RPKM as the ChIP‐seq signal in specific region. Briefly, ChIP‐seq reads aligning to the region were extended by 100 bp and the density of reads per bp was calculated using Bedtools (version 2.29.0). The density of reads in each region was normalized by reads per kilobase of bin per million mapped reads (RPKM). Partial results were shown in Table S5.

### 2.21 Targeted LC‐MS/MS

A total of 200 µL of H_2_O containing the internal standard succinate‐1,4‐^13^C_2_ (Sigma‐Aldrich, 485349) was added to the cell pellets. The cell suspension was vortexed for 30 s and sonicated on ice for 5 min, followed by adding 200 µL of 10% trichloroacetic acid (w/v) to precipitate proteins. The mixture was then centrifuged at 13,000 × rpm for 15 min at 4°C and the supernatant was transferred into a clean tube to dryness using a speed vac (Labconco, USA). The tumour tissue was weighed and homogenized in 200 µL H_2_O using a homogenizer (Automatic tissue grinder, Shanghai Jingxin Industry). A total of 200 µL of 10% trichloroacetic acid (w/v) with internal standard was added to the homogenized solution, vortexed for 30 s, and sonicated on ice for 10 min. The mixture was frozen in liquid nitrogen, thawed at room temperature and sonicated twice. The samples were centrifuged at 13,000 × rpm for 15 min at 4°C and the supernatant was transferred into a clean tube to dryness using a speed vac (Labconco, USA). The dried extract was redissolved in 10% acetonitrile and analysed by ultraperformance liquid chromatography with tandem mass spectrometry (UPLC–MS/MS) conducted on a Waters Acquity UPLC‐system coupled with 5500 QTRAP system (SCIEX). Chromatographic separation was achieved on a Waters Acquity UPLC BEH C18 Column (2.1 mm × 100 mm, 1.7 µm, Waters) using a flow rate of 0.2 mL/min at 40°C during a 10 min gradient (0–1 min from 1% B to 5% B, 1–3 min 5% B, 3–5 min from 5% B to 95% B, 5–10 min 95% B), using buffer A (2 mM ammonium acetate in 10% (v/v) acetonitrile) and buffer B (0.4% (v/v) acetic acid in 10% (v/v) acetonitrile). Mass spectrometry was operated in a polarity switching mode with electrospray source voltage set to +5000 V in positive mode and −4500 V in negative mode. The analytes were monitored in multiple reaction monitoring (MRM) mode using the precursor‐to‐product ion transitions of m/z 89.1 → 72.1 for putrescine (Sigma‐Aldrich, 51799), m/z 146.2 → 72.0 for spermidine (Sigma‐Aldrich, S2626), m/z 203.2 → 112.1 for spermine (Sigma‐Aldrich, S3256) and m/z 118.9 → 73.8 for succinate‐1,4‐^13^C_2_(IS). Collision energy was 13.3 eV for putrescine, 25.0 eV for spermidine, 29.9 eV for spermine and −16.3 eV for IS, respectively. Peak determination and area integration were performed using Analyst 1.7.1 (SCIEX) and SCIEX OS 1.4.0 software (SCIEX).

### 2.22 LC‐MS/MS

The coomassie‐stained SDS‐PAGE gel pieces were destained with a solution of 50 mM ammonium bicarbonate in 50% acetonitrile (1:1, vol/vol) and reduced by 10 mM DTT at 56°C for 60 min, followed by alkylation with 55 mM iodoacetamide at room temperature for 45 min in the dark. Gel pieces were dehydrated in acetonitrile, rehydrated in 10 mM ammonium bicarbonate and treated with trypsin (Promega) at an enzyme/substrate ratio of 1:50 (w/w) at 37°C overnight. Peptides were extracted from the gel pieces with 50% acetonitrile in 5% formic acid. The peptides were loaded on C18 stage tips twice and were desalted with FA then dried using a speed‐vac to complete dryness. An analytical column (75 µm inner diameter) packed with reversed‐phase Repro‐Sil Pur C18‐AQ 1.9 µm resin was prepared for sample loading. The dried samples were resuspended in 10 µL of LC‐MS buffer A (0.1% FA), transferred 5 µL to autosampler vials, and then injected 1 µL onto an Easy‐nano LC system (Thermo) coupled online with Q Exactive HF mass spectrometer (Thermo Scientific, San Jose, CA). The HPLC gradient was set as follows: 2%–5% Solvent B (0.1% FA in ACN) over 1 min, 5%–35% B over 20 min, 35%–100% B over 2 min, 100% B for 5 min at a flow‐rate of 200 nL/min. For data‐dependent acquisition (DDA), full‐MS (m/z 350–1600) were acquired in the orbitrap with a resolution of 60,000 and AGC target of 1e6. For MS/MS, the top 12 most abundant ions were automatically selected in each MS scan and fragmented in HCD mode at a resolution of 30,000 and AGC target of 1e5. Normalized collision energy (NCE) was set to 28. The LC–MS/MS data were processed and searched against the human protein database downloaded from Uniprot database using Proteome Discoverer (Version 2.5) for protein identification. A fully trypsin specific search was chosen as the enzyme and a maximum of two missed cleavages were allowed in the process. Dynamic modifications included were oxidation at methionine, phosphorylation at serine, threonine and tyrosine residues, and hydroxylation at lysine and proline residues. Fixed modification of carbamidomethyl at cysteine was chosen. The MS and MS/MS results were searched with a peptide ion mass tolerance of 10 ppm and a fragment ion mass tolerance of 0.02 Da.

### 2.23 Mice and Orthotopic Models

Animal experiments in this study were approved by and performed in accordance with the institutional animal care and use committee (IACUC) at the College of Life Science, Wuhan University (WDSKY0202008). Six‐eight weeks old female NOD/SCID/IL2Rγ‐null (NSG) mice were purchased from Shanghai Model Organisms Center (for MDA‐MB‐231 xenograft model) or female BALB/c mice purchased from Center for Disease Control (CDC; Hubei, China) (for 4T1 xenograft model) and 6‐weeks old male BALB/c‐Nude mice purchased from GemPharmatech corporation (for Hep3B, SW480 and A549 xenograft models) were used in this study. Mice were maintained under specific pathogen‐free conditions and housed four to five mice per cage at a 12 h light/dark cycle at a relative humidity of 30%–70% and room temperature of 22.2 ± 1.1°C, and were allowed free access to food and water. MDA‐MB‐231 xenograft models were established by injecting orthotopically 2 × 10^5^ WT cells or miR‐33a KO cells with an equal volume of Matrigel (Corning, 356234) into the fourth pair of mammary fat pad. 4T1 xenograft models were established by injecting 2 × 10^5^ WT cells, miR‐33 KO cells either alone or mixed with 1 × 10^6^ CAFs or CAFs stably expressing AGMAT/TIA1. To obtain fibroblast‐conditional knockout mice (*eIF5A^F−/−^*), the floxed eIF5A mice were crossed with PDGFRα‐cre transgenic mice. Xenograft tumours were established by injecting 1 × 10^6^ of E0771 cells mixed with Matrigel into the No.4 mammary fat pad. Hep3B, SW480 and A549 xenograft models were established by subcutaneously injecting 5 × 10^6^ into the right flank of each mouse, respectively. For ferric carboxymaltose (FeCM) injection into tumour core model, FeCM solution (10 g/L) was injected into the region of tumour core every other day at 30 µL per tumour and was administered seven times over a 12‐day period. For glucose injection into tumour, glucose solution (1 g/L) was injected into the region of tumour core every other day at 25 µL per tumour after 3 weeks and was administered seven times over 12 days. For polyamine (putrescine, spermidine, spermine) injection into tumour core region, putrescine, spermidine or spermine solution (25 µM) was injected into the region of tumour core every other day at 25 µL per tumour after ~3 weeks and was administered seven times over a 12‐day period. Tumour volume was assessed by the formula (1/2 *Lw* ^2^), where *L* is the longer diameter and *w* represents the shorter diameter. At the beginning of each experiment, mice were randomly assigned to each group.

### 2.24 Human Specimens

Archived samples from patients with cancer and health controls applied in this study were collected in accordance with the Clinical Research Ethical Committee of Renmin Hospital of Wuhan University. All participants provided written informed consent. Unstained paraffin‐embedded tissue sections and fresh tissues of breast cancer patients were obtained voluntary patient consent from Renmin Hospital of Wuhan University. A total of 500 µL of serum EVs were isolated using qEVoriginal/70 nm column (IZON, SP1). The qEV column was equilibrated by PBS solution filtered using a sterile 0.22 µm filter. Fresh serum samples were loaded onto the loading frit of the qEV column. The first 3 mL of default buffer was discarded and the following 1.5 mL buffer containing EVs was collected. All the patient information were shown in Table S7.

### 2.25 Software

Image J version 1.52v and Image‐Pro Plus version 6.0 were used for imaging analysis. Data were graphed and statistically tested using GraphPad Prism 9 and R Studio 1.1.463. Bio‐Rad CFX Manager version 3.1 was used to collect real‐time PCR data. NF Profession 2.0 was used for nanoFCM. PyMOL version 2.5.2 was used to display structures and docking results. GSEA version 4.0.2 was used for gene set enrichment analysis of RNA‐seq data. Metascape (https://metascape.org/) was used for Gene ontology (GO) analysis of secreted protein mass spectrum. TargetScan 7.2 (https://www.targetscan.org/vert_72/) was used to predict miRNAs targeting AGMAT. RNA binding proteins (RBPs) of miR‐33a were analysed using RBPDB (http://rbpdb.ccbr.utoronto.ca/). The structure of human ACO1 protein was obtained from AlphaFold Protein Structure Database (https://alphafold.com/).

### 2.26 Statistics and Reproducibility

All quantitative data are presented as mean ± standard deviation. Two‐tailed Student's *t*‐tests were used for comparison of means of data between two groups. For multiple independent groups, one‐way or two‐way ANOVA with post hoc Tukey tests were used. Values of *p* < 0.05 were considered significant. Sample size was generally chosen based on preliminary data indicating the variance within each group and the differences between groups. Pearson correlation analysis was employed for analysing the strength and direction of the linear relationship between two variables and represented as *R* ^2^ value (square of the Pearson correlation coefficient *R*). All samples that have received the proper procedures with confidence were included for analyses. Animals were randomized before treatments. Western blots were repeated independently three times with similar results, and representative images are shown.

## 3 Results

### 3.1 Polyamine Spatially Distributes in TME

**FIGURE 1:**
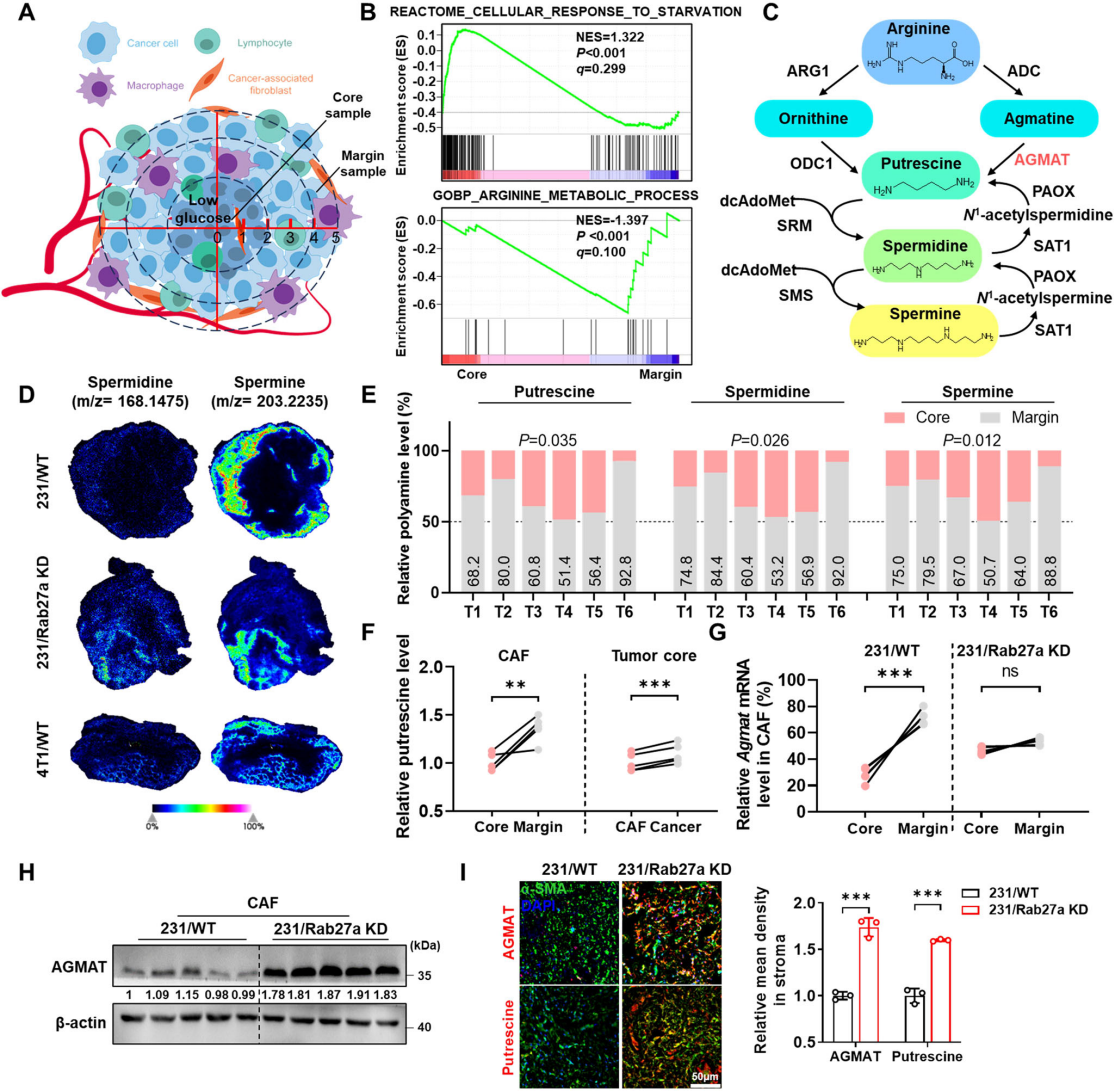
Polyamine metabolism spatially alters in tumour microenvironment. (A) Schematic diagram of the tumour spatial structure. Color deepening area, tumour core region. (B) GSEA analysis identified the cellular response to starvation signature in phenotype tumour core and the arginine metabolic process signature in phenotype tumour margin. (C) Schematic diagram depicting polyamine metabolic pathway. ARG1, arginase 1; ADC, arginine decarboxylase; ODC1, ornithine decarboxylase 1; AGMAT, agmatinase; SRM, spermidine synthase; SMS, spermine synthase; SAT1, diamine acetyltransferase 1; PAOX, polyamine oxidase. (D) Representative mass spectrometry imaging for spermidine and spermine abundance in 231 and 4T1 wild‐type tumours and 231/Rab27a KD tumour. (E) 4T1 xenograft tumours were harvested and separated into core and margin samples. Metabolites were extracted from each sample and polyamine concentration was measured by targeted LC‐MS/MS. Data are presented as mean ± s.d., *n* = 6 biological replicates, paired two‐tailed Student's *t*‐test. (F) Relative putrescine levels within tumour core and margin regional mouse cancer‐associated fibroblasts (mCAFs) derived from 4T1/WT tumours (left) and mCAFs or tumour cells from tumour core region (right). Putrescine concentrations were measured by ELISA kit. Data are presented as mean ± s.d., *n* = 5 biological replicates, paired two‐tailed Student's *t*‐test. (G) RNA levels of *Agmat* in mCAFs derived from tumour core and marginal regions from 231/WT and 231/Rab27a KD xenograft tumours were detected by RT‐qPCR. Data are presented as mean ± s.d., *n* = 5 biological replicates, paired two‐tailed Student's *t*‐test. (H) 231/WT and 231/Rab27a KD tumour tissues were harvested from NSG mice. Tissues from the core regions were separated to isolate mCAFs followed by western blots analysis. (I) Representative mIHC images showing AGMAT and putrescine staining of NSG 231/WT and 231/Rab27a KD xenograft tumour in core region mCAFs (α‐SMA). α‐SMA (green), AGMAT/Putrescine (red) and DAPI (blue). Quantification of AGMAT/Putrescine and α‐SMA colocalization presented as mean ± s.d., *n* = 3 mice, unpaired two‐tailed Student's *t*‐test.

Poor nutrient distribution in the tumour core (TC) region due to lack of blood vessel penetration is one of metabolic heterogeneity in TME (Figure 1A). To better understand the genetic regulation of metabolic heterogeneity, we performed RNA‐seq for both tumour core and margin (TM) from murine primary breast tumours. We found an upregulated cellular response to starvation pathway but suppressed arginine metabolic process which ranked top 2 pathways based on gene set enrichment analysis (GSEA) in TC when compared to TM (Figures 1B and S1A), suggesting arginine metabolism might contribute to starvation adaption in relative core region. Intriguingly, AGMAT, responsible for the arginine conversion into putrescine, was identified significantly downregulated in the core region than relative periphery tissue (Figures 1C and S1B).

We next examined the polyamine levels for tumours from mice xenografted with human (231/WT and 231/Rab27a KD with impaired exosomes secretion) and murine triple‐negative breast cancer cells (4T1/WT) by employing mass spectrometry imaging (MSI), with haematoxylin‐eosin (H&E) staining showing the morphology of those tumours (Figure S1C). Spermine, spermidine, *N* ^1^‐Acetylspermine and *N* ^1^‐Acetylspermidine abundances were significantly decreased in TC than TM in both 231/WT and 4T1/WT tumours rather than 231/Rab27a KD tumours (Figures 1D and S1D). However, putrescine failed to be accurately captured due to the high noise background. Therefore, we further employed targeted LC‐MS/MS to validate the concentration of polyamines and found they were remarkably decreased in TC when compared to TM (Figure 1E). Cancer‐associated fibroblasts (CAFs), the predominant stromal component in the TME, drive tumour progression through the crosstalk with cancer cells (Sahai et al. 2020; Kalluri 2016). We found that the levels of putrescine especially in CAFs residing TC area were significantly lower than those in TM area and TC tumour cells (Figure 1F). Next, we examined the putrescine levels in the serum samples derived from breast cancer patients and tumour bearing mice using ELISA kit. However, no significant differences were observed between patients and healthy donors, nor between tumour‐bearing and tumour‐free mice, indicating that putrescine produced in tumours would not release into the circulation (Figure S1E). In agreement with previous reports, both 4T1/Rab27a KO and 231/Rab27a KD cells with none or very low amount of Rab27a protein expression, displayed severely impaired capability for EV secretion (Figure S1F,G). For further validating the function of EVs, we treated MDA‐MB‐231 cells with GW4869 (Essandoh et al. 2015). We found that EVs derived from GW4869‐treated cells restored the AGMAT levels in human CAFs (Figure S1H). Consistently, RNA expression of AGMAT was suppressed in TC than TM area from 231/WT CAFs. However, this differential expression detected in 231/WT was totally abolished between TC and TM region from 231/Rab27a KD CAFs (Figure 1G). The AGMAT level was much lower in TC CAFs from 231/WT tumours than those from 231/Rab27a KD tumours (Figure 1H). Further multiplexed immunohistochemistry (mIHC) assay confirmed both AGMAT and putrescine levels were suppressed in the TC stroma of 231/WT compared to 231/Rab27a KD tumours (Figure 1I). However, there was undetectable change for putrescine concentration between TC and TM from either 231/Rab27a KD or 4T1/Rab27a KO mice (Figure S1I). All those observations suggested spatially variation of polyamine metabolism was partially mediated by tumour‐derived EVs.

### 3.2 Cancer Cell‐Secreted miR‐33a Suppresses Polyamine Metabolism by Targeting AGMAT in CAFs

**FIGURE 2:**
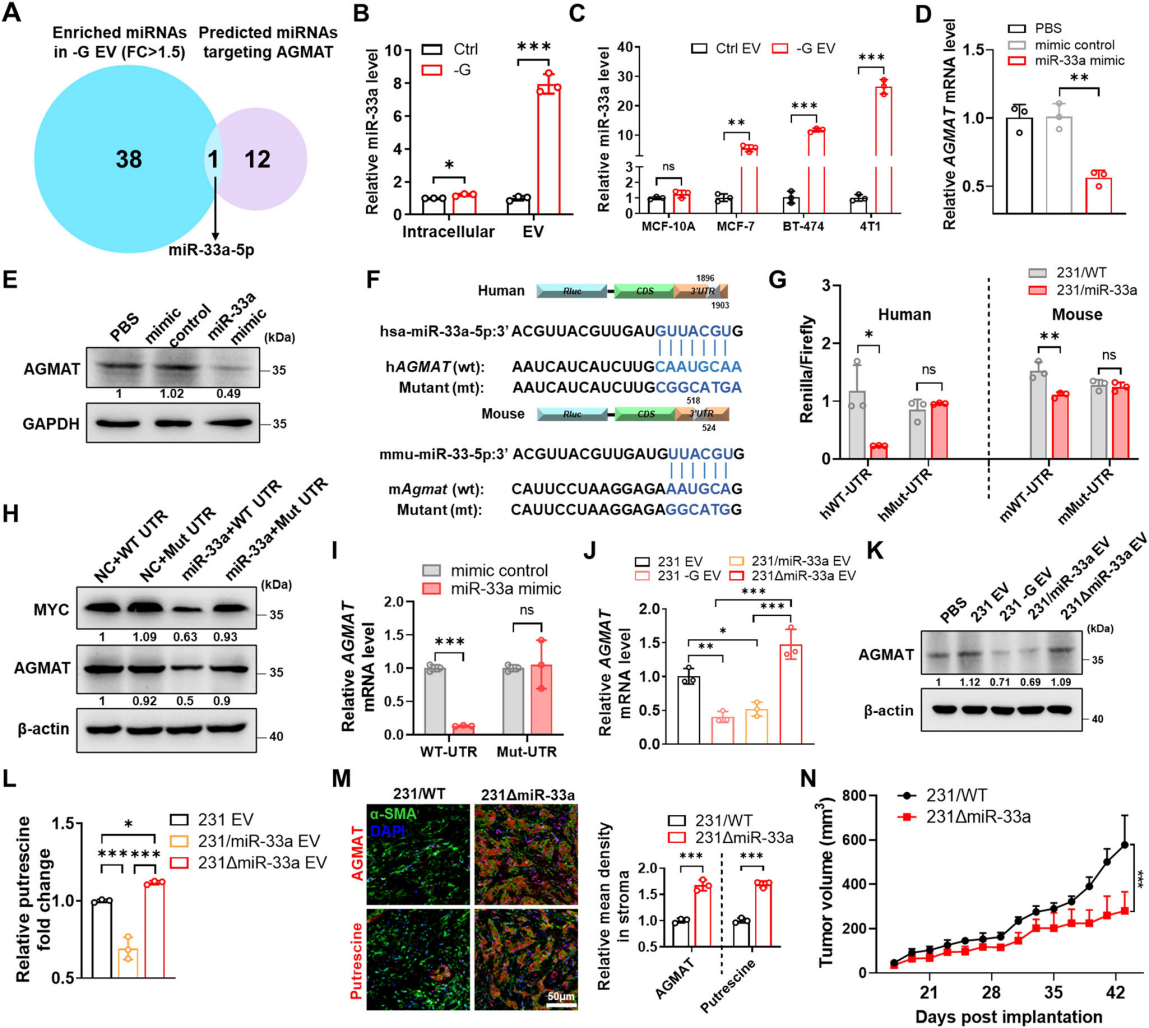
Cancer cell‐secreted miR‐33a inhibits putrescine production by targeting AGMAT in CAFs. (A) Venn diagram of upregulated exosomal miRNA from 231 ‐G EV versus 231 EV by miRNA‐seq (fold change > 1.5) and predicted miRNAs targeting AGMAT (TargetScan). ‐G indicates glucose free. (B) Relative intracellular or exosomal miR‐33a levels from glucose starvation cultured MDA‐MB‐231 compared to those for complete medium cultured cells were determined by RT‐qPCR. Data are presented as mean ± s.d., *n* = 3 biological replicates, unpaired two‐tailed Student's *t*‐test. (C) RT‐qPCR‐determined levels of exosomal miR‐33a from different breast cancer cell lines under glucose starvation treatment compared with complete medium cultured. Data are presented as mean ± s.d., *n* = 3 biological replicates, unpaired two‐tailed Student's *t*‐test. (D) RNA levels of AGMAT in human cancer‐associated fibroblasts (hCAFs) transfected with miR‐33a or control mimic. Data are presented as mean ± s.d., *n* = 3 biological replicates, unpaired two‐tailed Student's *t*‐test. (E) Protein levels of AGMAT in hCAFs transfected with miR‐33a or control mimic. (F) Predicted miR‐33a targeting human and mouse AGMAT 3'UTR binding sites. The corresponding sequences in WT and mutated reporters are shown. (G) Responsiveness of the WT and mutant AGMAT/Agmat 3'UTR reporters to miR‐33a in stable transfected cell lines. Data are presented as mean ± s.d., *n* = 3 biological replicates, unpaired two‐tailed Student's *t*‐test. (H) AGMAT levels in 231 transfected with AGMAT‐Myc‐WT 3′UTR or AGMAT‐Myc‐Mut 3′UTR were detected followed by miR‐33a mimic or negative control mimic treatment. (I) RNA levels of *AGMAT* in 231 transfected with AGMAT‐Myc‐WT 3′UTR or AGMAT‐Myc‐Mut 3′UTR were detected followed by miR‐33a mimic or negative control mimic treatment. Data are presented as mean ± s.d., *n* = 3 biological replicates, unpaired two‐tailed Student's *t*‐test. (J) Human CAFs were treated with EVs for 24 h to detect *AGMAT* level. Data are presented as mean ± s.d., *n*  =  3 biological replicates, one‐way analysis of variance (ANOVA), Tukey's multiple comparisons test. (K) AGMAT protein level of EVs treated hCAFs was detected by western blots. (L) The intracellular putrescine levels of EVs treated mCAFs measured by LC‐MS/MS. (succinate‐1,4‐^13^C_2_ as internal standard). Data are presented as mean ± s.d., *n*  =  3 biological replicates, one‐way ANOVA, Tukey's multiple comparisons test. (M) Representative mIHC images showing AGMAT and putrescine staining of NSG 231/WT tumour and 231ΔmiR‐33a tumour in core region mCAFs (α‐SMA). α‐SMA (green), AGMAT/Putrescine (red) and DAPI (blue). Quantification of AGMAT/Putrescine and α‐SMA colocalization presented as mean ± s.d., *n* = 3 mice, unpaired two‐tailed Student's *t*‐test. (N) Tumour volume of 231/WT and 231ΔmiR‐33a xenograft in NSG mice. Data are presented as mean ± s.d., *n* = 7 biological replicates, two‐way ANOVA, Sidak's multiple comparisons test.

Considering the amount and profiles of EVs alter under stress condition (Lisi et al. 2023; Virga et al. 2021; Borras et al. 2020), we performed the proteomic analysis (TMT‐MS) for cancer derived EVs to determine the underlying mechanism (see Table S3). We compared EV proteins enriched in both MDA‐MB‐231 cells under glucose starvation (‐G EV) versus normal glucose (231 EVs), and tumour core region (TC) versus tumour margin region (TM). However, none of the overlapped protein candidates had high association with arginine/polyamine metabolism. We next performed small RNA sequencing for EV‐derived from glucose‐free medium treated cancer cells (231 ‐G EV) versus normal glucose (3 g/L) medium treated cancer cells (231 EV) (Table S4) and identified that miR‐33a‐5p was the only miRNA both enriched in 231 ‐G EV and database predicted candidates potentially targeting AGMAT (Figure 2A). Notably, glucose starvation significantly upregulated the miR‐33a levels in both MDA‐MB‐231 cells and 231 EV, even though the number of particles was also elevated under glucose starvation, suggesting the responsive miR‐33a secretion of cancer cells partially due to the glucose starvation (Figures 2B and S2A,B). Likewise, we found several different breast cancer cell lines including MCF‐7, BT‐474 and 4T1 cells rather than normal mammary epithelial MCF‐10A cells secreted more miR‐33a into EVs under glucose starvation than those cells under normal glucose treatment (Figure 2C). Furthermore, significantly elevated miR‐33a levels were found in either tissues or EVs derived from tumour core compared to the tumour margin (Figure S2C,D).

Transfection of CAFs with miR‐33a mimic significantly suppressed AGMAT expression when compared to mimic control (Figure 2D,E). Further interrogation of sequences in the 3′UTR of human and mouse AGMAT genes identified a single miR‐33a binding site in *h*AGMAT and *m*Agmat, respectively (Figure 2F). Luciferase reporter assays comparing miR‐33a responsiveness of wild‐type and miR‐33a‐site‐mutated constructs confirmed direct targeting of AGMAT by miR‐33a (Figure 2G). Along with this finding, miR‐33a mimic treatment or overexpression significantly suppressed the total AGMAT abundance in MDA‐MB‐231 cells transfected with exogenous AGMAT gene with wild‐type 3’UTR rather than AGMAT with mutated 3’UTR (Figure 2H,I). In addition, we tracked the EVs‐derived from tumour cells with overexpression of a Lck‐GFP plasmid, with the secreted EVs carrying green fluorescence (Yan et al. 2022). The results showed CAFs rather than macrophages or granulocytes exhibited capacity to absorb tumour derived Lck‐GFP EVs (Figure S2E).

To further confirm the effect of breast cancer‐secreted exosomal‐miR‐33a in the regulation of polyamine metabolism, we knocked out the *MIR33A* gene in MDA‐MB‐231 cells by employing the CRISPR‐Cas9 technique (Figure S2F). Both the intracellular and EV miR‐33a levels were significantly decreased with *MIR33A* knockout in MDA‐MB‐231 cells (Figure S2G). Of note, as the gene *MIR33A* localized within *SREBF2*, miR‐33a deletion had no effect on the expression of *SREBF2* (Figure S2H). CAFs incubation with miR‐33a enriched EV displayed reduced AGMAT level, while 231∆miR‐33a EV treatment restored AGMAT abundance in CAFs (Figure 2J,K). Similar pattern was detected in putrescine level in CAFs upon EV treatment (Figure 2L). In comparison, 231∆miR‐33a EV displayed similar particle size when compared with 231 EV, 231 ‐G EV or 231/miR‐33a EV, and all EVs displayed classical exosome marker expressions (CD9, CD63, TSG101 and Alix), with negative expression of Golgi apparatus protein GM130 after gradient centrifugation (Figures S2I–K). Additionally, mIHC also supported AGMAT or putrescine evenly downregulated in CAFs from 231/WT than 231∆miR‐33a tumour (Figure 2M). Of note, miR‐33a knockout significantly suppressed the tumour growth rate in MDA‐MB‐231 cell‐xenografted mouse models (Figure 2N). All those results supported the pro‐tumour contribution of exosomal‐miR‐33a in primary tumours.

### 3.3 Putrescine Enhances H3K4 Tri‐Methylation by Inhibiting KDM5C Expression

**FIGURE 3:**
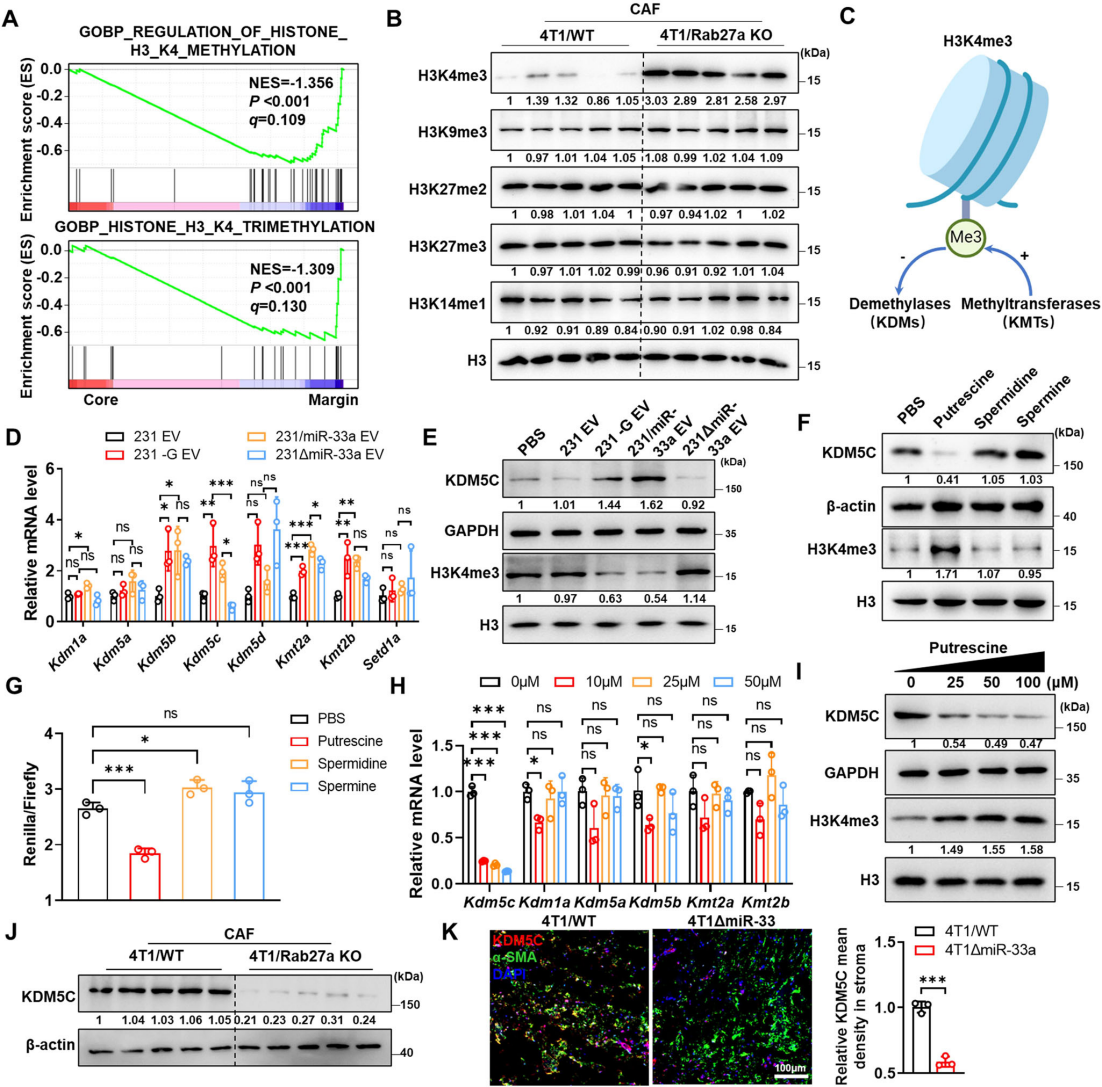
Putrescine enhances H3K4 tri‐methylation by inhibiting KDM5C expression. (A) GSEA analysis identified the regulation of histone H3K4 methylation signature and histone H3K4 trimethylation upregulated in phenotype tumour margin. (B) Mouse CAFs from core region of 4T1/WT and 4T1/Rab27a KO xenograft tumours were used for histone extraction. Histone methylation levels were assessed by western blots. (C) The schematic diagram showing the histone lysine methyltransferase and demethylases. (D) RNA levels of histone methyltransferase and demethylase of mCAFs treated with EVs. Data are presented as mean ± s.d., *n*  =  3 biological replicates, one‐way ANOVA, Tukey's multiple comparisons test. (E) KDM5C and H3K4me3 protein levels of mCAFs with EVs treatment were examined by western blots. (F) Western blots showing the expression levels of KDM5C and H3K4me3 in mCAFs treated with PBS or polyamine (25 µM). Arginine free medium was supplied. (G) Responsiveness of the human KDM5C 5'UTR reporters to polyamine (25 µM). Data are presented as mean ± s.d., *n*  =  3 biological replicates, one‐way ANOVA, Dunnett's multiple comparisons test. (H) RNA levels of histone methyltransferases and demethylases of mCAFs treated with gradient concentration of putrescine. Arginine free medium was supplied. Data are presented as mean ± s.d., *n*  =  3 biological replicates, one‐way ANOVA, Dunnett's multiple comparisons test. (I) Western blots showing the expression levels of KDM5C and H3K4me3 in mCAFs treated with gradient concentration of putrescine. Arginine free medium was supplied. (J) KDM5C levels in mCAFs from the core regions of 4T1/WT or 4T1/Rab27a KO mice were assessed by western blots. (K) Representative mIHC images showing KDM5C staining of 4T1/WT tumour and 4T1ΔmiR‐33 tumours in core region mCAFs (α‐SMA). α‐SMA (green), KDM5C (red) and DAPI (blue). Quantification of KDM5C and α‐SMA colocalization presented as mean ± s.d., *n* = 3 mice, unpaired two‐tailed Student's t‐test.

To further explore the biological effect of reduced polyamine in TC, we performed RNA‐sequencing and GSEA analysis, and identified H3K4 trimethylation was significantly suppressed in TC than TM regions (Figure 3A). Herein, we examined several methylation markers on the H3 protein in CAF cells and identified only H3K4me3 downregulated in CAFs from 4T1/WT than 4T1/Rab27a KO tumour (Figure 3B). Considering polyamines have been reported to regulate the amount of histone lysine demethylases LSD1 (KDM1A) (Tamari et al. 2018). We examined the RNA level of *Kdm1a* and the other histone methyltransferase or demethylases and found *Kdm5c*, rather than other methyltransferase or demethylases was induced by miR‐33a (Figure 3C–E).

To further identify which specific polyamine was involved in the regulation of KDM5C, we separately treated CAFs with putrescine, spermine and spermidine. Intriguingly, we found only putrescine significantly suppressed KDM5C expression by binding its 5′UTR, thereby inducing H3K4 tri‐methylation (Figure 3F,G). Our docking models also demonstrate putrescine rather than spermine or spermidine binds the 5′UTR of KDM5C and thereby suppresses its translation (Figure S3A). Further luciferase report assay validated that the sequence ‘GAAGGCU’ in the 5′UTR of KDM5C mediated its interaction with putrescine (Figure S3B,C). Furthermore, microscale thermophoresis (MST) assay showed putrescine binding to wild‐type rather than mutant KDM5C 5′UTR (Figure S3D,E). Additionally, we found putrescine regulated both the expression of KDM5C and downstream H3K4me3 in CAFs in a dose‐dependent manner (Figure 3H,I). The expression of *Kdm5c* was downregulated while the level of H3K4me3 increased upon putrescine treatment in NIH3T3 cells (Figure S3F,G). The regulatory effect of putrescine was abolished due to the overexpression of KDM5C (Figure S3H). KDM5C was also upregulated due to the high miR‐33a level in CAFs from 4T1/WT rather than 4T1/Rab27a KO tumours (Figure 3J). Exactly, there were more KDM5C expression detected in CAFs from 4T1/WT rather than 4T1∆miR‐33a tumour (Figure 3K). Considering polyamines have been reported to upregulate downstream gene expression by promoting eIF5A hypusination (Li et al. 2022; Zhang et al. 2019; Puleston et al. 2019), we employed the DHPS inhibitor N1‐guanyl‐1,7‐diaminoheptane (GC7) to inhibit eIF5A hypusination and confirmed eIF5A hypusination had no effect on KDM5C level (Figure S3I). Likewise, we also employed eIF5A fibroblast conditional knockout mice (*eIF5A^F‐/−^*) and validated the expression of eIF5A had no effect on KDM5C and H3K4me3, further confirming the regulation of KDM5C by putrescine was in an EIF5A‐independent manner (Figure S3J). All the data showed miR‐33a remodelled the epigenetic profile by suppression of putrescine biogenesis in CAFs, highlighting the central role of KDM5C in the regulation of H3K4me3 by putrescine.

### 3.4 MiR‐33a Reprograms Epigenetic Profile to Induce Stress Granule Disassembling in Stroma

**FIGURE 4:**
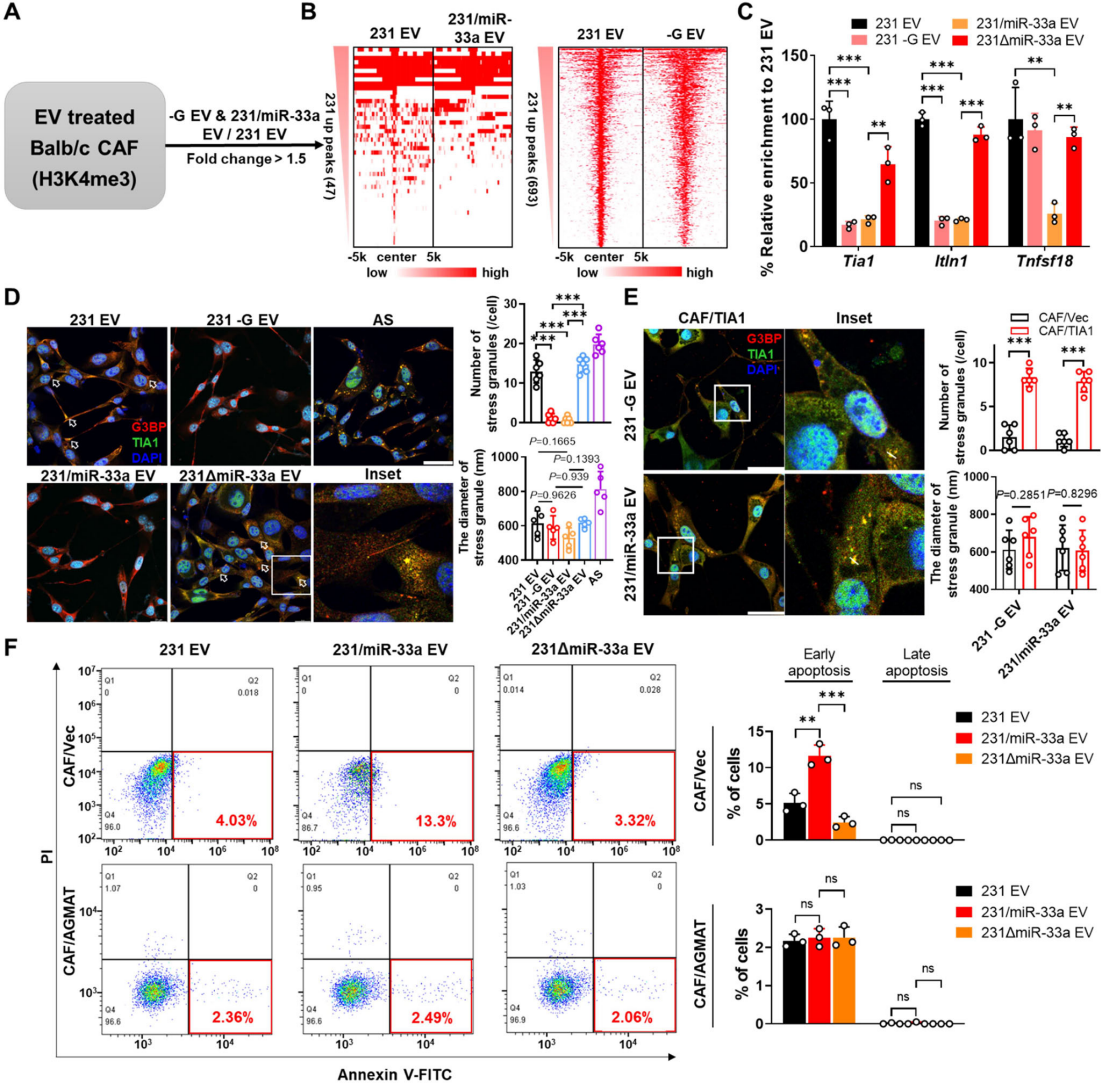
MiR‐33a reprograms epigenetic profile to induce stress granule disassembling in CAF. (A) Experimental workflow for studying the H3K4me3 landscapes of EV treated mCAFs. (B) Heatmap of ChIP‐seq experiments using antibody against H3K4me3 in EV treated mCAFs. (C) ChIP‐qPCR was used to detect the binding of H3K4me3 with putative elements near *Tia1*, *Itln1* and *Tnfsf18* in mCAFs after EV treatment. Relative enrichment score was presented as the ratio of corresponding group to 231 EV treatment. Data are presented as mean ± s.d., *n*  =  3 biological replicates, one‐way ANOVA, Tukey's multiple comparisons test. (D) Immunofluorescence of mCAFs treated with EVs under low glucose medium or exposed to sodium arsenite (AS, 400 µM; 1 h) and stained for the SG markers TIA1 and G3BP1. Scale bar, 50 µm. Quantification of the number and diameters of stress granules (SGs) were presented as mean ± s.d., *n*  =  6 different cells, one‐way ANOVA, Tukey's multiple comparisons test. (E) Immunofluorescence of EV treated mCAFs transfected with TIA1 or Vec under low glucose medium followed by staining for SG marker TIA1 and G3BP1. Scale bar, 50 µm. Quantification of the number and diameters of stress granules (SGs) were presented as mean ± s.d., *n*  =  6 different cells, unpaired two‐tailed Student's *t*‐test. (F) Representative scatter plots of PI versus Annexin V showed the apoptosis of EVs treated CAF/Vec or CAF/AGMAT by flow cytometry. Quantification of apoptosis and necrosis are presented as mean ± s.d., *n*  =  3 biological replicates, one‐way ANOVA, Tukey's multiple comparisons test.

To establish a comprehensive genome‐wide view of H3K4me3 educated by miR‐33a, we then performed H3K4me3 ChIP‐sequencing to analyse EV educated CAFs (Figure 4A). Compared to 231 EV educated CAFs, a cluster of 647 genes and 27 genes were repressed in 231 ‐G EV and 231/miR‐33a EV treated CAFs (Table S5), with 16 genes in common (Figure 4B). To test whether alterations in chromatin modifications specifically modulated gene expression, we also assessed H3K4me3 enrichment levels on*Tia1*, *Itln1* and *Tnfsf18* altered peak region by ChIP‐qPCR. The region levels were significantly decreased by 231/miR‐33a EV and 231‐G EV treatment along with H3K4me3 immunoprecipitation but not significantly immunoprecipitated with anti‐H3K27me3 antibody when compared with 231 EV educated CAFs (Figures 4C and S4A). TIA1, a critical component for eukaryotic stress response and stress granules (SGs) formation, was transcriptionally inhibited in 231/miR‐33a EV and ‐G EV treated CAFs compared to CAFs incubation with 231 EV or 231ΔmiR‐33a EV (Figure S4B). Furthermore, 231/miR‐33a EV and ‐G EV treatment significantly reduced the number of SGs in CAFs, which were marked by immunofluorescence staining for TIA1 and G3BP (Figure 4D). With restoration of TIA1 expression, CAFs recovered the ability to assemble discernible SGs either when incubation with 231 ‐G EV or 231/miR‐33a EV, further evidencing TIA‐1 as a key factor involved in the signalling of miR‐33a mediated SG formation (Figure 4E). Previous reports showed that TIA1 deficiency could lead to inhibition of cell proliferation (Sanchez‐Jimenez and Izquierdo 2013). We detected the apoptosis and necrosis of CAF/Vec cells or CAF/AGMAT cells under EVs treatment by Flow Cytometry. The results showed that 231 ‐G EV or 231/miR‐33a EV increased early apoptosis in CAFs and restored in CAF/AGMAT when compared to 231 EV (Figure 4F). Moreover, TIA1 overexpression significantly promoted CAFs proliferation (Figure S4C). We also observed MDA‐MB‐231 cells rather than 231ΔmiR‐33a cells gained higher proliferation capability when cocultured with CAFs rather than MDA‐MB‐231 cells in glucose free medium (Figure S4D,E). Additionally, TIA1 overexpression in CAFs strikingly decreased tumour growth in 4T1 and CAF/TIA1 co‐injected model (Figure S4F). To assess the biological role of tumour‐derived miR‐33a mediated epigenetic remodelling, KDM5C inhibitor (KDM5‐C70) was applied to treat CAFs incubated with EVs. We found TIA1 was significantly upregulated than with untreated controls (Figure S4G,H). Collectively, we identified miR‐33a dysregulated SGs formation in CAFs and inhibited its survival capability and further mediated cancer cell growth.

### 3.5 Nutrient Restoration Blocks the Effect of miR‐33a in Stress Granules Formation

**FIGURE 5:**
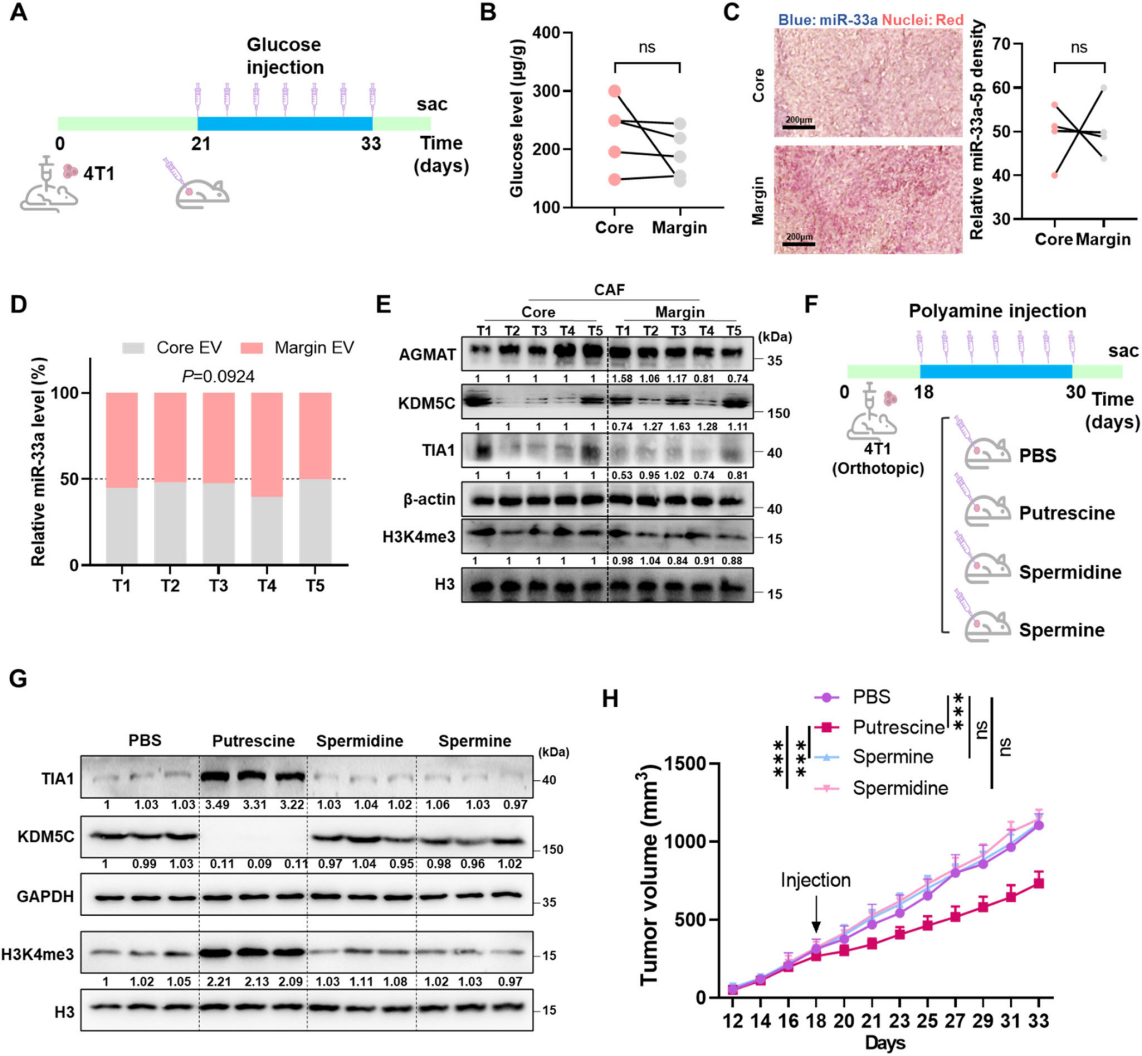
Nutrient supply blocks the effect of miR‐33a in tumour core. (A) The strategy of BALB/c 4T1 xenograft tumours with PBS or glucose injection into core region was shown. Glucose (1 g/L) was administered into tumour core every other day at 25 µL per tumour 3 weeks. (B) The glucose concentrations of core and margin regions of BALB/c 4T1 glucose injected tumours were detected. Data are presented as dots, *n* = 5 mice, paired two‐tailed Student's *t*‐test. (C) Representative in situ hybridization images showing the miR‐33a level in the core and margin regions of glucose‐injected 4T1 tumour. Scale bar, 200 µm. Quantification of miR‐33a level presented as mean ±s.d., *n* = 5 mice, paired two‐tailed Student's *t*‐test. (D) RT‐qPCR‐determined miR‐33a levels of EVs from 4T1 glucose injection tumour in core and margin regions. Data are presented as mean ± s.d., *n* = 5 biological replicates, paired two‐tailed Student's *t*‐test. (E) Samples from core or margin mCAFs of the 4T1 glucose injected tumours as indicated were harvested and gene expression levels were detected by western blots. (F) The injection strategy of BALB/c 4T1 xenograft tumours with polyamine injection into core region was shown. Putrescine, spermidine and spermine (25 µM) was administered into tumour core every other day at 25 µL per tumour. (G) Core mCAFs of the 4T1 PBS or polyamine injected tumours as indicated were harvested and protein levels were detected by western blots. (H) Tumour volume of 4T1 PBS or polyamine injected tumours. Data are presented as mean ± s.d., *n* = 5 mice, two‐way ANOVA, Sidak's multiple comparisons test.

Considering glucose starvation was the key source to trigger miR‐33a secretion, we injected glucose into TC of 4T1/WT tumour to determine whether glucose supplementary in the tumour core region could block the effect of miR‐33a/AGMAT axis in vivo (Figure 5A). We observed no significant differences for either glucose level or miR‐33a between core and margin regions in glucose‐injected tumours (Figure 5B,C). However, the core glucose levels were significantly reduced in PBS‐injected tumours (Figure S5A). We also measured the secretion of miR‐33a abundance in EVs and found that there were no significant differences of secreted miR‐33a between TC and TM EVs (Figure 5D). Consistently, there was no significantly differential expression of AGMAT, KDM5C, TIA1 or H3K4me3 between CAFs from TC and TM (Figure 5E). Furthermore, IHC data demonstrated that more homogeneous expression of AGMAT, putrescine and H3K4me3 levels in the core and margin stroma of the glucose‐injected tumours (Figure S5B). Likewise, to confirm the central role of polyamine in glucose starvation induced tumour stroma remodelling, we injected PBS, putrescine, spermine and spermidine into the tumour core region of 4T1 tumour (Figure 5F), and we found only putrescine rather than spermine or spermidine injection blocked the differential expression pattern of KDM5C and restored TIA1 and H3K4me3 in TC (Figures 5G and S5C). Only injecting putrescine into the tumour core region could decrease tumour growth when compared to spermine or spermidine injection (Figure 5H). Additionally, 4T1 and wild‐type or AGMAT overexpressed CAFs co‐injected model was performed to mimic the putrescine rescue model (Figure S5D). CAFs with AGMAT overexpression notably inhibited 4T1 tumour growth, and strikingly decreased weight of tumours (Figure S5E,F). Moreover, AGMAT overexpression in CAFs restored the AGMAT and TIA1 levels in the tumour core regions, as well as the H3K4me3 levels and blocked the KDM5C levels (Figure S5G–I). Those results further confirmed putrescine as the key modulator for miR‐33a‐5p mediated epigenetic remodelling.

### 3.6 RNA Binding Protein ACO1 Assists miR‐33a Secretion Under Low Iron Levels

**FIGURE 6:**
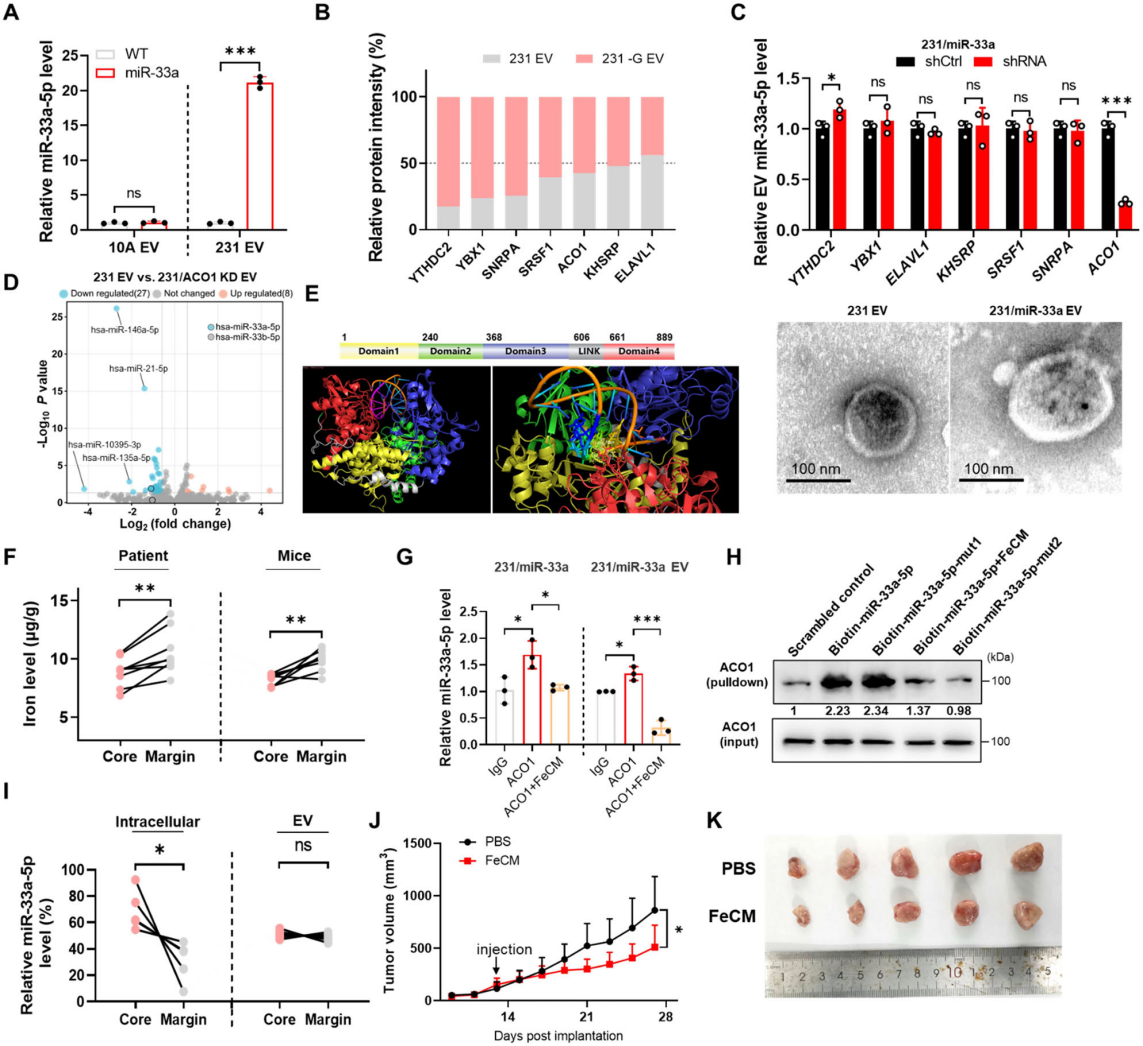
RNA binding protein ACO1 assists miR‐33a secretion under low iron level. (A) MiR‐33a levels were detected in EVs derived from MCF‐10A (left) or 231 (right) stably transfected with Lenti‐miR‐33a by RT‐qPCR. Data are presented as mean ± s.d., *n* = 3 biological replicates, unpaired two‐tailed Student's *t*‐test. (B) Diagram shows potential RNA binding protein levels in 231 control or ‐G EV detected by EVs Mass Spectrum. (C) MiR‐33a levels in EVs derived from 231/miR‐33a or putative RNA binding protein knockdown cells were measured by RT‐qPCR (Top). Data are presented as mean ± s.d., *n* = 3 biological replicates, unpaired two‐tailed Student's *t*‐test. Representative images of immuno‐EM analysis of small EVs purified from 231/WT or 231/miR‐33a and visualized with 10‐nm gold‐gold particles (Bottom). Scale bar, 100 nm. (D) Volcano plot of expression differences between 231 EVs and 231 ACO1 knockdown EVs as determined via miRNA‐seq. Pink‐colored points and cerulean blue‐colored points represent gene expression fold change > 1.5 and < −1.5, respectively (*p* values < 0.05). (E) Schematic diagram showed the sequence of miR‐33a and the structure of ACO1 binding miR‐33a. Color band was 3D structure of ACO1 while the orange chain showed the structure of *hsa*‐miR‐33a‐5p, red band represented the domain 4 of ACO1, docking result between *hsa*‐miR‐33a and ACO1. The purple band was the binding sequence CAUUG. The visualization was done by PyMOL 2.5.2. The structure of human ACO1 protein was obtained from AlphaFold Protein Structure Database based on PDB 2B3X. (F) Iron levels within tumour core and margin regions derived from patients (left) and mice (right). Data are presented as mean ± s.d., *n* = 8 biological replicates, paired two‐tailed Student's *t*‐test. (G) RIP assays with anti‐ACO1 antibody (with IgG as negative control) were performed in the cell (left) and EV (right) lysates from miR‐33a overexpressed in MDA‐MB‐231 cell lines. miR‐33a levels in immunoprecipitated samples were detected by RT‐qPCR and normalized to the corresponding input samples. Data are presented as mean ± s.d., *n* = 3 biological replicates, unpaired two‐tailed Student's *t*‐test. FeCM, ferric carboxymaltose. (H) Western blots analysis of ACO1 expression in exosome lysates subjected to miRNA pulldown with biotinylated miR‐33a or biotinylated miR‐33a mutant probes. MiR‐33a‐5p: GUGCAUUGUAGUUGCAUUGCA, miR‐33a‐5p mut1: GUCGUAACUAGUUGCAUUGCA, miR‐33a‐5p mut2: GUGCAUUGUAGUUGGUAACGA. (I) Relative intracellular (left) or exosomal (right) miR‐33a‐5p levels from core and margin regions of 4T1 FeCM injected tumours were detected by RT–qPCR. Data are presented as mean ± s.d., *n* = 5 biological replicates, paired two‐tailed Student's *t*‐test. (J) Tumour volume of 4T1/WT tumours with PBS or ferric carboxymaltose (FeCM) injection (0.3 mg) into tumour core region. Data are presented as mean ± s.d., *n* = 5 mice, two‐way ANOVA, Sidak's multiple comparisons test. (K) Representative tumour images in each group were shown.

To further seek the mechanism underlying the secretion of miR‐33a into EVs under glucose starvation, we found there was significant enrichment of miR‐33a encapsulated in 231/miR‐33a EVs rather than in 10A/miR‐33a EVs, when compared to their respective control cells (Figure 6A). However, the intracellular abundance of miR‐33a was much higher in MCF‐10A cells than MDA‐MB‐231 cells (Figure S6A), which suggests there might be some essential RBPs uniquely enriched in MDA‐MB‐231 cells rather than MCF‐10A cells to assist the secretion of miR‐33a. To further determine which RBP specifically bound miR‐33a for downstream packaging into EVs, we combined both predicted RBPs with miR‐33a from ‘RBPDB database’ and proteomic data between 231 EV and 231 ‐G EV (Table S6). Thereby, we recognized ACO1, a TCA enzyme, together with KHSRP, SNRPA and SRSF1 overlappingly enriched in 231 ‐G EV, serving as higher potential RBP candidates (Figure 6B). To further confirm which RBP was involved in miR‐33a secretion, we knocked down each of the four potential targets along with YBX1, YTHDF2 and ELAVL1 (known RBPs involved in miRNA secretion), remaining ∼half of their intracellular abundance in MDA‐MB‐231/miR‐33a cells (Figure S6B). We found only 231/miR‐33a/ACO1 KD cells secreted reduced amount of miR‐33a, while more ACO1 proteins were detected in 231/miR‐33a EV than 231 EV imaged by immunogold electron microscopy (Figure 6C). Conversely, miR‐33a secretion was blocked in 231/ACO1 KD cells compared to 231 cells based on small RNA‐sequencing data (Figure 6D). Besides, immunofluorescent staining clearly demonstrated the flow of intracellular ACO1 from mitochondria to multivesicular bodies (MVBs) under glucose starvation between 9 and 16 h (Figure S6C,D). ACO1 was also decreased in the mitochondria of glucose starved MDA‐MB‐231 cells (Figure S6E). Nevertheless, we used the mitophagy inhibitor Mitochondrial division inhibitor 1 (Mdivi‐1) and found the loss of TOM20 rather than ACO1 could be due to increased mitophagy (Figure S6F). In addition, the secretion of miR‐33a was increased by deleting the mitochondrial targeting sequences (MTS) of ACO1 when compared to wild‐type ACO1 (Figure S6G). Molecular docking model showed there was potential binding between mature *hsa*‐miR‐33a‐5p and domain 4 of human ACO1, and the purple band was the binding sequence ‘CAUUG’ (Figure 6E).

Considering RBP capability of ACO1 was highly dependent on iron level (Dupuy et al. 2006; Liu et al. 2022). We examined the iron level in TME to investigate the regulatory mechanism how ACO1 got involved in the secretion of miR‐33a. Indeed, we detected lower iron levels in TC than TM region of both patient and mouse tumours (Figure 6F). To experimentally confirm whether ACO1 bound miR‐33a directly, we performed RNA‐immunoprecipitation assay, which showed ACO1 could pull down miR‐33a from both intracellular and EV fractions, but was blocked by ferric carboxymaltose (FeCM) (Figure 6G). Similarly, biotinylated wild‐type miR‐33a and mutant miR‐33a outside of ‘CAUUGC’ could pull down ACO1, and would be inhibited by FeCM. However, the mutant ‘CAUUGC’ miR‐33a could not pull down ACO1 (Figure 6H). In agreement with this finding, the binding between ACO1 and miR‐33a was also supported by electrophoretic mobility shift assay (EMSA) (Figure S6H). Furthermore, notable reduction of iron levels was detected in cancer cells, rather than in CAFs from wild‐type 4T1 tumours core regions. This phenomenon disappeared in 4T1/Rab27a KO tumours (Figure S6I,J). In contrast, high level of iron supplement by FeCM injected into tumour core could inhibit the RBP ability of ACO1. The intracellular miR‐33a levels in TC were significantly higher than TM, while there was no change in EVs (Figure 6I). Besides, FeCM injected into TC region remarkably suppressed the tumour growth rate and reduced the tumour volume when compared to PBS injection (Figure 6J,K). Simultaneously, there was downregulated of KDM5C and upregulated of H3K4me3 in TC upon FeCM treatment (Figure S6K). Collectively, those results suggested poor iron levels enhanced miR‐33a secretion and FeCM supplementary might be a strategy for treating cancer.

### 3.7 MiR‐33a/AGMAT Axis Widely Exists in Breast Cancer Patients

**FIGURE 7:**
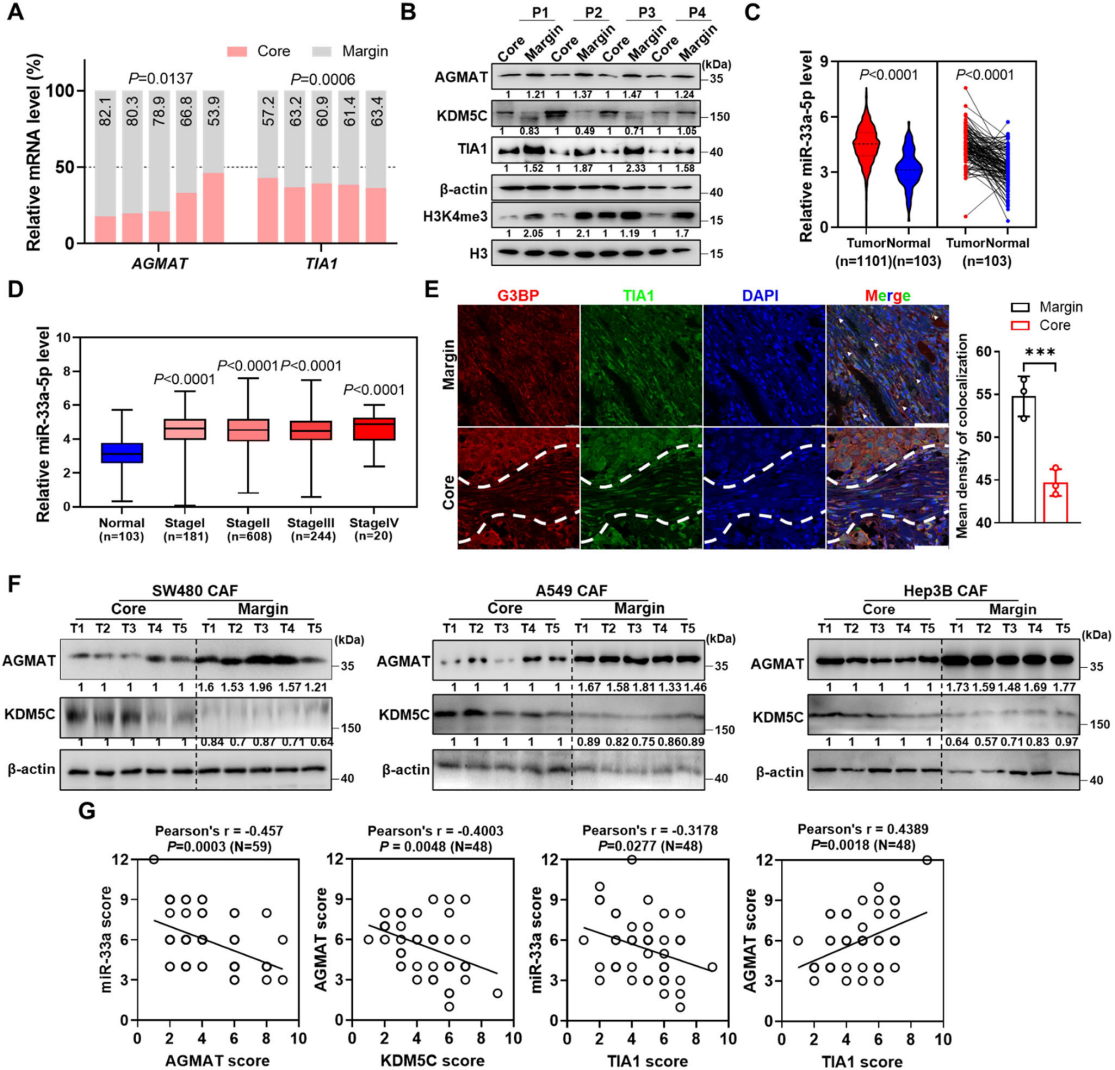
MiR‐33a/AGMAT axis widely exists in primary breast tumours and other cancer types. (A) RNA levels of indicated genes in core and margin hCAFs of patient primary tumours were detected. Data are presented as mean ± s.d., *n* = 5 patients, paired two‐tailed Student's *t*‐test. (B) Protein levels of indicated genes in core and margin hCAFs of patient primary tumours were detected. (C) Relative *hsa*‐miR‐33a‐5p levels in BRCA tumours and normal mammary tissues from TCGA databases. miR‐33a‐5p is upregulated in BRCAs. Statistical significance was assessed using two‐tailed Mann–Whitney test (left) and paired two‐tailed Student's *t*‐test (right). (D) Relative *hsa*‐miR‐33a‐5p levels of BRCA patients at different clinical stages by means of TNM stages from TCGA databases. Data are presented as mean ± s.d., one‐way ANOVA, Dunnett's multiple comparisons test. (E) Representative immunofluorescence images showing patient tissue SGs staining of core and margin primary tumour. Scale bar, 50 µm. Quantification of colocalization presented as mean ± s.d., *n* = 3 for core or margin of patient breast cancer samples, paired two‐tailed Student's *t*‐test. (F) Protein levels of indicated genes in Hep3B, SW480 and A549 tumour core and margin mCAFs were detected through western blots. (G) BC tissues analyzed for correlations among selected gene expression patterns by IHC/in situ hybridization‐determined scores. The Pearson's *r*, sample size (*N*; number of independent tissue samples) and *p* value (paired two‐tailed *t*‐test) are indicated.

To finally determine whether miR‐33a/AGMAT axis widely existed in patient samples, we examined some BC patients’ tumours and identified there were less AGMAT, H3K4me3 and TIA1 expression while more KDM5C expression in TC than in TM CAFs (Figure 7A,B). There was much higher miR‐33a level in TC than TM from BC patient tumour and their derived EVs (Figure S7A). However, the miR‐33a levels in EVs derived from patient serum samples were lower than healthy donors, indicating that exosomal‐miR‐33a solely plays a role in TME (Figure S7B). TCGA data and GEO dataset showed there was more miR‐33a in primary tumour than the normal tissue (Figures 7C and S7C). Increased miR‐33a was also detected in Stage I to IV BC specimen than adjacent normal tissue (Figure 7D). Interestingly, miR‐33a was generally overexpressed in multiple BC types, such as luminal A, luminal B and TNBC and partial Her‐2 positive patients (Figure S7D).

Consistent with TIA1 expression pattern, there were less SGs in the stroma from the tumour core region than their relative margins (Figure 7E). There were less AGMAT and more KDM5C expression detected in TC CAFs from various tumour types, such as colon (SW480), lung (A549) and liver (Hep3B) tumours (Figure 7F). Correlation analysis confirmed the negative correlation between the pair of miR‐33a/AGMAT, AGMAT /KDM5C and the pair of miR‐33a/TIA1, but positive correlation between the pair of AGMAT and TIA1 in the clinical BC samples (Figure 7G). Collectively, as shown by our graphical abstract, we found breast cancer cells secreted more miR‐33a‐5p with the assistance of RNA binding protein ACO1 under low iron and glucose starvation to further suppress the putrescine biogenesis by targeting AGMAT gene in CAFs. Downstream ChIP‐seq profiling revealed that TIA1 gene was tightly regulated by KDM5C/H3K4me3 axis, leading to dysregulated SGs formation in CAFs to favor cancer cells’ survival and stress adaption (Figure S7E).

## 4 Discussion

The heterogeneity for TME is considered as a major obstacle for cancer treatment (Vasan et al. 2019; Dagogo‐Jack and Shaw 2018; Marusyk et al. 2020). The heterogeneity within tumours is a complex, multifactorial phenomenon, integrating genetic, environmental nutrients and epigenetic inputs. Therefore, tumour cells display high epigenetic and phenotypic plasticity in TME (Karnoub et al. 2007; Dentro et al. 2021; Pe'er et al. 2021). Metabolism connects epigenetic profile either through the key enzymes or the metabolites of the flux. Here we show ACO1, a classical TCA enzyme, functions as RNA binding protein involved in miRNA secretion from cancer cells under glucose starvation. Simultaneously, our docking models demonstrate putrescine rather than spermine or spermidine binds the 5′UTR of KDM5C and thereby suppresses its translation (Figure S3A). Previous studies show glutamine deficiency in tumours promotes dedifferentiation through inhibition of histone demethylation due to decrease α‐KG level in TME (Pan et al. 2016). Unlike oncometabolites such as α‐KG, itaconate or 2‐hydroxyglutarate serving as crucial co‐substrate for the activity of demethylases (Hu et al. 2013; Dang et al. 2009; Chen et al. 2022), herein we demonstrate that putrescine oppositely functions as an anti‐tumour metabolite via decreasing the expression of KDM5C in a riboswitch like mode. It has been reported that polyamines either induced histone demethylase expression level by hypusination of eukaryotic translation initiation factor 5A as a substrate or activated mitochondrial trifunctional protein through binding to the α and β subunits of MTP with strong affinity to improve antitumour immunity (Tamari et al. 2018; Li et al. 2022; Pan et al. 2016; Al‐Habsi et al. 2022). However, there is lack of open reading frames encoding short polyproline tracks of KDM5C mRNA, which is required for efficient translation (Zhang et al. 2019; Gutierrez et al. 2013). Therefore, here we report a new mechanism that putrescine regulates H3K4me3 through post‐transcriptionally suppressing demethylase KDM5C's expression, which is independent of hypusination or directly binding the pocket of the enzyme. In addition, polyamine metabolism is associated with cancer‐driving pathways (Casero et al. 2018). PTEN‐PI3K‐mTORC1 regulates polyamine dynamics in human prostate cancer (Zabala‐Letona et al. 2017). WNT/MYC signalling pathway activates polyamine metabolism in cancer (Bi et al. 2024). Crosstalk occurs between polyamine metabolism and RAS pathways in various types of cancer (Novita Sari et al. 2021).

In our study, we identify EVs linking the polyamine metabolism and epigenetic remodelling in the heterogeneity of TME. As newly studied media, EVs also connect tumour and stroma cells in TME. Of note, another classical heterogeneity of TME refers to diverse cell types constitution beyond spatial epigenetic and metabolic distribution (Hinshaw and Shevde 2019). Here, we identified EVs derived from tumour core delivering more miR‐33a to neighbouring cancer associated fibroblast (CAF) cells. As immune cells are also important constituents of tumour stroma, such as the innate immune cells (macrophages, neutrophils, dendritic cells, innate lymphoid cells, myeloid‐derived suppressor cells and NK cells) as well as adaptive immune cells (T cells and B cells). Whether exosomal miR‐33a mediates the crosstalk between cancer cells and the proximal immune cells or involved in the tumour growth and metastasis still need to investigate. Collectively, our study reveals a novel mechanism that under glucose starvation, cancer cells utilize the education strategy by secreting EVs to gain advantage for nutrition competition with CAFs, opening for other potential regulation mechanisms between tumour cells and immune cells within TME for better survival and stress resistance.

## Author Contributions

**Wei Yan**: Conceptualization; funding acquisition; project administration; resources; supervision; validation; visualization; writing–original draft; Writing–review and editing. **Wei Yan**: conceived ideas. **Wei Yan, Min Wu, and Juanjuan Li**: contributed to project planning. **Wei Yan, Sheng Hu and Xu Li**: designed and performed most of the experiments. **Xiaohui Zhang**: assisted with cell line construction. **Meixin Li**: assisted with mouse experiments. **Haifeng Zhou**: assisted with nano flow cytometry analysis. **Juanjuan Li and Xiaoyu Fu**: provided clinical samples. **Qixin Hu and Min Wu**: performed ChIP‐seq experiments. **Chenyu Wang and Lianyun Li**: assisted with ChIP‐seq analysis. **Gang Deng, Wenda Huang and Xiaolu Zhao**: assisted with LC/MS/MS analyses. **Ao Hua and Zifu Li**: assisted with AFM data analysis. **Juan Wu**: assisted with histological analyses. **Mingzhou Chen**: assisted with stress granules analyses. **Wei Yan and Sheng Hu**: wrote the manuscript.

## Conflicts of Interest

The authors declare no conflicts of interest.

## Supporting information




**Supplementary Figures and Tables**: jev270153‐sup‐0001‐SuppMat.docx (replaced with an updated version with formated Tables and polished legend)

## Data Availability

The RNA‐seq data generated in this study have been deposited in the NCBI Gene Expression Omnibus (GEO) with accession code GSE222295, including GSE222291 (tumour core and margin) and GSE222293 (Extracellular vesicles from MDA‐MB‐231 and 231/ACO1 KD cells). Small RNA deep sequencing data deposited in the NCBI GEO with accession code GSE275167. ChIP‐Seq data generated in this study have been deposited in the NCBI Gene Expression Omnibus (GEO) with accession code GSE241351. Partial proteomic data were shown in Tables S3 and S6. The bulk RNA‐seq human BRCA data were derived from the TCGA Research Network. TGCA data were downloaded from the UCSC Xena platform (http://xena.ucsc.edu/). All other data supporting the findings of this study are available from the corresponding author on reasonable request.
