# Supplementary material for: Cancer Cell‐Secreted miR‐33a Reduces Stress Granule Formation by Targeting Polyamine Metabolism in Stroma to Promote Tumourigenesis
Source: J Extracell Vesicles. 2025 Sep 3;14(9):e70153. doi: 10.1002/jev2.70153 (PMC12408367; doi:10.1002/jev2.70153)
Supplement: Supplementary file 1 — Supplementary Figures and Tables: jev270153‐sup‐0001‐SuppMat.docx (replaced with an updated version with formated Tables and polished legend) [file JEV2-14-e70153-s001.docx]

**Supplementary Figures with legends**

**
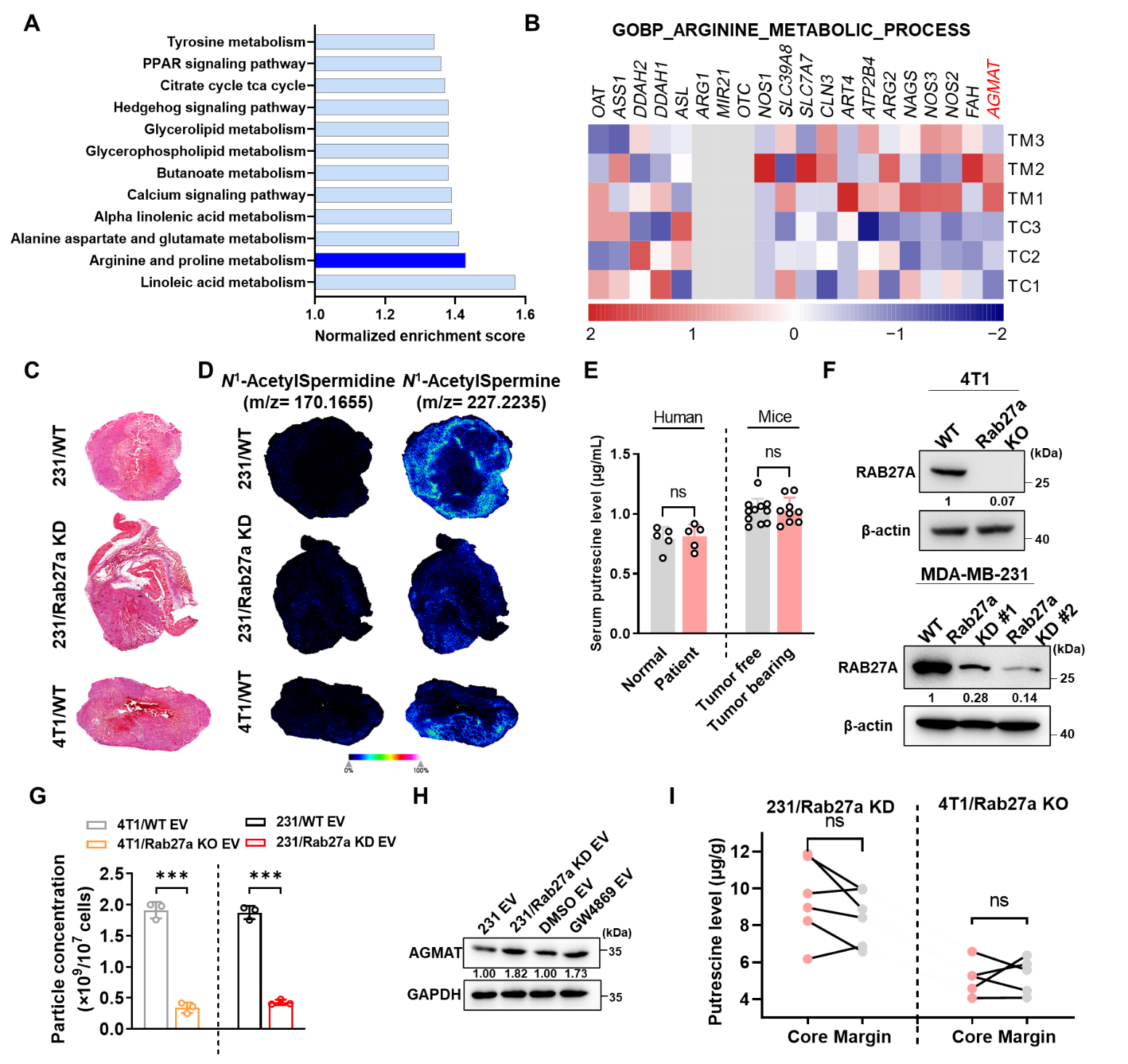
**

**Figure 1: Polyamine metabolism spatially alters in tumor microenvironment.**

**(A)** KEGG pathway analysis shows the significantly downregulated pathways (*P*<0.05) in tumor core. **(B)** The level of genes in arginine metabolic process gene sets in the region of tumor core versus margin are shown as a heat map. TC, tumor core. TM, tumor margin. **(C)** Representative H&E staining of tumors for mass spectrometry imaging. **(D)** Representative mass spectrometry imaging showed the distribution of *N*^1^-Acetylspermidine and *N*^1^-Acetylspermine in 231/WT, 231/Rab27a KD and 4T1/WT tumors. **(E)** Serum putrescine concentrations of healthy people and breast cancer patients (n = 5) or NSG mice with or without 231 cells xenograft (n = 9) were measured by ELISA kit. Data are presented as mean ± s.d., unpaired two-tailed Student’s t-test. **(F)** Western blots verified the Rab27a knockout or knock down outcomes in 4T1 and MDA-MB-231 cells. **(G)** Quantification of EV counts derived from Rab27a knockout or knock down outcomes in 4T1 and MDA-MB-231 cells by nano-flow cytometry. Data are presented as mean ± s.d., n = 3 biological replicates, unpaired two-tailed Student’s t-test. **(H)** Western blots analysis of the AGMAT levels in human cancer-associated fibroblasts (hCAFs) treated with the indicated EVs. **(I)** Putrescine concentrations of 231/Rab27a KD and 4T1/Rab27a KO tumor core and margin were measured by ELISA kit. Data are presented as dots, n = 6 for 231/Rab27a KD, n = 5 biological replicates for 4T1/Rab27a KO, paired two-tailed Student’s t-test.


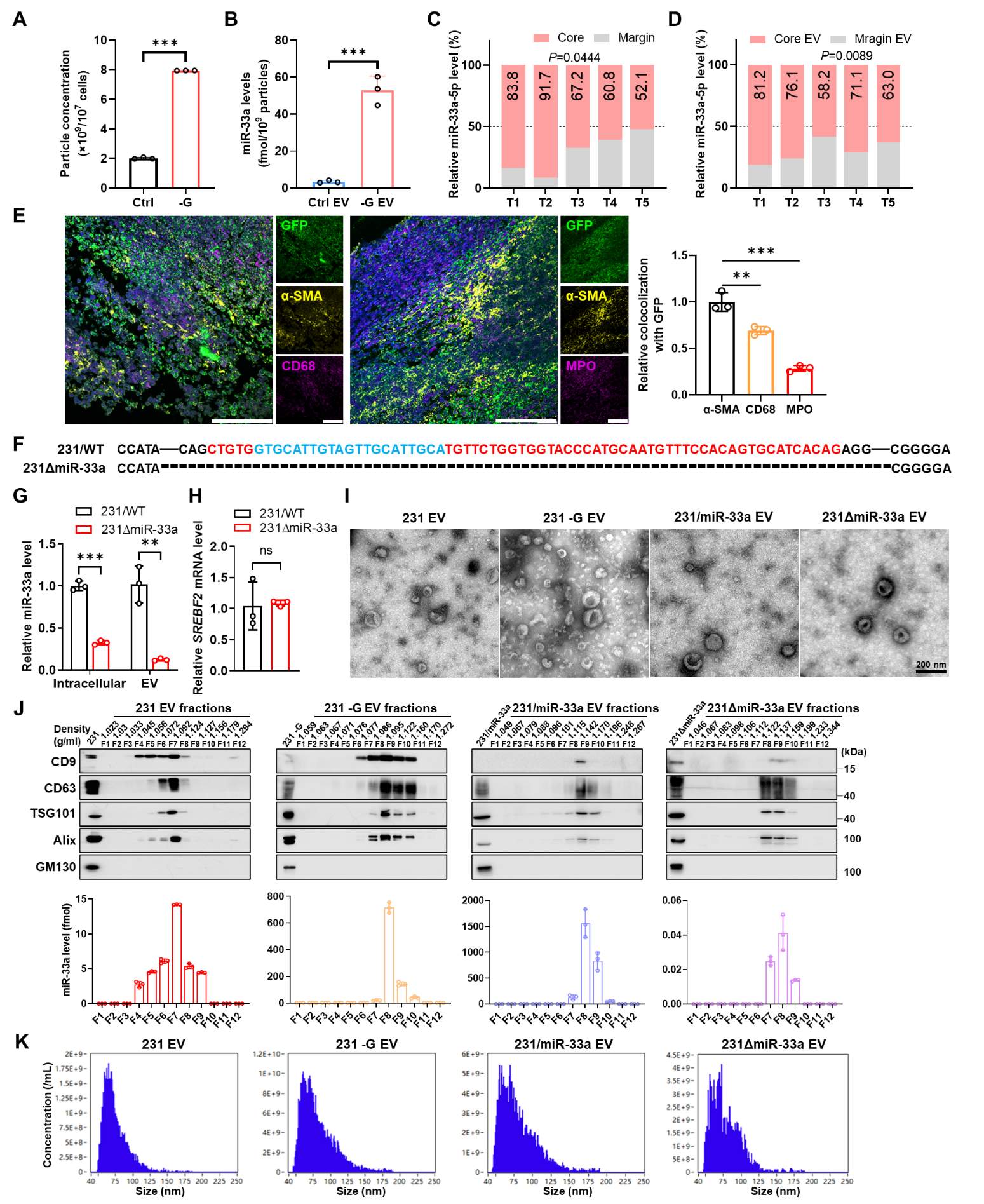


**Figure 2: Starvation-induced-miR-33a inhibits putrescine production by targeting AGMAT in tumor stroma.**

**(A)** Quantification of EV counts derived from Ctrl or -G medium-treated MDA-MB-231 cells by nano-flow cytometry. Data are presented as mean ± s.d., n = 3 biological replicates, unpaired two-tailed Student’s t-test. **(B)** Levels of RT-qPCR-determined miR-33a (normalized to a synthetic miR-33a determined standard curve) in 231/Ctrl EVs or 231/-G EVs. Data are presented as mean ± s.d., n = 3 biological replicates, unpaired two-tailed Student’s t-test. **(C)** Relative miR-33a levels of 231/WT xenograft tumor in core and margin regions determined by RT-qPCR. Data are presented as mean ± s.d., n=5, paired two-tailed Student’s t-test. **(D)** RT-qPCR-determined miR-33a levels of EVs from 231/WT xenograft tumor in core and margin regions. Data are presented as mean ± s.d., n=5, paired two-tailed Student’s t-test. **(E)** Representative mIHC analysis of 4T1/WT tumor samples stained for α-SMA (yellow), CD68 (purple), MPO (purple) and DAPI (blue). Lck-GFP labeled 4T1 cells and EVs were green. Scale bar, 200 μm. Quantification of mIHC staining presented as mean ± s.d., n = 3 mice, unpaired two-tailed Student’s t-test. **(F)** Sanger sequencing showing genetic knockout of *hsa*-miR-33a-5p gene in 231ΔmiR-33a cells. Dashes indicate the region deleted by the CRISPR-Cas9 genome editing system. **(G)** The intracellular and EV miR-33a levels were detected from 231/WT or 231∆miR-33a cells by RT-qPCR. Data are presented as mean ± s.d., n = 3 biological replicates, unpaired two-tailed Student’s t-test. **(H)** Relative *SREBF2* RNA levels of 231/WT and 231ΔmiR-33a were detected by RT-qPCR. Data are presented as mean ± s.d., n = 3 biological replicates, unpaired two-tailed Student’s t-test. **(I)** Representative transmission electron microscope (TEM) images of EVs. **(J)** Levels of indicated EV proteins (top) and RT-qPCR-determined miR-33a (bottom, normalized to a synthetic miR-33a determined standard curve) in OptiPrep gradient fractions (n=3 biological replicates). **(K)** Nano flow cytometry showing size distribution and concentration in EVs derived from different cell lines.


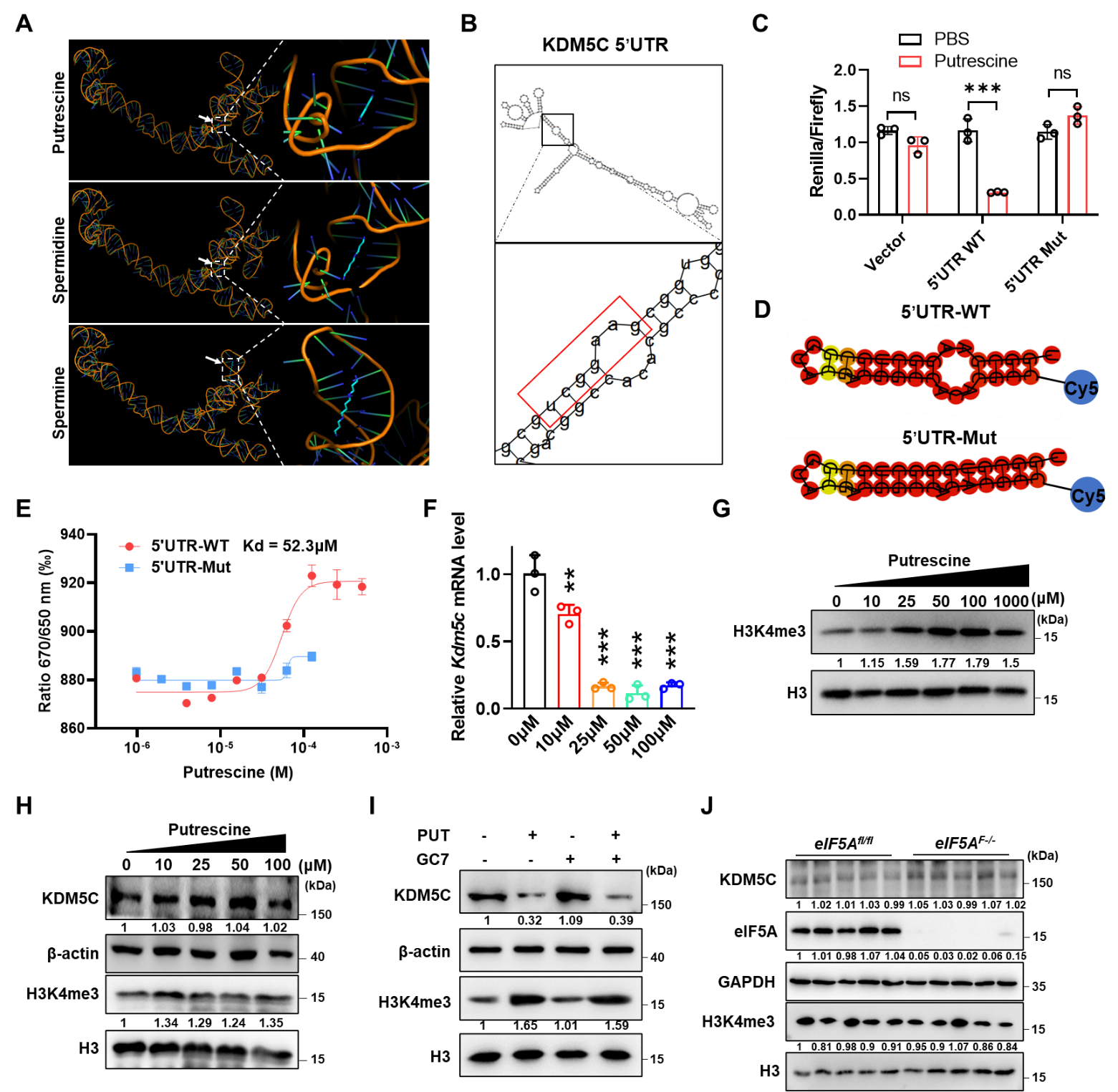


**Figure 3: Putrescine enhances H3K4 tri-methylation by inhibiting KDM5C expression.**

**(A)** Docking model displaying potential interaction between 5’ UTR of KDM5C gene (sequence from UCSC) with putrescine, spermidine and spermine. **(B)** Schematic diagram shows the secondary structure of the 5’UTR of KDM5C. The red frame shows the predicted binding site of putrescine. **(C)** Responsiveness of the human WT or mutant KDM5C 5'UTR reporters to putrescine (25 μM). Data are presented as mean ± s.d., n = 3 biological replicates, unpaired two-tailed Student’s t-test. **(D)** Schematic diagram shows the secondary structure of the Cy5 labeled WT or mutant 5’UTR of KDM5C. **(E)** Microscale thermophoresis (MST) to detect the binding affinity between putrescine and the Cy5 labeled WT or mutant 5’UTR of KDM5C (Sigmoidal, 4PL, X is log(concentration), Kd = 52.3µM, n=3 biological replicates). **(F)** The expression of *Kdm5c* from putrescine treated NIH3T3 (-R medium) was analyzed by RT-qPCR. Data are presented as mean ± s.d., n = 3 biological replicates, one-way ANOVA, Dunnett’s multiple comparisons test. **(G)** The expression of H3K4me3 gradient treated with concentration of putrescine was detected by western blots. **(H)** NIH3T3 with stable expression KDM5C cells were treated with putrescine. KDM5C and H3K4me3 protein levels were examined. **(I)** The expression of KDM5C and H3K4me3 from putrescine or GC7 treated mouse cancer-associated fibroblasts (mCAFs) was determined. Putrescine (PUT, 25 μM) treated for 48h and N1-guanyl-1,7-diaminoheptane (GC7) was added to cells for 24 h at a concentration of 10 μM. **(J)** Protein levels of mCAFs were examined from eIF5A fibroblast conditional knock out (*eIF5A^F-/-^*) and control (*eIF5A^fl/fl^*) mice with E0771 xenograft.


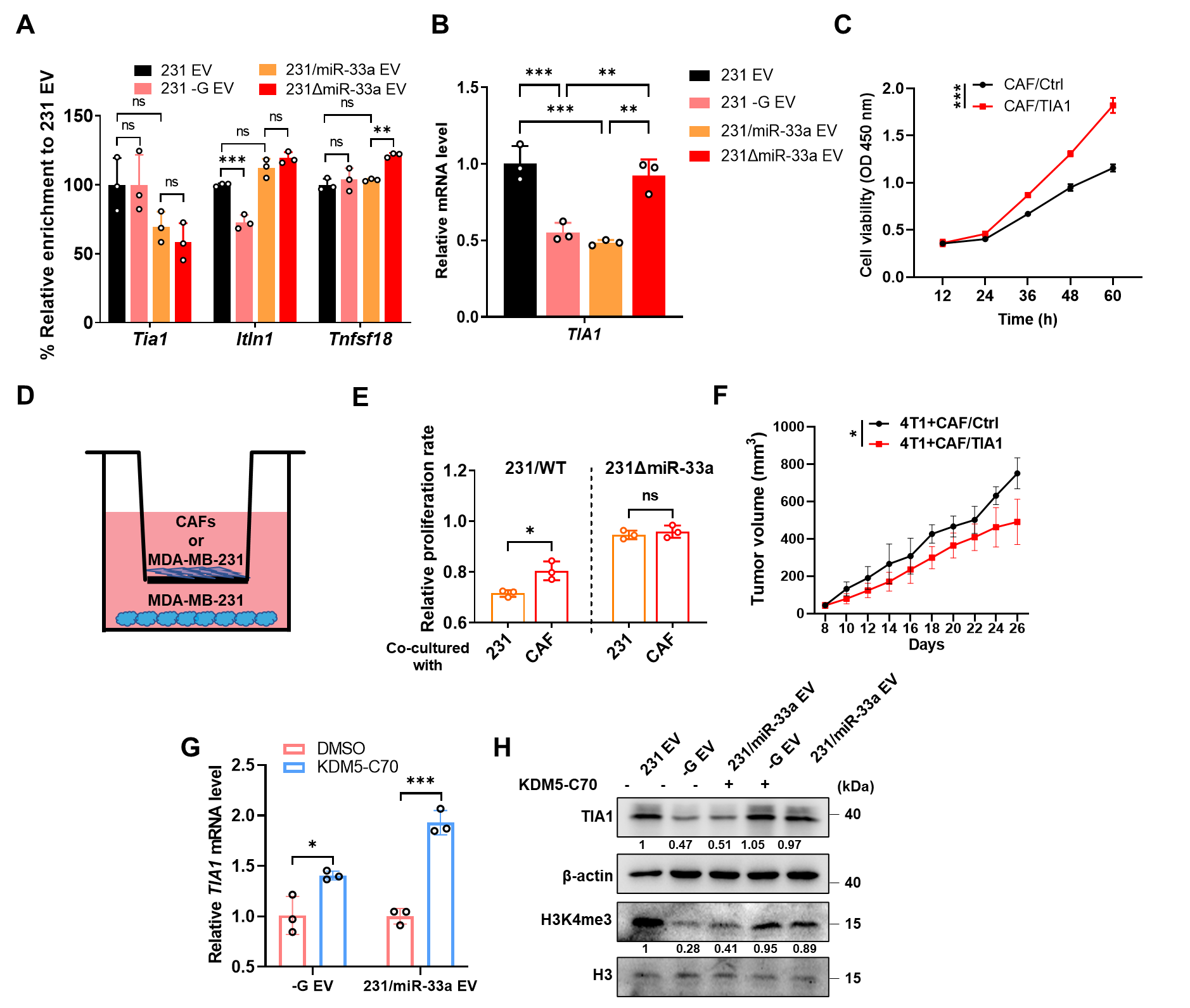


**Figure 4: MiR-33a reprograms epigenetic profile to induce stress granule disassembling in CAF.**

**(A)** H3K27me3 ChIP-qPCR at the *Tia1*, *Itln1* and *Tnfsf18* locus. Data are presented as mean ± s.d., n = 3 biological replicates, one-way ANOVA, Tukey’s multiple comparisons test.  **(B)** RNA levels of *TIA1* in hCAFs from indicated groups were analyzed by RT-qPCR. Data are presented as mean ± s.d., n = 3 biological replicates, one-way ANOVA, Tukey’s multiple comparisons test. **(C)** The proliferation of CAF/Ctrl or CAF/TIA1 cells were determined by CCK-8 kit. Data are presented as mean ± s.d., n = 3 biological replicates, two-way ANOVA, Sidak’s multiple comparisons test. **(D)** The schematic diagram showing MDA-MB-231 co-cultured with MDA-MB-231 or hCAFs by transwell. Glucose free medium was supplied.  **(E)** 231/WT or 231ΔmiR-33a cells were cocultured with hCAFs or 231 followed by cell proliferation rate detection using Cell Counting Kit-8. Data are presented as mean ± s.d., n = 3 biological replicates, unpaired two-tailed Student’s t-test. Glucose free medium was supplied. **(F)** Female BALB/c mice received a mammary fat pad injection of 2×10^5^ 4T1 cells mixed with 10^6^ CAFs or CAF/TIA1. The tumor volume was measured. Data are presented as mean ± s.d., n = 5, two-way ANOVA, Sidak’s multiple comparisons test. **(G)** The expression of *TIA1* in hCAFs from indicated groups was analyzed by RT-qPCR. Data are presented as mean ± s.d., n = 3 biological replicates, unpaired two-tailed Student’s t-test. **(H)** Western blots showing TIA1 and H3K4me3 levels of EVs treated hCAFs followed by KDM5C inhibitor (KDM5 histone demethylase inhibitor, KDM5-C70, 50 μM) or vehicle treatment.


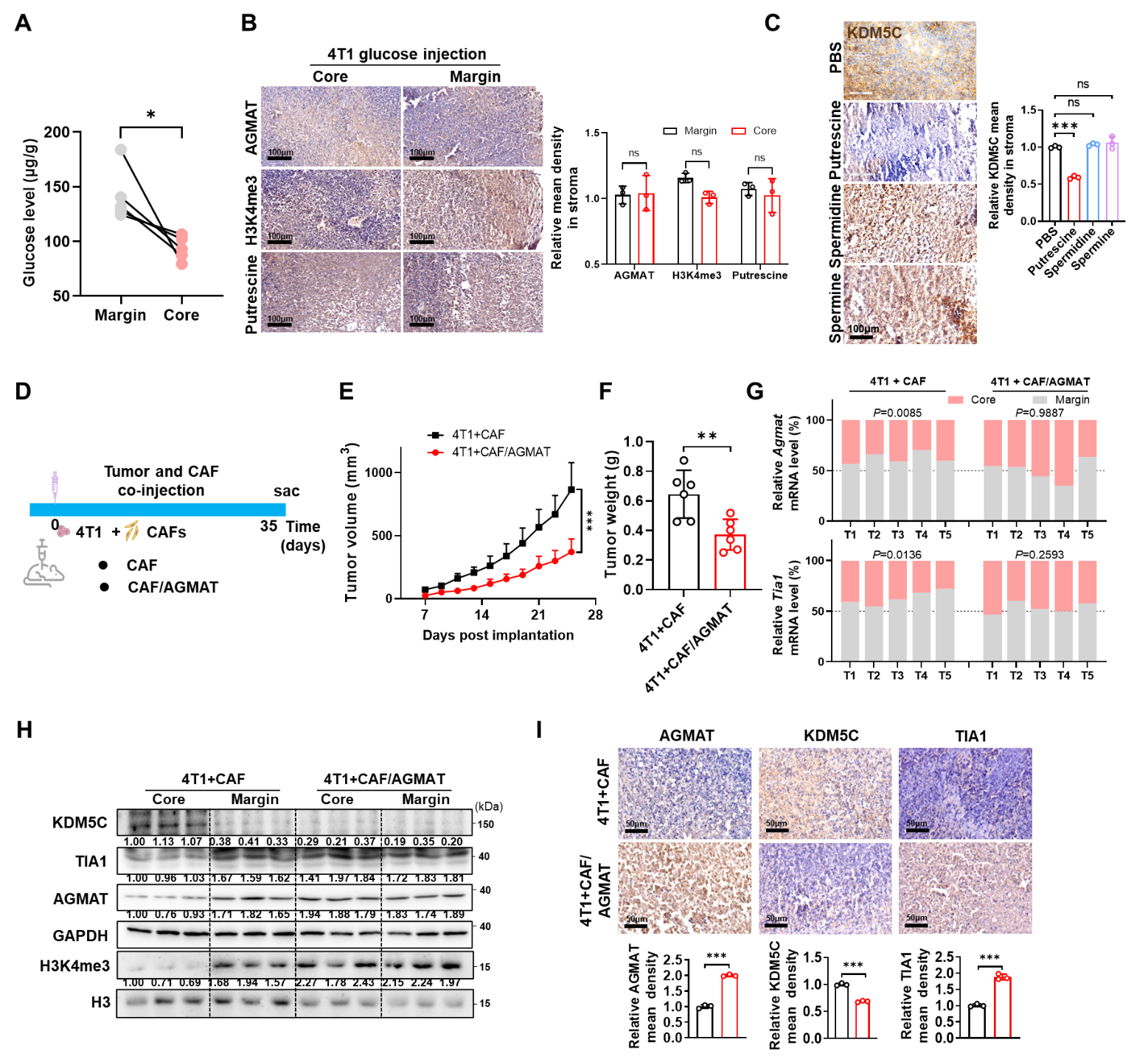


**Figure 5: Nutrients injection blocks the effect of miR-33a in the tumor core region.**

**(A)** The glucose concentrations of core and margin regions from BALB/c 4T1 PBS injected tumors were detected. Data are presented as dots, n = 5 mice, paired two-tailed Student’s t-test. **(B)** Representative IHC images showing AGMAT, H3K4me3 and putrescine staining of 4T1 glucose injected tumor in core and margin regions. Quantification of IHC staining presented as mean ± s.d., n = 3 mice, paired two-tailed Student’s t-test. Scale bar, 100 μm. **(C)** Representative IHC images showing KDM5C staining of 4T1 polyamine or PBS injected tumor in TC. Quantification of IHC staining presented as mean ± s.d., n = 3 mice, one-way ANOVA, Dunnett’s multiple comparisons test. Scale bar, 100 μm. **(D)** The strategy of BALB/c 4T1 and mCAFs co-injection xenograft tumors was shown. **(E)** Tumor volume of 4T1 co-injected with WT or AGMAT overexpressed mCAFs. Data were presented as mean ± s.d., n = 6 mice, two-way ANOVA, Sidak’s multiple comparisons test. **(F)** Tumor weight of 4T1+CAF and 4T1+CAF/AGMAT was weighed at the end of the experiment. Data are presented as mean ± s.d., n = 6, unpaired two-tailed Student’s t-test. **(G)** RT-qPCR-determined AGMAT and TIA1 levels from indicated tumor CAFs. Data are presented as mean ± s.d., n = 5 biological replicates, paired two-tailed Student’s t-test. **(H)** Core and margin CAFs of the 4T1+CAF and 4T1+CAF/AGMAT tumors were harvested and protein levels were detected by western blots. **(I)** Representative IHC images showing AGMAT, KDM5C and TIA1 staining of two group tumors. Quantification of IHC staining presented as mean ± s.d., n = 3 mice, unpaired two-tailed Student’s t-test. Scale bar, 50 μm.


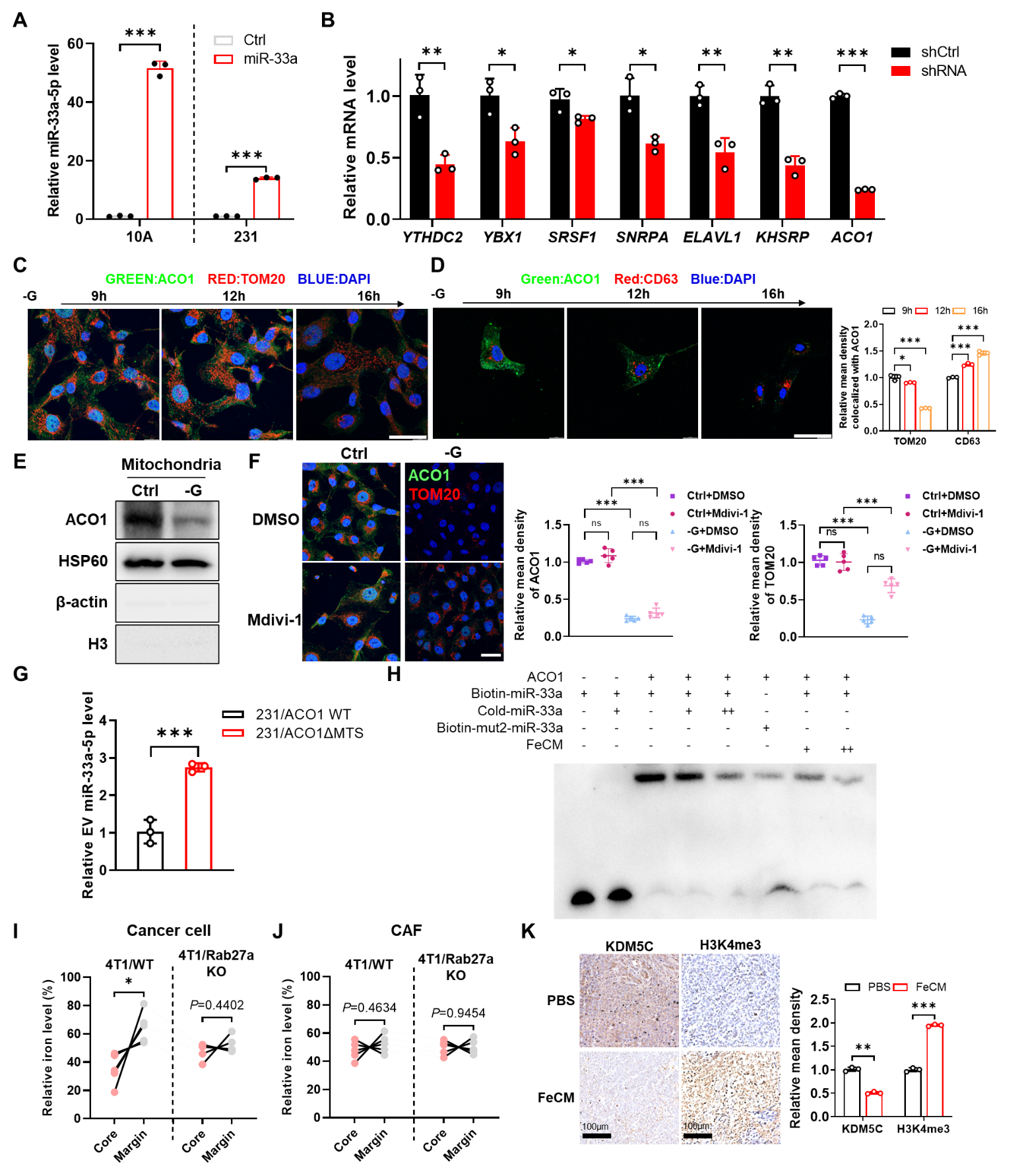


**Figure 6:** **ACO1 assists miR-33a secretion under low iron level.**

**(A)** MiR-33a levels of WT or *hsa*-miR-33a-5p overexpressed in MCF-10A (left) and 231 (right). Data are presented as mean ± s.d., n = 3 biological replicates, unpaired two-tailed Student’s t-test. **(B)** The RNA levels of putative RNA binding proteins from 231/miR-33a knockdown cells were measured by RT-qPCR. Data are presented as mean ± s.d., n = 3 biological replicates, unpaired two-tailed Student’s t-test. **(C-D)** Representative immunofluorescence images of 231 cells treated with time gradient glucose starvation were captured by confocal laser scanning microscope. TOM20, mitochondrial marker. CD63, MVB marker. Scale bar, 50 μm. Quantification of colocalization presented as mean ± s.d., 3 different fields, unpaired two-tailed Student’s t-test. **(E)** Mitochondria derived from 231/Ctrl or 231 -G and indicated proteins were detected. **(F)** Representative immunofluorescence images of 231 cells treated with glucose starvation and Mdivi-1 (10 μM) were captured by confocal laser scanning microscope. TOM20, mitochondrial marker. CD63, MVB marker. Scale bar, 50 μm. Quantification of colocalization presented as mean ± s.d., 5 different fields, one-way ANOVA, Tukey’s multiple comparisons test. **(G)** MiR-33a levels were detected in EVs derived from 231/ACO1 WT or 231/ACO1ΔMTS (mitochondrial targeting sequences, deletion of 50 amino acids (1-50)) by RT-qPCR. Data are presented as mean ± s.d., n = 3 biological replicates, unpaired two-tailed Student’s t-test. **(H)** Electrophoresis mobility shift assay (EMSA) showing the binding of ACO1 to wild-type miR-33a. **(I)** Relative iron levels within tumor core and margin regional cancer cells derived from 4T1/WT (left) and 4T1/Rab27a KO (right) tumors. Data are presented as mean ± s.d., n = 5, paired two-tailed Student’s t-test. **(J)** Relative iron levels within tumor core and margin regional mCAF cells derived from 4T1/WT (left) and 4T1/Rab27a KO (right) tumors. Data are presented as mean ± s.d., n = 5, paired two-tailed Student’s t-test. **(K)** Representative IHC images showing KDM5C and H3K4me3 staining of 4T1 ferric carboxymaltose (FeCM) injected tumor in TC. Quantification of IHC staining presented as mean ± s.d., n = 3 mice, paired two-tailed Student’s t-test. Scale bar, 100 μm.


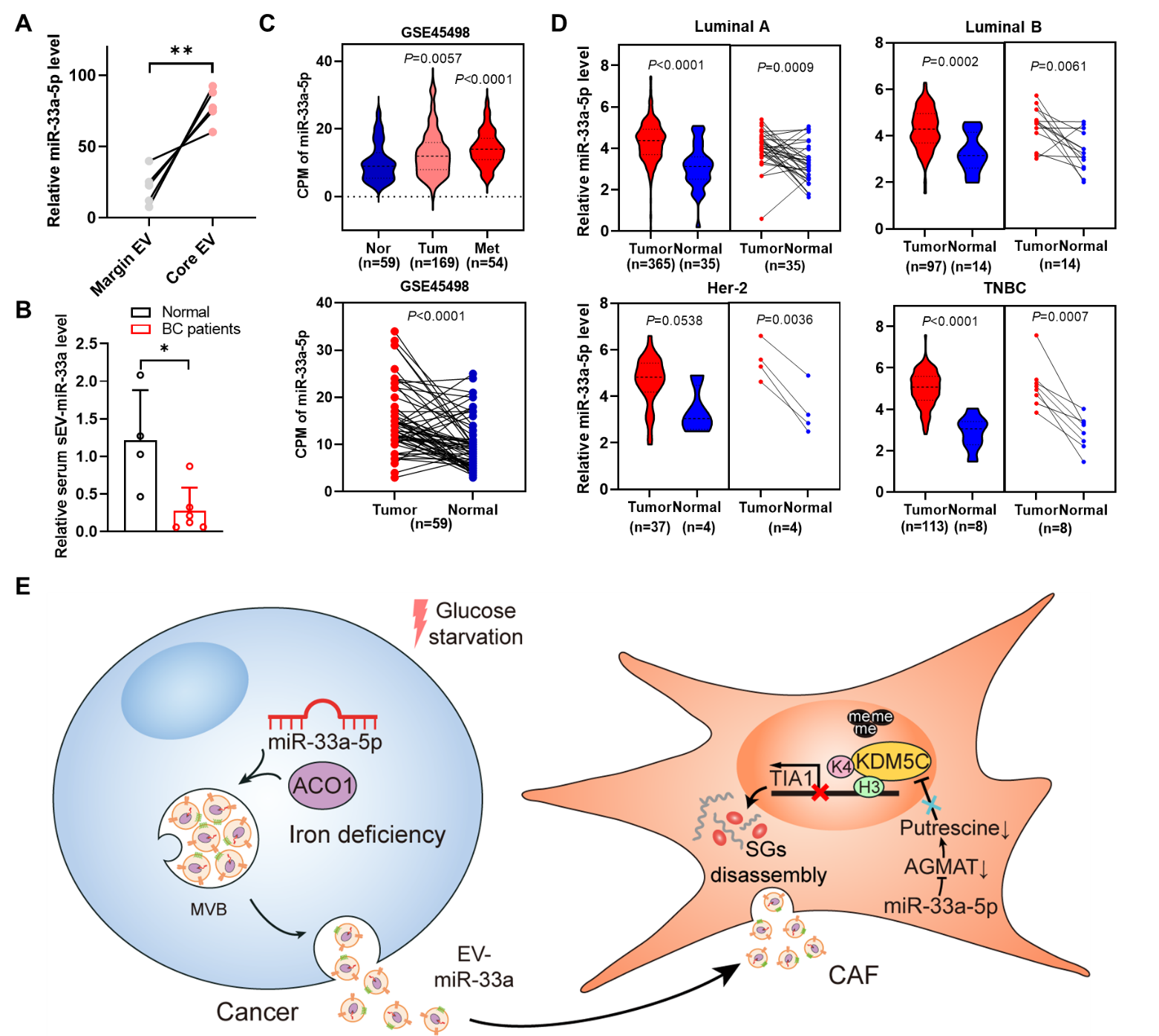


**Figure 7: MiR-33a/AGMAT axis widely exists in breast cancer patients.**

**(A)** Relative miR-33a levels of EVs (n=5) derived from BC patient tumor core and margin regions determined by RT-qPCR. Data are presented as mean ± s.d., paired two-tailed Student’s t-test. **(B)** RT-qPCR-determined miR-33a levels in EVs derived from serum of normal (n=4) and breast cancer patients (n=6) (Mann-Whitney test). **(C)** The *hsa*-miR-33a-5p levels were assessed from GSE45498 database. Boxplots show median, quartiles, min, and max. Data are presented as mean ± s.d., two-tailed Mann-Whitney test (left) and paired two-tailed Student’s t-test (right). **(D)** Relative *hsa*-miR-33a-5p levels in BRCA tumors and normal mammary tissues from TCGA databases among breast cancer subtypes (luminal A, luminal B, HER2 and triple-negative). Statistical significance was assessed using two-tailed Mann-Whitney test (left) and paired two-tailed Student’s t-test (right). **(E)** Schema summaries the crosstalk between cancer derived miR-33a and CAF in this study.

**Supplementary Table 1: Primer sequences used in RT-qPCR.**

| **Gene name** | **Forward primer** | **Reverse primer** |
| --- | --- | --- |
| Human *KDM5C* | GGGTCCGACGATTTCCTACC | ATGCCCGATTTCTCTGCGATG |
| Human *ACO1* | TATGGCTCCCGCCGAGGTAA | ACCGCTCAGCAGCATCAAACA |
| Human *ELAVL1* | TTTGGGCGGATCATCAACTCG | AAACTGGTAATTGCCTCTTCTGC |
| Human *PUM2* | CCGCCATCACTTTCATCACAT | TCAGACCTATTATACCGAAGC |
| Human *KHSRP* | CCGAGGTGGCGAGAATGTGAA | GGCCTGGTCCAACTGGGCAGA |
| Human *SRSF1* | CGCCCTTCGCCTTCGTTGAGT | CGAGGAAACTCCACCCGCAGA |
| Human *YBX1* | CGGGGACAAGAAGGTCATCGC | CCTGGGGTTATTCTTCTTTATG |
| Human *SNRPA* | CACCGAATCACATCTTGTTCCTCAC | CTGCCTGTACCTCATTGTCAAACTC |
| Human *YTHDC2* | TCTTGGAGGTCAAATAATAGTAGGA | CTTCTGAGGTGGGTGTAATGC |
| Human *SREBF2* | AACGGTCATTCACCCAGGTC | GGCTGAAGAATAGGAGTTGCC |
| Human *TIA1* | GATGCCCGAGTGGTAAAAGAC | CCCATCTGTTGAATGGCGTTT |
| Human 18S rRNA (internal | CTACCACATCCAAGGAAGGCA | TTTTTCGTCACTACCTCCCCG |
| control) |  |  |
| Mouse *Agmat* | ACACCAACACCACGGACAAAC | CAGTCTTCAGCCAGGACCACA |
| Mouse *Kdm5c* | GAGGCCCAGACAAGAGTGAAA | TTGGGAATCTTTAAGGATGAGCC |
| Mouse *Aco1* | AGAACCCATTTGCACACCTTG | AGCGTCCGTATCTTGAGTCCT |
| Mouse *Tia1* | CACACAGCGTTCACAAGATCA | GGTAGCCATGTCTTTTACCACA |
| Mouse *Kdm1a* | AGGAATCGCACATTGCAGTTA | ACAGTATCGCTGTTGTAAGGC |
| Mouse *Kdm5a* | CACAGACCCGCTGAGTTTTAT | CTTCACAGGCAAATGGAGGTT |
| Mouse *Kdm5b* | CTGGGAAGAGTTCGCGGAC | CGCGGGGTGAAATGAAGTTTAT |
| Mouse *Kdm5d* | CCAGGATCTGACGACTTTCTACC | TTCTCCGCAATGGGTCTGATT |
| Mouse *Kmt2a* | GCAGATTGTAAGACGGCGAG | GAGAGGGGGTGTTCCTTCCTT |
| Mouse *Kmt2b* | GATGAGAATGGCTCGTTGTGG | TCTATCTTGTCACACTTCCGGTA |
| Mouse *Kmt2c* | GCCACAGTCCCCTATGAAGGA | GGGAAGGATATGGGAGGTGATG |
| Mouse *Kmt2d* | TCATTGTTCTGCTTGGCTTGGT | TGCAGCGAATTTGTCCAGGT |
| Mouse *Setd1a* | TGCTGTCCCTCGTAGACTGG | GGCTCTTTCCGTTTTACCTTGA |
| Mouse *Setd1b* | TCCTCAAGCTCCGACAAGGAT | CGTCGATGTCTGAATCAATCTGG |
| Mouse *Actb* (internal control) | CGAGGCCCAGAGCAAGAGAG | CGGTTGGCCTTAGGGTTCAG |

**Supplementary Table 2: Antibody information.**

| **Antibody name** | **Source** | **Catalog #** |
| --- | --- | --- |
| Mouse monoclonal anti-AGMAT antibody | Santa Cruz | sc-166414 |
| Mouse monoclonal anti-TOM20 antibody | Santa Cruz | sc-17764 |
| Rabbit polyclonal anti-MPO antibody | Proteintech | 22225-1-AP |
| Rabbit polyclonal anti-smooth muscle actin antibody | Proteintech | 14395-1-AP |
| Rabbit polyclonal anti-Aconitase 1 antibody | Proteintech | 12406-1-AP |
| Rabbit polyclonal anti-TIA1 antibody | Proteintech | 12133-2-AP |
| Mouse monoclonal anti-G3BP1 antibody | Proteintech | 66486-1-Ig |
| Rabbit polyclonal anti-Alix antibody | Proteintech | 12422-1-AP |
| Rabbit polyclonal anti-CD9 antibody | Proteintech | 20597-1-AP |
| Rabbit polyclonal anti-CD63 antibody | Proteintech | 25682-1-AP |
| Rabbit polyclonal anti-TSG101 antibody | Proteintech | 28283-1-AP |
| Rabbit polyclonal anti-GOLGA2/GM130 antibody | Proteintech | 11308-1-AP |
| Rabbit polyclonal anti-GAPDH antibody | Proteintech | 10494-1-AP |
| Mouse monoclonal anti-Beta Actin antibody | Proteintech | 66009-1-Ig |
| Mouse monoclonal anti-Histone H3 (di methyl K9) | Abcam | ab1220 |
| Rabbit monoclonal anti-Histone H3 (tri methyl K9) | Abcam | ab176916 |
| Rabbit monoclonal anti-Histone H3 (mono methyl K14) | Abcam | ab202416 |
| Rabbit polyclonal anti-Histone H3 (di methyl K27) | Abclonal | A2362 |
| Rabbit polyclonal anti-Histone H3 (tri methyl K27) | Abclonal | A2363 |
| Rabbit polyclonal anti-CD68 antibody | Abclonal | A13286 |
| Rabbit polyclonal anti-Tri-Methyl-Histone H3 (Lys4)/H3K4me3 antibody | Affinity | DF6935 |
| Rabbit polyclonal anti-KDM5C/Jarid1C/SMCX antibody | Affinity | DF13631 |
| Rabbit polyclonal anti-Histone H3 antibody | Cell Signaling Technology | 9715 |
| Rabbit anti Myc-Tag pAb | Abclonal | AE009 |
| Rabbit polyclonal anti-putrescine antibody | FineTest | FNab09929 |
| Sheep Anti-Digoxigenin Fab fragments Antibody, AP Conjugated | Roche | 11093274910 |
| Goat anti-mouse IgG (H+L) secondary antibody, HRP | Thermo Fisher Scientific | 31430 |
| Goat anti-rabbit IgG (H+L) secondary antibody, HRP | Thermo Fisher Scientific | 31460 |
| Alexa Fluor 488 AffiniPure Donkey anti-Rabbit IgG (H+L) secondary antibody | Yeasen | 34206ES60 |
| Alexa Fluor 594-conjugated AffiniPure Goat Anti-Mouse IgG (H+L) secondary antibody | Abclonal | AS077 |
| Rabbit IgG | Proteintech | B900610 |

**Supplementary Table 3: Upregulated EV proteomics in TC versus TM-derived EVs and 231 -G versus 231 EVs.**

| **Protein name** | **TM-EV** | **TC-EV** | **p value** | **Fc** | **Protein name** | **231 EV** | **231 EV** | **231-G EV** | **FC** | **TC EV vs TM EV**  **231-G EV vs 231 EV** |
| --- | --- | --- | --- | --- | --- | --- | --- | --- | --- | --- |
| Ighv9 | 0 | 199334.667 | 0 | 33 | TMEM183A | 1 | 1 | 19.174 | 19.174 | Rbbp7 |
| Cd101 | 0 | 9954.000 | 0 | 33 | HSP90AA4P | 1 | 1 | 16.061 | 16.061 | Rptor |
| Tyrobp | 0 | 11966.667 | 0 | 33 | ASPM | 1 | 1 | 15.538 | 15.538 | Stxbp5 |
| Cdcp1 | 0 | 37482.333 | 0 | 33 | ARHGAP12 | 1 | 1 | 14.749 | 14.749 | Asap1 |
| C1s2 | 0 | 150493.333 | 0 | 33 | ANXA13 | 1 | 1 | 14.365 | 14.365 | Fgfbp1 |
| Actn1 | 0 | 14998.000 | 0 | 33 | UGT2A1 | 1 | 1 | 14.298 | 14.298 | Frmd8 |
| Septin8 | 0 | 12737.200 | 0 | 33 | TNFRSF11B | 1 | 1 | 13.291 | 13.291 | Gak |
| Ddx3x | 0 | 7315.633 | 0 | 33 | AKNA | 1 | 1 | 12.912 | 12.912 | Golm1 |
| Anp32a | 0 | 22455.667 | 0 | 33 | KRT17 | 1 | 1 | 12.403 | 12.403 | Psmd5 |
| Clcn7 | 0 | 14104.633 | 0 | 33 | CYP24A1 | 1 | 1 | 12.24 | 12.24 | Pno1 |
| Apob | 0 | 4808.000 | 0 | 33 | NID1 | 1 | 1 | 11.808 | 11.808 | Wdr55 |
| Sqor | 0 | 59147.667 | 0 | 33 | BICD1 | 1 | 1 | 11.562 | 11.562 | H2ax |
| Tspan15 | 0 | 50645.667 | 0 | 33 | TTN | 1 | 1 | 11.275 | 11.275 | Atox1 |
| Pkd2 | 0 | 7453.367 | 0 | 33 | PZP | 1 | 1 | 10.511 | 10.511 | Eif3m |
| Yme1l1 | 0 | 20954.000 | 0 | 33 | ANK2 | 1 | 1 | 10.042 | 10.042 | Top1 |
| Mtnd5 | 0 | 24397.333 | 0 | 33 | CCDC91 | 1 | 1 | 9.97 | 9.97 | Pzp |
| Prf1 | 0 | 5034.300 | 0 | 33 | EPSTI1 | 1 | 1 | 9.768 | 9.768 | Gpx4 |
| Casp1 | 0 | 9864.000 | 0 | 33 | MROH2A | 1 | 1 | 9.508 | 9.508 | Gys1 |
| Timp3 | 0 | 24562.333 | 0 | 33 | CDCA2 | 1 | 1 | 9.347 | 9.347 | Cstf2 |
| Hmga2 | 0 | 16929.333 | 0 | 33 | IFT74 | 1 | 1 | 8.988 | 8.988 |  |
| Polr2i | 0 | 7228.000 | 0 | 33 | TTC5 | 1 | 1 | 8.921 | 8.921 |  |
| Rap2b | 0 | 59202.000 | 0 | 33 | ARPIN-AP3S2 | 1 | 1 | 8.441 | 8.441 |  |
| Rhob | 0 | 10910.333 | 0 | 33 | NAP1L1 | 1 | 1 | 8.311 | 8.311 |  |
| Prkacb | 0 | 16461.500 | 0 | 33 | OSBPL5 | 1 | 1 | 8.166 | 8.166 |  |
| Vamp7 | 0 | 15546.700 | 0 | 33 | GDPGP1 | 1 | 1 | 7.745 | 7.745 |  |
| Casp7 | 0 | 9129.000 | 0 | 33 | PIK3C3 | 1 | 1 | 7.677 | 7.677 |  |
| Col8a1 | 0 | 57143.667 | 0 | 33 | EIF3J | 1 | 1 | 7.671 | 7.671 |  |
| Jup | 0 | 15403.867 | 0 | 33 | RALGPS2 | 1 | 1 | 7.636 | 7.636 |  |
| Fcna | 0 | 23081.667 | 0 | 33 | EIF3K | 1 | 1 | 7.626 | 7.626 |  |
| H2 | 0 | 24757.667 | 0 | 33 | CYP4F22 | 1 | 1 | 7.435 | 7.435 |  |
| Ubqln1 | 0 | 20018.333 | 0 | 33 | CNIH4 | 1 | 1 | 7.428 | 7.428 |  |
| Gypa | 0 | 43450.333 | 0 | 33 | ACTRT1 | 1 | 1 | 7.085 | 7.085 |  |
| Khsrp | 0 | 11430.333 | 0 | 33 | SCLT1 | 1 | 1 | 7.065 | 7.065 |  |
| Lipg | 0 | 8128.133 | 0 | 33 | MYO1A | 1 | 1 | 7.012 | 7.012 |  |
| Tom1 | 0 | 18046.700 | 0 | 33 | SMG1 | 1 | 1 | 6.827 | 6.827 |  |
| Clpb | 0 | 20835.000 | 0 | 33 | MYOT | 1 | 1 | 6.708 | 6.708 |  |
| Diaph1 | 0 | 12271.800 | 0 | 33 | LSM4 | 1 | 1 | 6.674 | 6.674 |  |
| Rps4l | 0 | 19415.000 | 0 | 33 | NMT2 | 1 | 1 | 6.654 | 6.654 |  |
| Prkaa1 | 0 | 5408.900 | 0 | 33 | WFIKKN1 | 1 | 1 | 6.606 | 6.606 |  |
| Tmem256 | 0 | 102681.333 | 0 | 33 | GRAMD4 | 1 | 1 | 6.602 | 6.602 |  |
| IgG1 | 0 | 6792.970 | 0 | 33 | PARP9 | 1 | 1 | 6.409 | 6.409 |  |
| Rbbp4 | 0 | 23031.333 | 0 | 33 | RTN4 | 1 | 1 | 6.367 | 6.367 |  |
| H2A | 0 | 102950.000 | 0 | 33 | SLAIN1 | 1 | 1 | 6.298 | 6.298 |  |
| mKIAA0024 | 0 | 8159.267 | 0 | 33 | CD38 | 1 | 1 | 6.19 | 6.19 |  |
| Fgr | 0 | 57369.000 | 0 | 33 | RAB26 | 1 | 1 | 6.18 | 6.18 |  |
| Smc3 | 0 | 8470.600 | 0 | 33 | ZNRD2 | 1 | 1 | 6.116 | 6.116 |  |
| Bpnt2 | 0 | 43525.667 | 0 | 33 | RGPD3 | 1 | 1 | 6.006 | 6.006 |  |
| Slc7a6 | 0 | 48088.333 | 0 | 33 | RTKN | 1 | 1 | 5.965 | 5.965 |  |
| Pafah1b2 | 0 | 21107.333 | 0 | 33 | FGD6 | 1 | 1 | 5.947 | 5.947 |  |
| Uap1l1 | 0 | 8558.233 | 0 | 33 | WDR46 | 1 | 1 | 5.886 | 5.886 |  |
| Mthfd1 | 0 | 24567.000 | 0 | 33 | POLQ | 1 | 1 | 5.848 | 5.848 |  |
| Ccz1 | 0 | 117111.000 | 0 | 33 | AK8 | 1 | 1 | 5.728 | 5.728 |  |
| Acta2 | 0 | 112920.000 | 0 | 33 | BET1 | 1 | 1 | 5.706 | 5.706 |  |
| Pak2 | 0 | 11391.900 | 0 | 33 | CLUAP1 | 1 | 1 | 5.662 | 5.662 |  |
| Cfhr2 | 0 | 30671.833 | 0 | 33 | DES | 1 | 1 | 5.652 | 5.652 |  |
| Eif4b | 0 | 11065.000 | 0 | 33 | IPO7 | 1 | 1 | 5.624 | 5.624 |  |
| Impa2 | 0 | 14235.333 | 0 | 33 | SYTL2 | 1 | 1 | 5.61 | 5.61 |  |
| Rragc | 0 | 46660.667 | 0 | 33 | FAM102A | 1 | 1 | 5.606 | 5.606 |  |
| Selplg | 0 | 245856.667 | 0 | 33 | KIF18B | 1 | 1 | 5.57 | 5.57 |  |
| Chmp3 | 0 | 78427.333 | 0 | 33 | BCORL1 | 1 | 1 | 5.517 | 5.517 |  |
| Ndufa2 | 0 | 31650.333 | 0 | 33 | ATP7A | 1 | 1 | 5.476 | 5.476 |  |
| RTRAF | 0 | 5661.900 | 0 | 33 | HERC5 | 1 | 1 | 5.451 | 5.451 |  |
| Ndufb9 | 0 | 62550.333 | 0 | 33 | SRPRA | 1 | 1 | 5.359 | 5.359 |  |
| Eif3e | 0 | 18237.000 | 0 | 33 | ANKLE2 | 1 | 1 | 5.316 | 5.316 |  |
| Agpat3 | 0 | 14976.333 | 0 | 33 | INTS1 | 1 | 1 | 5.24 | 5.24 |  |
| Chmp5 | 0 | 40544.667 | 0 | 33 | TGFB1 | 1 | 1 | 5.182 | 5.182 |  |
| Cmpk1 | 0 | 8351.333 | 0 | 33 | H3C15 | 1 | 1 | 5.118 | 5.118 |  |
| Mfsd1 | 0 | 40071.000 | 0 | 33 | NUDT4 | 1 | 1 | 5.113 | 5.113 |  |
| Abcb10 | 0 | 37913.667 | 0 | 33 | RPL10 | 1 | 1 | 5.095 | 5.095 |  |
| Sec31a | 7466.9 | 54379.000 | 0.02152 | 7.283 | CTTNBP2NL | 1 | 1 | 5.022 | 5.022 |  |
| Snrnp40 | 6662.0333 | 32255.500 | 0.00608 | 4.842 | H2AC21 | 1 | 1 | 5.004 | 5.004 |  |
| H3 | 16331000 | 57502333.330 | 0.00803 | 3.521 | THADA | 1 | 1 | 4.961 | 4.961 |  |
| Mamdc2 | 35461 | 123173.667 | 0.01069 | 3.473 | EIF3B | 1 | 1 | 4.925 | 4.925 |  |
| Ndufs6 | 22766 | 77644.500 | 0.02794 | 3.411 | PNPT1 | 1 | 1 | 4.898 | 4.898 |  |
| L2hgdh | 72839 | 242905.000 | 0.04549 | 3.335 | YTHDC2 | 1 | 1 | 4.743 | 4.743 |  |
| Tpm4 | 537813.33 | 1727933.333 | 0.02236 | 3.213 | RAN | 1 | 1 | 4.734 | 4.734 |  |
| H4c16 | 27052000 | 86291666.670 | 0.02444 | 3.19 | SYAP1 | 1 | 1 | 4.724 | 4.724 |  |
| Mif | 394793.33 | 1190630.000 | 0.02331 | 3.016 | ZC3H7A | 1 | 1 | 4.614 | 4.614 |  |
| Polr3a | 12937.667 | 37947.500 | 0.00044 | 2.933 | SP3 | 1 | 1 | 4.605 | 4.605 |  |
| Hnrnpa1 | 35293.333 | 87194.333 | 0.03113 | 2.471 | EDC4 | 1 | 1 | 4.554 | 4.554 |  |
| Mbl1 | 205416.67 | 504366.667 | 0.0028 | 2.455 | WDR4 | 1 | 1 | 4.545 | 4.545 |  |
| Ifi202b | 1384833.3 | 3338033.333 | 0.01525 | 2.41 | SURF6 | 1 | 1 | 4.521 | 4.521 |  |
| Flt1 | 25024.667 | 59314.000 | 0.02083 | 2.37 | ACTR2 | 1 | 1 | 4.517 | 4.517 |  |
| H2ac12 | 51091000 | 118433333.300 | 0.00177 | 2.318 | A2ML1 | 1 | 1 | 4.511 | 4.511 |  |
| Pon1 | 37085 | 81669.000 | 0.01977 | 2.202 | CAV1 | 1 | 1 | 4.435 | 4.435 |  |
| Masp1 | 145076.67 | 317996.667 | 0.04533 | 2.192 | ZNF222 | 1 | 1 | 4.403 | 4.403 |  |
| Ssbp1 | 51708.667 | 107813.667 | 0.03544 | 2.085 | PAXX | 1 | 1 | 4.396 | 4.396 |  |
| Elane | 603920 | 1226500.000 | 0.05 | 2.031 | MORF4L1 | 1 | 1 | 4.323 | 4.323 |  |
| C1ra | 265530 | 537470.000 | 0.0499 | 2.024 | MPHOSPH8 | 1 | 1 | 4.252 | 4.252 |  |
| Hist1h2bj | 24219000 | 48973333.330 | 0.00918 | 2.022 | RAB6C | 1 | 1 | 4.211 | 4.211 |  |
| Vim | 173116.67 | 349903.333 | 0.04567 | 2.021 | MRI1 | 1 | 1 | 4.204 | 4.204 |  |

Partial metabolites are listed due to the length limitation. Values represent intensity.

**Supplementary Table 4: Small RNA-seq analysis of 231 EV and 231 -G EV.**

|  | | | | | | | |  |  |
| --- | --- | --- | --- | --- | --- | --- | --- | --- | --- |
| **Mature_ID** | **231 EV-1** | **231-G EV-1** | **FC** | **Mature_ID** | **231 EV-2** | **231-G EV-2** | **FC** | **Upregulated miRNAs in 231-G EV (FC>1.5)** | **Predicted miRNAs targeting AGMAT** |
| *hsa*-miR-206 | 0 | 6283.260 | 6284.260 | *hsa*-miR-188-5p | 0 | 23.4063 | 24.4063 | *hsa*-miR-449c-5p | *hsa*-miR-33b-5p |
| *hsa*-miR-449c-5p | 0 | 3718.664 | 3719.664 | 11_5610 | 0 | 23.4063 | 24.4063 | *hsa*-miR-449a | *hsa*-miR-33a-5p |
| *hsa*-miR-4695-5p | 0 | 1987.562 | 1988.562 | *hsa*-miR-1267 | 0 | 17.5547 | 18.5547 | *hsa*-miR-3158-3p | *hsa*-miR-182-5p |
| *hsa*-miR-375-3p | 0 | 769.379 | 770.379 | *hsa*-miR-320d | 0 | 17.5547 | 18.5547 | *hsa*-miR-1287-5p | *hsa*-miR-96-5p |
| *hsa*-miR-449a | 0 | 769.379 | 770.379 | *hsa*-miR-450a-1-3p | 0 | 17.5547 | 18.5547 | *hsa*-miR-33a-5p | *hsa*-miR-1271-5p |
| *hsa*-miR-6165 | 0 | 641.149 | 642.149 | *hsa*-miR-4668-5p | 0 | 17.5547 | 18.5547 | *hsa*-miR-574-3p | *hsa*-miR-599 |
| *hsa*-miR-3158-3p | 0 | 577.034 | 578.034 | *hsa*-miR-500a-5p | 0 | 17.5547 | 18.5547 | *hsa*-let-7d-3p | *hsa*-miR-320b |
| *hsa*-miR-133a-3p | 0 | 577.034 | 578.034 | *hsa*-miR-6505-3p | 0 | 17.5547 | 18.5547 | *hsa*-miR-1307-5p | *hsa*-miR-320d |
| *hsa*-miR-6718-5p | 0 | 512.919 | 513.919 | *hsa*-miR-7705 | 0 | 17.5547 | 18.5547 | *hsa*-miR-1843 | *hsa*-miR-320a |
| *hsa*-miR-5003-5p | 0 | 512.919 | 513.919 | 5_23418 | 0 | 17.5547 | 18.5547 | *hsa*-miR-629-5p | *hsa*-miR-320c |
| *hsa*-miR-1287-5p | 0 | 384.689 | 385.689 | *hsa*-let-7f-2-3p | 0 | 11.7032 | 12.7032 | *hsa*-miR-19b-1-5p | *hsa*-miR-4429 |
| *hsa*-miR-150-5p | 0 | 384.689 | 385.689 | *hsa*-miR-1266-5p | 0 | 11.7032 | 12.7032 | *hsa*-miR-339-3p | *hsa*-miR-22-3p |
| *hsa*-miR-199b-5p | 0 | 320.574 | 321.574 | *hsa*-miR-19b-1-5p | 0 | 11.7032 | 12.7032 | *hsa*-miR-1278 | *hsa*-miR-6835-3p |
| *hsa*-miR-155-5p | 0 | 320.574 | 321.574 | *hsa*-miR-2276-5p | 0 | 11.7032 | 12.7032 | *hsa*-miR-425-3p |  |
| *hsa*-miR-548as-5p | 0 | 320.574 | 321.574 | *hsa*-miR-3176 | 0 | 11.7032 | 12.7032 | *hsa*-miR-326 |  |
| *hsa*-miR-486-3p | 0 | 320.574 | 321.574 | *hsa*-miR-320e | 0 | 11.7032 | 12.7032 | *hsa*-miR-6780b-5p |  |
| *hsa*-miR-6869-5p | 0 | 320.574 | 321.574 | *hsa*-miR-362-3p | 0 | 11.7032 | 12.7032 | *hsa*-let-7i-3p |  |
| *hsa*-miR-24-2-5p | 0 | 256.460 | 257.460 | *hsa*-miR-3620-3p | 0 | 11.7032 | 12.7032 | *hsa*-miR-7706 |  |
| *hsa*-miR-3189-3p | 0 | 256.460 | 257.460 | *hsa*-miR-3913-5p | 0 | 11.7032 | 12.7032 | *hsa*-miR-130b-3p |  |
| *hsa*-miR-4732-5p | 0 | 256.460 | 257.460 | *hsa*-miR-422a | 0 | 11.7032 | 12.7032 | *hsa*-miR-597-5p |  |
| *hsa*-miR-33a-5p | 0 | 256.460 | 257.460 | *hsa*-miR-4422 | 0 | 11.7032 | 12.7032 | *hsa*-miR-3190-3p |  |
| *hsa*-miR-449b-5p | 0 | 192.345 | 193.345 | *hsa*-miR-4485-5p | 0 | 11.7032 | 12.7032 | *hsa*-miR-3177-3p |  |
| *hsa*-miR-210-3p | 0 | 192.345 | 193.345 | *hsa*-miR-4689 | 0 | 11.7032 | 12.7032 | *hsa*-miR-324-3p |  |
| *hsa*-miR-574-3p | 0 | 192.345 | 193.345 | *hsa*-miR-500b-5p | 0 | 11.7032 | 12.7032 | *hsa*-miR-1266-5p |  |
| *hsa*-miR-5010-5p | 0 | 192.345 | 193.345 | *hsa*-miR-582-5p | 0 | 11.7032 | 12.7032 | *hsa*-miR-148a-3p |  |
| *hsa*-miR-378d | 0 | 192.345 | 193.345 | *hsa*-miR-6756-3p | 0 | 11.7032 | 12.7032 | *hsa*-miR-378c |  |
| *hsa*-miR-1343-5p | 0 | 192.345 | 193.345 | *hsa*-miR-6768-5p | 0 | 11.7032 | 12.7032 | *hsa*-miR-27a-3p |  |
| *hsa*-miR-3978 | 0 | 192.345 | 193.345 | *hsa*-miR-6781-5p | 0 | 11.7032 | 12.7032 | *hsa*-miR-134-5p |  |
| *hsa*-miR-6821-5p | 0 | 192.345 | 193.345 | *hsa*-miR-744-3p | 0 | 11.7032 | 12.7032 | *hsa*-miR-25-3p |  |
| *hsa*-miR-8053 | 0 | 192.345 | 193.345 | 11_5575 | 0 | 11.7032 | 12.7032 | *hsa*-miR-140-3p |  |
| *hsa*-miR-95-3p | 0 | 192.345 | 193.345 | *hsa*-miR-887-3p | 8.8902 | 111.18 | 11.3425 | *hsa*-miR-139-5p |  |
| *hsa*-let-7d-3p | 0 | 192.345 | 193.345 | *hsa*-miR-105-3p | 8.8902 | 76.0705 | 7.79261 | *hsa*-miR-877-5p |  |
| *hsa*-miR-1469 | 0 | 192.345 | 193.345 | *hsa*-miR-320c | 4.4451 | 40.9611 | 7.70621 | *hsa*-miR-130a-3p |  |
| *hsa*-miR-3926 | 0 | 192.345 | 193.345 | *hsa*-miR-101-5p | 0 | 5.8516 | 6.8516 | *hsa*-miR-186-5p |  |
| *hsa*-miR-215-5p | 54.35669 | 9296.660 | 167.959 | *hsa*-miR-10399-5p | 0 | 5.8516 | 6.8516 | *hsa*-miR-423-5p |  |
| *hsa*-miR-223-5p | 0 | 128.230 | 129.230 | *hsa*-miR-10527-5p | 0 | 5.8516 | 6.8516 | *hsa*-miR-29a-3p |  |
| *hsa*-miR-4298 | 0 | 128.230 | 129.230 | *hsa*-miR-1179 | 0 | 5.8516 | 6.8516 | *hsa*-miR-128-3p |  |
| *hsa*-miR-1307-5p | 0 | 128.230 | 129.230 | *hsa*-miR-1251-5p | 0 | 5.8516 | 6.8516 | *hsa*-miR-3131 |  |
| *hsa*-miR-1843 | 0 | 128.230 | 129.230 | *hsa*-miR-1278 | 0 | 5.8516 | 6.8516 | *hsa*-miR-532-5p |  |
| *hsa*-miR-410-5p | 0 | 128.230 | 129.230 | *hsa*-miR-1294 | 0 | 5.8516 | 6.8516 |  |  |
| *hsa*-miR-342-3p | 0 | 128.230 | 129.230 | *hsa*-miR-134-5p | 0 | 5.8516 | 6.8516 |  |  |
| *hsa*-miR-125b-2-3p | 0 | 128.230 | 129.230 | *hsa*-miR-1908-5p | 0 | 5.8516 | 6.8516 |  |  |
| *hsa*-miR-6088 | 0 | 128.230 | 129.230 | *hsa*-miR-196a-3p | 0 | 5.8516 | 6.8516 |  |  |
| *hsa*-miR-4740-5p | 0 | 128.230 | 129.230 | *hsa*-miR-2113 | 0 | 5.8516 | 6.8516 |  |  |
| *hsa*-miR-135b-5p | 0 | 128.230 | 129.230 | *hsa*-miR-26b-3p | 0 | 5.8516 | 6.8516 |  |  |
| *hsa*-miR-5001-5p | 0 | 128.230 | 129.230 | *hsa*-miR-3131 | 0 | 5.8516 | 6.8516 |  |  |
| *hsa*-miR-888-5p | 0 | 128.230 | 129.230 | *hsa*-miR-3174 | 0 | 5.8516 | 6.8516 |  |  |
| *hsa*-miR-6794-5p | 0 | 128.230 | 129.230 | *hsa*-miR-3177-3p | 0 | 5.8516 | 6.8516 |  |  |
| *hsa*-miR-4534 | 0 | 128.230 | 129.230 | *hsa*-miR-3190-3p | 0 | 5.8516 | 6.8516 |  |  |
| *hsa*-miR-483-3p | 0 | 128.230 | 129.230 | *hsa*-miR-3194-5p | 0 | 5.8516 | 6.8516 |  |  |
| *hsa*-miR-3619-5p | 0 | 128.230 | 129.230 | *hsa*-miR-3605-3p | 0 | 5.8516 | 6.8516 |  |  |
| *hsa*-miR-187-3p | 0 | 128.230 | 129.230 | *hsa*-miR-3617-3p | 0 | 5.8516 | 6.8516 |  |  |
| *hsa*-miR-12125 | 0 | 128.230 | 129.230 | *hsa*-miR-378c | 0 | 5.8516 | 6.8516 |  |  |
| *hsa*-miR-3689f | 0 | 128.230 | 129.230 | *hsa*-miR-3940-3p | 0 | 5.8516 | 6.8516 |  |  |
| *hsa*-miR-3064-3p | 0 | 128.230 | 129.230 | *hsa*-miR-3944-3p | 0 | 5.8516 | 6.8516 |  |  |
| *hsa*-miR-629-5p | 0 | 128.230 | 129.230 | *hsa*-miR-4254 | 0 | 5.8516 | 6.8516 |  |  |
| *hsa*-miR-3666 | 0 | 128.230 | 129.230 | *hsa*-miR-449c-5p | 0 | 5.8516 | 6.8516 |  |  |
| *hsa*-miR-29c-5p | 0 | 128.230 | 129.230 | *hsa*-miR-4657 | 0 | 5.8516 | 6.8516 |  |  |

Partial miRNAs are listed due to the length limitation. Values represent standardized expression level.

**Supplementary Table 5: Downregulated peaks proximal genes in 231 -G EV and 231/miR-33a EV treated CAFs from ChIP-seq.**

| **231 EV up peaks proximal gene versus 231/miR-**  **33a EV** | **231 EV up peaks proximal gene versus 231 -G EV** | **Overlap** | |
| --- | --- | --- | --- |
| Baz2b | Aatf | Nop56 | [NOP 56 ribonucleoprotein(Nop56)](https://david.ncifcrf.gov/geneReportFull.jsp?rowids=67134) |
| Ccdc 82 | Abraxas 2 | Phf 21a | [PHD finger protein 21A(Phf21a)](https://david.ncifcrf.gov/geneReportFull.jsp?rowids=192285) |
| Cdk6 | Abtb 1 | Baz2b | [bromodomain adjacent to zinc finger domain, 2B(Baz2b)](https://david.ncifcrf.gov/geneReportFull.jsp?rowids=407823) |
| Cdkn2c | Acaa 2 | Cdk6 | [cyclin-dependent kinase 6(Cdk6)](https://david.ncifcrf.gov/geneReportFull.jsp?rowids=12571) |
| Cldn34d | Acadm | Tia1 | [cytotoxic granule-associated RNA binding protein 1(Tia1)](https://david.ncifcrf.gov/geneReportFull.jsp?rowids=21841) |
| Col 19a1 | Acap 2 | Hspa5 | [heat shock protein 5(Hspa5)](https://david.ncifcrf.gov/geneReportFull.jsp?rowids=14828) |
| Eapp | Acvr 1b | Itln1 | [intelectin 1 (galactofuranose binding)(Itln1)](https://david.ncifcrf.gov/geneReportFull.jsp?rowids=16429) |
| Gm 15319 | Adar | Meioc | [meiosis specific with coiled-coil domain(Meioc)](https://david.ncifcrf.gov/geneReportFull.jsp?rowids=268491) |
| Gm 21119 | Adipor1 | Mllt10 | [myeloid/lymphoid or mixed-lineage leukemia; translocated to, 10(Mllt10)](https://david.ncifcrf.gov/geneReportFull.jsp?rowids=17354) |
| Gm 5458 | Aga | Gm 15319 | [predicted gene 15319 (Gm 15319)](https://david.ncifcrf.gov/geneReportFull.jsp?rowids=100040599) |
| Gtf3c3 | Akirin2 | Gm 21119 | [predicted gene, 21119 (Gm 21119)](https://david.ncifcrf.gov/geneReportFull.jsp?rowids=100861668) |
| Hspa5 | Akt3 | Primpol | primase and polymerase (DNA-directed)(Primpol) |
| Itln1 | Alg8 | Rpl 31-ps 12 [ribosomal protein L31, pseudogene 1 2(Rpl31-ps 12)](https://david.ncifcrf.gov/geneReportFull.jsp?rowids=665562) | |
| Lrrc4c | Alkbh3 | Slc 30a4 | [solute carrier family 30 (zinc transporter), member 4(Slc30a4)](https://david.ncifcrf.gov/geneReportFull.jsp?rowids=22785) |
| Meioc | Als 2 | Tg | [thyroglobulin (Tg)](https://david.ncifcrf.gov/geneReportFull.jsp?rowids=21819) |
| Mllt10 | Ambra 1 | Tnfsf 18 | [tumor necrosis factor (ligand) superfamily, member 18(Tnfsf18)](https://david.ncifcrf.gov/geneReportFull.jsp?rowids=240873) |
| Nop56 | Anapc 5 |  | |
| Phf 21a | Angel2 |  | |
| Primpol | Ankmy 2 |  | |
| Rpl 31-ps 12 | Ankrd11 |  | |
| Six2 | Ankrd26 |  | |
| Slc 30a4 | Ap3b1 |  | |
| Tg | Apcdd1 |  | |
| Tia1 | App |  | |
| Tnfsf 18 | Aptx |  | |
| Tra 2a | Arf 2 |  | |
|  | Arhgap12 |  | |
|  | Arhgap31 |  | |
|  | Arl1 |  | |
|  | Arl15 |  | |
|  | Arl4a |  | |
|  | Arl8a |  | |
|  | Armc 6 |  | |
|  | Armt1 |  | |
|  | Arsa |  | |
|  | Atp5g2 |  | |
|  | Atp5j |  | |
|  | Atp5j2 |  | |
|  | Atp6v0c |  | |
|  | AY074887 |  | |
|  | B 3gat3 |  | |
|  | Baz1b |  | |
|  | Baz2b |  | |
|  | Bbx |  | |
|  | BC 003965 |  | |
|  | Bckdha |  | |
|  | Bclaf 1 |  | |
|  | Bin3 |  | |
|  | Birc6 |  | |
|  | Blmh |  | |
|  | Bmt2 |  | |
|  | Brf1 |  | |
|  | Bri3bp |  | |
|  | Brwd1 |  | |
|  | Btn 2a2 |  | |
|  | Bzw 1 |  | |
|  | C 87436 |  | |
|  | Calm 3 |  | |
|  | Camk1 |  | |

Partial candidates are listed due to the length limitation.

**Supplementary Table 6: Predicted RNA-binding protein and mass spectrum analysis of EVs.**

| **Predicted RNA-binding protein of miR-33a Mass spectrum analysis of 231 EV and 231 -G EV** | | | | | | | | | | | | | | |
| --- | --- | --- | --- | --- | --- | --- | --- | --- | --- | --- | --- | --- | --- | --- |
| **Score** | **Relative**  **score** | **RBP**  **Name** | **Matching sequence** | **Matrix ID** | **Download PWM** | **Download PFM** | **231 EV** | **231 -G EV** | **pvalue** | **fc** | **log2fc** | **regulate** | **significant** | **log10 p**  **value** |
| 7.22942 | 100% | [Pum2](http://rbpdb.ccbr.utoronto.ca/proteins.php?PME_sys_operation=PME_op_View&PME_sys_rec=1800) | UGUA | [329_1178](http://rbpdb.ccbr.utoronto.ca/experiments.php?exp_id=329)  [0640](http://rbpdb.ccbr.utoronto.ca/experiments.php?exp_id=329) | [Downloa d PWM](http://rbpdb.ccbr.utoronto.ca/PWMDir/329_11780640.pwm) | [Downloa d PFM](http://rbpdb.ccbr.utoronto.ca/PFMDir/329_11780640.pfm) | ND | ND |  |  |  |  |  |  |
| 6.241967 | 56% | [A2BP1](http://rbpdb.ccbr.utoronto.ca/proteins.php?PME_sys_operation=PME_op_View&PME_sys_rec=1276) | UGCAUU | [37_16537](http://rbpdb.ccbr.utoronto.ca/experiments.php?exp_id=37)  [540](http://rbpdb.ccbr.utoronto.ca/experiments.php?exp_id=37) | [Downloa](http://rbpdb.ccbr.utoronto.ca/PWMDir/37_16537540.pwm)  [d PWM](http://rbpdb.ccbr.utoronto.ca/PWMDir/37_16537540.pwm) | [Downloa](http://rbpdb.ccbr.utoronto.ca/PFMDir/37_16537540.pfm)  [d PFM](http://rbpdb.ccbr.utoronto.ca/PFMDir/37_16537540.pfm) | ND | ND |  |  |  |  |  |  |
| 4.80838 | 59% | [SNRPA](http://rbpdb.ccbr.utoronto.ca/proteins.php?PME_sys_operation=PME_op_View&PME_sys_rec=1274) | GUUGCA U | [1175_195](http://rbpdb.ccbr.utoronto.ca/experiments.php?exp_id=1175)  [61594](http://rbpdb.ccbr.utoronto.ca/experiments.php?exp_id=1175) | [Downloa](http://rbpdb.ccbr.utoronto.ca/PWMDir/1175_19561594.pwm)  [d PWM](http://rbpdb.ccbr.utoronto.ca/PWMDir/1175_19561594.pwm) | [Downloa](http://rbpdb.ccbr.utoronto.ca/PFMDir/1175_19561594.pfm)  [d PFM](http://rbpdb.ccbr.utoronto.ca/PFMDir/1175_19561594.pfm) | 1 | 2.908 | 0.9172 | 2.908 | 1.540027 | up | yes | -0.03754 |
| 4.559639 | 52% | [a2bp1](http://rbpdb.ccbr.utoronto.ca/proteins.php?PME_sys_operation=PME_op_View&PME_sys_rec=2570) | GCAUU | [36_12574](http://rbpdb.ccbr.utoronto.ca/experiments.php?exp_id=36)  [126](http://rbpdb.ccbr.utoronto.ca/experiments.php?exp_id=36) | [Downloa d PWM](http://rbpdb.ccbr.utoronto.ca/PWMDir/36_12574126.pwm) | [Downloa d PFM](http://rbpdb.ccbr.utoronto.ca/PFMDir/36_12574126.pfm) | ND | ND |  |  |  |  |  |  |
| 3.313818 | 45% | [ACO1](http://rbpdb.ccbr.utoronto.ca/proteins.php?PME_sys_operation=PME_op_View&PME_sys_rec=2664) | CAUUGC | [1213_802](http://rbpdb.ccbr.utoronto.ca/experiments.php?exp_id=1213)  [1254](http://rbpdb.ccbr.utoronto.ca/experiments.php?exp_id=1213) | [Downloa](http://rbpdb.ccbr.utoronto.ca/PWMDir/1213_8021254.pwm)  [d PWM](http://rbpdb.ccbr.utoronto.ca/PWMDir/1213_8021254.pwm) | [Downloa](http://rbpdb.ccbr.utoronto.ca/PFMDir/1213_8021254.pfm)  [d PFM](http://rbpdb.ccbr.utoronto.ca/PFMDir/1213_8021254.pfm) | 1 | 1.354 | 1 | 1.354 | 0.437228 | no change | yes | 0 |
| 3.169925 | 50% | [KHSRP](http://rbpdb.ccbr.utoronto.ca/proteins.php?PME_sys_operation=PME_op_View&PME_sys_rec=2661) | GUGC | [1186_178](http://rbpdb.ccbr.utoronto.ca/experiments.php?exp_id=1186)  [93325](http://rbpdb.ccbr.utoronto.ca/experiments.php?exp_id=1186) | [Downloa](http://rbpdb.ccbr.utoronto.ca/PWMDir/1186_17893325.pwm)  [d PWM](http://rbpdb.ccbr.utoronto.ca/PWMDir/1186_17893325.pwm) | [Downloa](http://rbpdb.ccbr.utoronto.ca/PFMDir/1186_17893325.pfm)  [d PFM](http://rbpdb.ccbr.utoronto.ca/PFMDir/1186_17893325.pfm) | 1 | 1.08 | 1 | 1.08 | 0.111031 | no change | yes | 0 |
| 3.141731 | 50% | [YTHDC1](http://rbpdb.ccbr.utoronto.ca/proteins.php?PME_sys_operation=PME_op_View&PME_sys_rec=1283) | CAUUGC | [969_2016](http://rbpdb.ccbr.utoronto.ca/experiments.php?exp_id=969)  [7602](http://rbpdb.ccbr.utoronto.ca/experiments.php?exp_id=969) | [Downloa](http://rbpdb.ccbr.utoronto.ca/PWMDir/969_20167602.pwm)  [d PWM](http://rbpdb.ccbr.utoronto.ca/PWMDir/969_20167602.pwm) | [Downloa](http://rbpdb.ccbr.utoronto.ca/PFMDir/969_20167602.pfm)  [d PFM](http://rbpdb.ccbr.utoronto.ca/PFMDir/969_20167602.pfm) | ND | ND |  |  |  |  |  |  |
| 2.846915 | 64% | [ELAVL1](http://rbpdb.ccbr.utoronto.ca/proteins.php?PME_sys_operation=PME_op_View&PME_sys_rec=1258) | GUUG | [1170_195](http://rbpdb.ccbr.utoronto.ca/experiments.php?exp_id=1170)  [61594](http://rbpdb.ccbr.utoronto.ca/experiments.php?exp_id=1170) | [Downloa](http://rbpdb.ccbr.utoronto.ca/PWMDir/1170_19561594.pwm)  [d PWM](http://rbpdb.ccbr.utoronto.ca/PWMDir/1170_19561594.pwm) | [Downloa](http://rbpdb.ccbr.utoronto.ca/PFMDir/1170_19561594.pfm)  [d PFM](http://rbpdb.ccbr.utoronto.ca/PFMDir/1170_19561594.pfm) | 1 | 0.781 | 1 | 0.781 | -0.35661 | no change | yes | 0 |
| 1.866248 | 40% | [SFRS1](http://rbpdb.ccbr.utoronto.ca/proteins.php?PME_sys_operation=PME_op_View&PME_sys_rec=1448) | UGCA | [1173_195](http://rbpdb.ccbr.utoronto.ca/experiments.php?exp_id=1173)  [61594](http://rbpdb.ccbr.utoronto.ca/experiments.php?exp_id=1173) | [Downloa d PWM](http://rbpdb.ccbr.utoronto.ca/PWMDir/1173_19561594.pwm) | [Downloa d PFM](http://rbpdb.ccbr.utoronto.ca/PFMDir/1173_19561594.pfm) | 1 | 1.541 | 1 | 1.541 | 0.623867 | up | yes | 0 |
| 1.866248 | 40% | [SFRS1](http://rbpdb.ccbr.utoronto.ca/proteins.php?PME_sys_operation=PME_op_View&PME_sys_rec=1448) | UGCA | [1173_195](http://rbpdb.ccbr.utoronto.ca/experiments.php?exp_id=1173)  [61594](http://rbpdb.ccbr.utoronto.ca/experiments.php?exp_id=1173) | [Downloa](http://rbpdb.ccbr.utoronto.ca/PWMDir/1173_19561594.pwm)  [d PWM](http://rbpdb.ccbr.utoronto.ca/PWMDir/1173_19561594.pwm) | [Downloa](http://rbpdb.ccbr.utoronto.ca/PFMDir/1173_19561594.pfm)  [d PFM](http://rbpdb.ccbr.utoronto.ca/PFMDir/1173_19561594.pfm) | 1 | 1.541 | 1 | 1.541 | 0.623867 | up | yes | 0 |

**Supplementary Table 7: Clinical characteristics of human specimens.**

| **Pathological number** | **Number** | **Types** | **Grade** | **Subtypes** | **Tumor size** | **Age** |
| --- | --- | --- | --- | --- | --- | --- |
| 201611128-16 | 1 | Invasive breast cancer | WHO II | Luminal B | 4*2*2cm | 45 |
| 201611018-12 | 2 | Invasive ductal carcinoma | WHO II | HER-2 | 2.5*2*1cm | 49 |
| 201610840-8 | 3 | Invasive ductal carcinoma | WHO II | Luminal B | 2.5*2.5*1.5cm | 41 |
| 201702945-3 | 4 | Invasive ductal carcinoma | WHO III | TNBC | 2.5×1.5cm | 64 |
| 201600501-3 | 5 | Invasive ductal carcinoma with central necrosis and interstitial massive lymphocyte infiltration | WHO III | HER-2 or TNBC | max diameter 2.5cm | 67 |
| 201608462-4 | 6 | Invasive ductal carcinoma | WHO III | Luminal B | max diameter 2.5cm | 46 |
| 201615818-2 | 7 | Ductal carcinoma in situ |  | HER-2 or TNBC | 3.5*2cm | 74 |
| 201607187-1 | 8 | Invasive ductal carcinoma | WHO II | Luminal A | max diameter 1.5cm | 59 |
| 201607806-40 | 9 | Ductal carcinoma in situ |  | HER-2 | 4*3.5*1.5 | 52 |
| 201620092-3 | 10 | Invasive ductal carcinoma | WHO III | Luminal B | 3*3*2.5cm | 52 |
| 201623528-6 | 11 | Invasive breat carcinoma with neuroendocrine features |  | Luminal A |  | 61 |
| 201615698-7 | 12 | Invasive ductal carcinoma | WHO II | Luminal B | max diameter 2.5cm | 64 |
| 201602435-1 | 13 | Invasive ductal carcinoma | WHO II | Luminal B |  | 48 |
| 201614684-14 | 14 | Ductal carcinoma in situ |  | - |  | 36 |
| 201603732-5 | 15 | Invasive ductal carcinoma | WHO II-III | Luminal A | max diameter 4cm | 75 |
| 201615795-7 | 16 | Invasive ductal carcinoma | WHO III | HER-2 |  | 37 |
| 201607955-3 | 17 | Invasive ductal carcinoma | WHO III | Luminal B | max diameter 2.3cm | 68 |
| 201619157-3 | 18 | Invasive micropapillary carcinoma | WHO III | HER-2 |  | 40 |
| 201614885-2 | 19 | Invasive ductal carcinoma | WHO II | HER-2 |  | 49 |
| 201616112-1 | 20 | Invasive ductal carcinoma | WHO III | Luminal B | max diameter 3.5cm | 48 |
| 201617129-1 | 21 | Invasive ductal carcinoma | WHO II | Luminal A |  | 49 |
| 201618645-3 | 22 | Ductal carcinoma in situ |  | HER-2 |  | 57 |
| 201600982-3 | 23 | Invasive ductal carcinoma | WHO II | Luminal B | max diameter 0.7cm | 46 |
| 201607954-8 | 24 | Invasive ductal carcinoma | WHO II-III | Luminal B | max diameter 5cm | 49 |
| 201607681-6 | 25 | Invasive ductal carcinoma | WHO II | Luminal A |  | 64 |
| 201624184-14 | 26 | Ductal carcinoma in situ |  | HER-2 | max diameter 0.6cm | 61 |
| 201614269-5 | 27 | Invasive ductal carcinoma | WHO II | Luminal A |  | 54 |
| 201608493-11 | 28 | Invasive micropapillary carcinoma |  | Luminal B | 4.5*4*3cm | 33 |
| 201621259-3 | 29 | Invasive ductal carcinoma | WHO III | HER-2 | max diameter 2cm | 73 |
| 201608111-13 | 30 | Invasive ductal carcinoma | WHO II | Luminal A |  | 52 |
| 201618088-7 | 31 | Ductal carcinoma in situ |  | Luminal A | 2.5*1.6*1.5cm | 45 |
| 201618495-1 | 32 | Invasive ductal carcinoma | WHO II | Luminal A | max diameter 1.4cm | 39 |
| 201709181-14 | 33 | Invasive ductal carcinoma | WHO III | Luminal B | 5×4×3cm | 60 |
| 201605898-3 | 34 | Invasive ductal carcinoma | WHO II | HER-2 |  | 46 |
| 201723678-5 | 35 | Invasive ductal carcinoma | WHO II | Luminal B | max diameter 2.5cm | 35 |
| 201606093-2 | 36 | Invasive ductal carcinoma | WHO III | Luminal B | max diameter 1.8cm | 51 |
| 201628844-5 | 37 | Invasive ductal carcinoma | WHO III | HER-2 | max diameter 2cm | 58 |
| 201610236-3 | 38 | Invasive ductal carcinoma | WHO II | Luminal A |  | 39 |
| 201609996-4 | 39 | Invasive ductal carcinoma | WHO II-III | Luminal B |  | 44 |
| 201623227-3 | 40 | Invasive ductal carcinoma | WHO III | HER-2 | max diameter 2cm | 54 |
| 201621448-5 | 41 | Invasive ductal carcinoma | WHO II | Luminal A | max diameter 2cm | 61 |
| 201636232-2 | 42 | Ductal carcinoma in situ |  | Luminal A | max diameter 3cm | 48 |
| 201606001-10 | 43 | Invasive ductal carcinoma | WHO II | TNBC |  | 47 |
| 201718292-4 | 44 | Invasive ductal carcinoma | WHO III | Luminal B | 3.5*2.5*1.6cm | 45 |
| 201618645-5 | 45 | Ductal carcinoma in situ |  | HER-2 | max diameter 1.3cm | 57 |
| 201607158-4 | 46 | Invasive ductal carcinoma | WHO III | Luminal A | 3.5*3*2cm | 62 |
| 201725468-12 | 47 | Invasive ductal carcinoma | WHO III | Luminal B | 2*1*1cm | 38 |
| 201621672-7 | 48 | Invasive ductal carcinoma | WHO III | Luminal A |  | 36 |
| 201712907-3 | 49 | Mucinous carcinoma with ductal carcinoma in situ |  | Luminal A | max diameter 1.5cm | 44 |
| 201616031-11 | 50 | Invasive ductal carcinoma | WHO II | HER-2 | max diameter 8cm | 47 |
| 201719549-15 | 51 | Invasive ductal carcinoma | WHO II | Luminal A | 6*4*3cm | 38 |
| 201612540-8 | 52 | Invasive ductal carcinoma | WHO III | HER-2 | max diameter 4cm | 55 |
| 201601542-2 | 53 | Invasive ductal carcinoma | WHO III | Luminal B | max diameter 1.5cm | 55 |
| 201617404-7 | 54 | Invasive ductal carcinoma | WHO II | Luminal A | 6*5*5cm | 57 |
| 201608752-10 | 55 | Invasive ductal carcinoma | WHO II | Luminal A | 2×1.5cm | 69 |
| 201720110-6 | 56 | Invasive lobular carcinoma |  | TNBC | max diameter 1.6cm | 46 |
| 201726333-3 | 57 | Invasive ductal carcinoma | WHO III | Luminal B | max diameter 3cm | 62 |
| 201607191-6 | 58 | Invasive ductal carcinoma | WHO III | HER-2 | 3*2cm | 56 |
| 201619826-8 | 59 | Invasive micropapillary carcinoma |  | Luminal A | 1*1cm | 39 |
| 201601669-10 | 60 | Invasive lobular carcinoma |  | Luminal B |  | 59 |
| 201607445-6 | 61 | Invasive ductal carcinoma | WHO III | HER-2 | max diameter 4cm | 51 |
| 201627711-5 | 62 | Invasive ductal carcinoma | WHO III | Luminal A |  | 35 |
| 201617756-16 | 63 | Paget's disease of the breast |  | HER-2 |  | 52 |
| Fresh samples for western blots and RT-qPCR detection | | |  |  |  |  |
| Hospitalization number | Number | Types | Grade | Subtypes | Age |  |
| 10170776 | 1 | Ductal carcinoma in situ | II | Luminal B | 74 |  |
| 10235604 | 2 | Invasive breast cancer | WHO II | HER-2 | 43 |  |
| 10158119 | 3 | Invasive ductal carcinoma | II | HER-2 | 59 |  |
| 10158839 | 4 | Invasive ductal carcinoma | III | HER-2 | 72 |  |
| 10169431 | 5 | Invasive breast cancer | III | Luminal A | 46 |  |
| 10159793 | 6 | Invasive ductal carcinoma | III | Luminal B | 42 |  |
| 10158829 | 7 | Invasive ductal carcinoma | III | Luminal B | 62 |  |
| 10172024 | 8 | Invasive ductal carcinoma | III | Luminal A | 58 |  |
| 10177975 | 9 | Invasive breast cancer | III | HER-2 | 62 |  |
| 10174886 | 10 | Invasive ductal carcinoma | III | Luminal A | 69 |  |
